# Landscape genomics reveal that ecological character determines adaptation: a case study in smoke tree (*Cotinus coggygria* Scop.)

**DOI:** 10.1186/s12862-017-1055-3

**Published:** 2017-08-23

**Authors:** Cai-Yun Miao, Yong Li, Jie Yang, Run-Li Mao

**Affiliations:** College of Forestry, Henan Agricultural University, Zhengzhou, 450002 China

**Keywords:** Adaptation, *Cotinus coggygria*, Ecological character, Landscape genomics, SCoT marker

## Abstract

**Background:**

The adaptive evolution of species response to environment are the key issues in molecular ecology and evolutionary biology. The direction of adaptive differentiation of species in regions lacking strong selection pressure is usually diverse. However, the driving mechanism of the diverse adaptive differentiation for regional species is still undetermined to date. In this study, we used landscape genomics modelling to infer the adaptive evolution of *Cotinus coggygria* in China’s warm-temperate zone.

**Results:**

Using fifteen natural populations and nine start codon targeted (SCoT) markers, a total of 1131 unambiguous loci were yielded. Our results showed two genetic groups existed in the fifteen natural populations of *C. coggygria,* which is due to the divergent selection driven by six environmental factors. Environmental association analyses revealed the environmental variables related to precipitation were associated with high numbers of environment-associated loci.

**Conclusions:**

Our results indicated that the ecological characters of *C. coggygria*, i.e. avoiding wetness and tolerating drought, determine its adaptive evolution. This study provides a reference that ecological character determines the adaptive evolution of species in regions lacking strong selection pressure.

**Electronic supplementary material:**

The online version of this article (10.1186/s12862-017-1055-3) contains supplementary material, which is available to authorized users.

## Background

The adaptive evolution of species response to environment are the fundamental issues in molecular ecology and evolutionary biology. The global climate is changing rapidly and affecting the global ecosystem and biodiversity. Under the pressure of climate change, species either adapt or become extinct [1]. Species adaptation response to rapid climate change can be divided into two. On the one hand, species adapt through migration to adjust the distribution [2]. On the other hand, for species that cannot adapt to climate change through migration, they resort to local adaptation. Thus, these species require adaptive changes, especially in terms of phenology, reproductive behavior, and phenotypic characteristics [3]. Adaptive phenotypic changes for fitting to the changing climate are usually based on the adaptive evolution of species genome [4]. Therefore, the identification of these adaptive genes is the key in understanding species adaptive evolution.

To date, two strategies can be used to identify the adaptive genes. One is the “top-down” strategy, which measures the adaptive phenotype and phenological data using common garden experiment or reciprocal transplant experiment; then, this strategy links these data to genetic variation via genome-wide association studies [5] or quantitative trait locus [6]. The other is the “bottom-up” strategy, which searches the selected signals of adaptive genetic evolution using genomics scanning and then associates these signals with climatic data to determine the adaptive genes [4]. An example of the latter strategy is landscape genomics, which is less cost and more time efficient than the methods belonging to the former strategy [7, 8].

In recent years, landscape genomics studies have provided information on the interactions between environmental variations and adaptive genetic variations in natural populations [9]. The universal pattern of adaptive evolution is especially popular for regional landscape genomics studies. However, the universal pattern on adaptation needs extreme selection pressure, such as drought of desert and high salinity of ocean, for most species in the same region [10, 11]. However, the strong selection pressure produced by extreme environmental conditions does not exist in most regions. Thus, the direction of adaptive differentiation of regional species is usually diverse [9, 12]. However, the determinant of the diverse adaptive differentiation for regional species is still unclear to date. Determining the reason why these environmental variables drive the comprehensive adaptive differentiation of species genomes is interesting.

In this study, we sampled *Cotinus coggygria* Scop. (smoke tree) in China’s warm-temperate zone to infer the relationship between environmental variables and adaptive genetic variations in the plant’s genome. This deciduous tree species is widely distributed in China’s warm-temperate zone. This species prefers light; tolerates semi-shade, cold, and drought; and avoids wetness. Although previous population genetics study on *C. coggygria* had been conducted [13], the used neutral markers of chloroplast DNA (cpDNA) reflected more demography history events rather than adaptive evolution driving by environmental variations. Here, novel molecular markers, start codon targeted (SCoT) polymorphisms, were used for genome scanning. Start codon targeted polymorphism (SCoT) is a kind of gene targeted marker that was developed based on the conserved region flanking the ATG start codon [14]. SCoT markers have certain advantages, such as simplicity, reproducibility, abundant polymorphism, high throughput and not require priori genomic information [15]. Therefore, this marker is suitable for landscape genomics research.

We employed SCoT markers to detect environment-associated loci (EAL) in response to environmental variations in natural populations of *C. coggygria.* The present study aimed (i) to reveal the population genetic structure of *C. coggygria*, (ii) identify the EAL in the genome of *C. coggygria*, and (iii) detect the key environmental factors that drive the adaptive differentiation of *C. coggygria.*

## Results

### Genetic structure

Nine SCoT primers were selected in investigating the population genetic structure in *C. coggygria*. A total of 1131 unambiguous loci were identified with sizes ranging from 60 bp to 1000 bp. The numbers of loci of the nine primers ranged from 93 (SCoT31) to 163 (SCoT2). The number of polymorphic alleles (*N*_A_) of each population ranged from 134 (P9) to 325 (P14). The percentage of polymorphic alleles (*PPA*) of each population ranged from 11.8 (P9) to 28.7 (P14). The level of genetic diversity (*H*_E_) of each population ranged from 0.038 (P9) to 0.098 (P4). Summary statistics for the genetic diversity analyses for each population are shown in Table 1.

**Table 1:** Details of population locations, sample size, genetic diversity of 15 population for *C. coggygria*

Population no. and code	Locations	Altitude (meter)	Lat.(N)/ Long.(E)	***N***	*N*_A_	*PPA*	*H*_E_
1.HBWD	Wudang Mt., Hubei	988	32.40/111.00	5	141	12.5	0.047
2.HNSM	Song Mt., Henan	631	34.47/113.08	12	274	24.2	0.095
3.SDBD	Baodugu, Shandong	287	35.00/117.70	12	241	21.3	0.083
4.HNJL	Jiulian Mt., Henan	755	35.58/113.58	12	303	26.8	0.098
5.SDYM	Yuan Mt., Shandong	241	36.47/117.85	12	232	20.5	0.087
6.SXLK	Lingkong Mt., Shanxi	1673	36.60/112.08	11	254	22.5	0.070
7.HNLJ	Laojun Mt., Henan	835	33.75/111.63	12	252	22.3	0.074
8.SXTB	Taibai Mt., Shaanxi	3269	33.95/107.75	12	154	13.6	0.049
9.SXTT	Tiantai Mt., Shaanxi	1167	34.28/107.18	11	134	11.8	0.038
10.SXLJ	Laojun Mt., Shaanxi	1241	34.33/110.25	12	261	23.1	0.072
11.SXWL	Wulaofeng, Shanxi	1191	34.83/110.58	9	220	19.5	0.048
12.HNYT	Yuntai Mt., Henan	297	35.42/113.42	12	166	14.7	0.052
13.SXHM	Hua Mt., Shaanxi	1160	35.55/110.10	8	256	22.6	0.059
14.SXTL	Wuzhi Mt., Hebei	793	37.70/112.43	10	325	28.7	0.068
15.HBTG	Tianlong Mt., Shanxi	612	38.25/113.73	6	206	18.2	0.059

*N*_A_ number of polymorphic alleles, *PPA* percentage of polymorphic alleles; *H*_E_, Nei’s (1973) measure of gene diversity

**Fig. 1:**
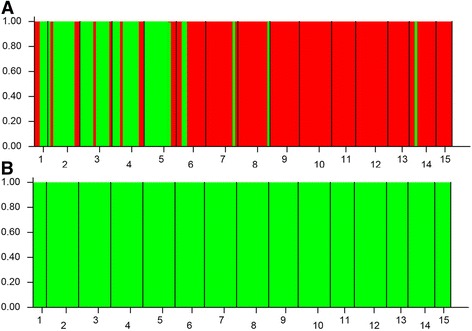
STRUCTURE analyses of fifteen sampled populations of *C. coggygria*. a Population genetic structure estimated by STRUCTURE analysis with all SCoT loci. b Population genetic structure estimated by STRUCTURE analysis with all SCoT loci except EAL. Each vertical bar represents an individual and its assignment proportion into one of two population clusters (*K*)

**Fig. 2:**
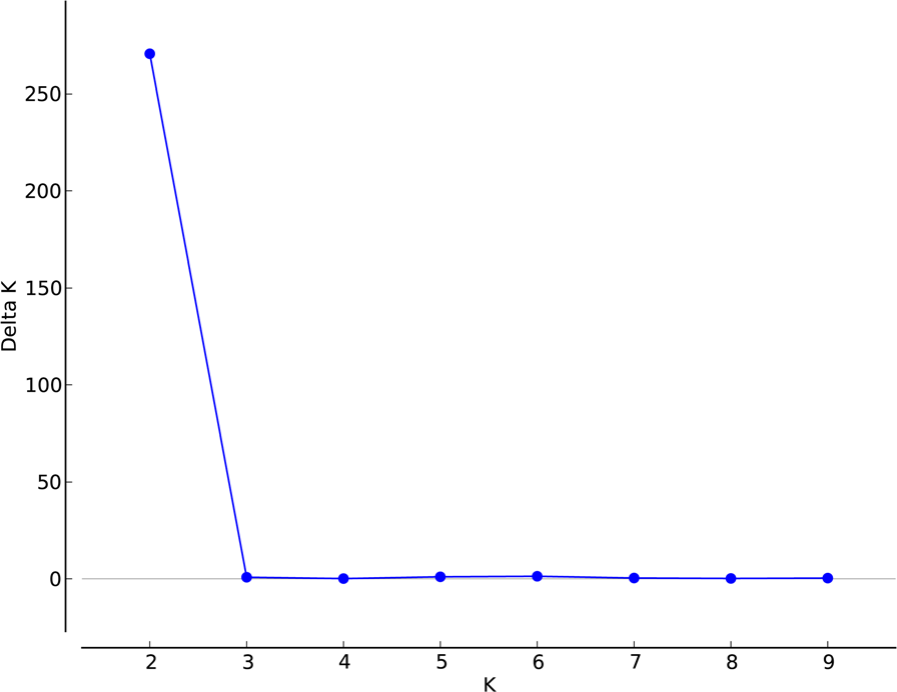
The uppermost hierarchical level of genetic structure determined using values of *ΔK*. *ΔK was computed by* software Structure Harvester

**Fig. 3:**
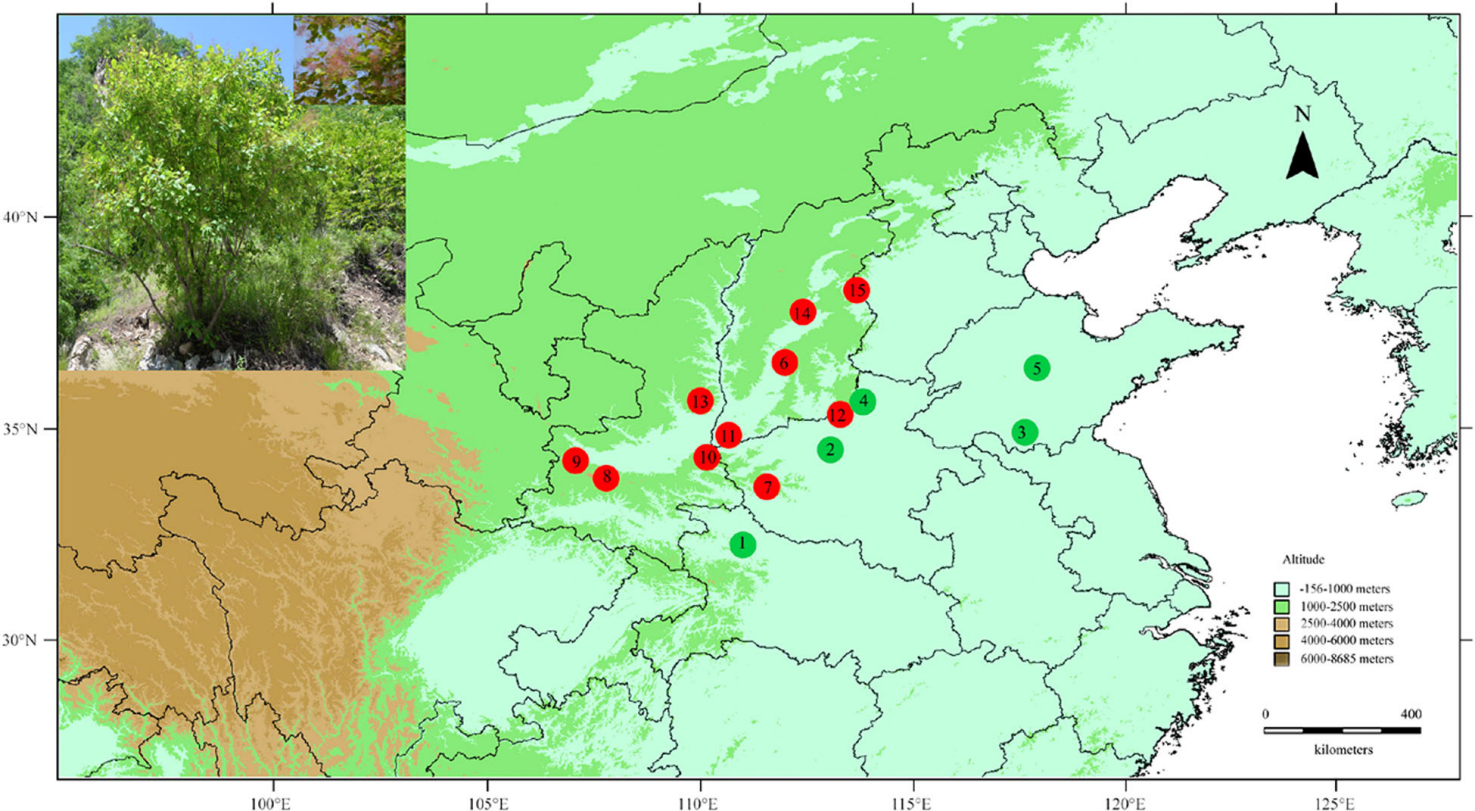
Locations of the fifteen sampled *C. coggygria* populations. Map produced by software DIVA-GIS. The elevation layer file was downloaded from http://www.diva-gis.org/

The Bayesian analysis with all loci of population structure (Fig. 1a) clearly demonstrated that the highest *ΔK* value (Fig. 2) was obtained when populations were clustered into two groups. One is the East group (P1 to P5), the other is the West group (P6 to P15) (Fig. 3). The non-hierarchical AMOVA (Table 2) revealed that these populations were significantly structured at the species-range scale (*F*_ST_ = 0.115, *P* < 0.001). Although two groups were divided, only 5.30% genetic variation occurred among groups (*F*_CT_ = 0.053, *P* < 0.05) and most genetic variation occurred within populations (86.06%, *F*_ST_ = 0.139, *P* < 0.001). In addition, significant patterns of isolation by distance were detected by comparing *F*_ST_ values with geographical distances at the species-range scale (*r* = 0.2452, *P* < 0.05).

**Table 2:** Hierarchical AMOVAs for SCOT variation surveyed in *C. coggygria*

Source of variation	d.f.	%Total variance	*F*-statistic	*P*-value
Non-hierarchical AMOVAs				
Total	14	11.52%	*F*_ST_ = 0.115	*P* < 0.001
East group	4	5.34%	*F*_ST_ = 0.053	*P* < 0.001
West group	9	11.73%	*F*_ST_ = 0.117	*P* < 0.001
Hierarchical AMOVAs				
Among two groups	1	5.30%	*F*_CT_ = 0.053	*P* < 0.05
Among populations	13	8.65%	*F*_SC_ = 0.091	*P* < 0.001
Within populations	141	86.06%	*F*_ST_ = 0.139	*P* < 0.001

### Redundancy analysis

**Fig. 4:**
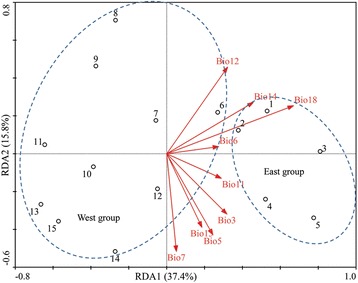
RDA analysis was performed to determine the relative contribution of environmental variations shaping the genetic structure. The biplot depicts the eigenvalues and lengths of eigenvectors for the RDA. Population locations on the spatial axes are marked by their number

A total of 15 natural populations of *C. coggygria* and environmental variables were used as subjects and explanatory variables to perform redundancy analysis (RDA). To avoid overestimation of the contribution of environmental variables to population structure, the strong correlated environmental variables were excluded from 19 environmental variables (Table 3). After removing the strong correlated environmental variables, nine remaining environmental variables (Bio3, Bio5, Bio6, Bio7, Bio11, Bio12, Bio14, Bio15 and Bio18) were selected for RDA and environmental association analyses. Figure 4 shows the results of RDA performed using 1131 SCoT allele frequencies as response variables. Correlations of genetic variables with environmental variables in axes 1 and 2 were 0.934 and 0.965, respectively. The ratios of the total eigenvalues of axes 1 and 2 were 37.4% and 15.8%, respectively. RDA showed that six environmental variables were significantly associated with RDA axes 1 and 2 (Table 4), which suggested that the two axes represented more of the changes in the six environmental variables. Among these six environmental variables, isothermality (Bio3), max temperature of warmest month (Bio5), and temperature annual range (Bio7) were related to temperature. Meanwhile, annual precipitation (Bio12), precipitation seasonality (Bio15), and precipitation of warmest quarter (Bio18) were related to precipitation. Bio18 was the highest contributor among the nine environmental variables because of the high ratios of the total eigenvalues of axis 1.

**Table 3:** Nineteen environmental variables used in this study

Temperature (period 1950–2000)	Bio1: Annual mean temperature (°C × 10)
	Bio2: Mean diurnal range (Mean of monthly (max temp - min temp))
	Bio3: Isothermality (Bio2/Bio7) (×100)
	Bio4: Temperature seasonality (standard deviation ×100)
	Bio5: Max temperature of warmest month (°C × 10)
	Bio6: Min temperature of coldest month (°C × 10)
	Bio7: Temperature annual range (E5-E6)
	Bio8: Mean temperature of wettest quarter (°C × 10)
	Bio9: Mean temperature of driest quarter (°C × 10)
	Bio10: Mean temperature of warmest quarter (°C × 10)
	Bio11: Mean temperature of coldest quarter (°C × 10)
Precipitation (period 1950–2000)	Bio12: Annual precipitation (mm)
	Bio13: Precipitation of wettest month (mm)
	Bio14: Precipitation of driest month (mm)
	Bio15: Precipitation seasonality (coefficient of variation)
	Bio16: Precipitation of wettest quarter (mm)
	Bio17: Precipitation of driest quarter (mm)
	Bio18: Precipitation of warmest quarter (mm)
	Bio19: Precipitation of coldest quarter (mm)

**Table 4:** Correlations between environmental variables and the ordination axes

Environmental variable	Axis 1	Axis 2	Axis 3	Axis 4
Bio3	0.327	−0.523 ^*^	0.436	0.475
Bio5	0.251	−0.702 ^**^	−0.082	0.065
Bio6	0.283	0.062	−0.459	−0.303
Bio7	0.053	−0.846 ^**^	0.282	0.321
Bio11	0.297	−0.213	−0.332	−0.185
Bio12	0.331	0.750 ^**^	−0.270	−0.257
Bio14	0.471	0.444	−0.166	−0.403
Bio15	0.192	−0.637 ^**^	−0.063	0.510 ^*^
Bio18	0.689 ^**^	0.415	−0.323	−0.071

Statistically significant correlation by ^*^ (*P* < 0.05) and ^**^ (*P* < 0.01)

### Outlier detection

Using the FDIST2 method, 100 outlier loci with a *P*-value under 0.05 were identified (Additional file 1: Figure S5A). The BayeScan method detected 74 loci as outliers with a log_10_PO above 0.5; this value is considered a substantial evidence for selection and corresponds to a posterior probability above 0.76 (Additional file 1: Figure S5A). Among the 74 loci detected using the second approach, 27 common loci were detected using the first approach (Additional file 1: Figure S5A). The detected outlier loci all showed a positive alpha value, thereby indicating positive or directional selection. To reduce the false discovery rate, we used the 27 common loci for further environmental association analyses.

### Environmental association

**Fig. 5:**
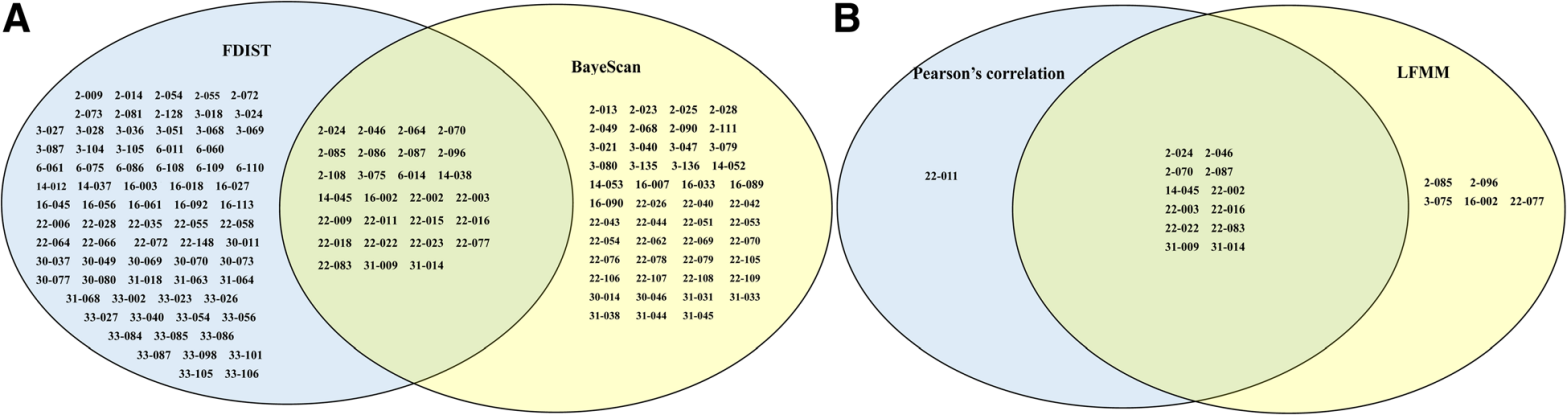
Number summary of outlier loci and EAL. a One hundred, 74, and 27 loci were detected as outlier loci in *C. coggygria* using Bayescan, Dfdist, and both with Dfdist and Bayescan, respectively. b Thirteen, 17, and 12 loci were detected as EAL in *C. coggygria* using Pearson’s correlation, LFMM, and both with Pearson’s correlation and LFMM, respectively

The Pearson’s correlation analysis detected thirteen EAL among the 27 outlier loci, which associated with at least one environmental variable (Table 5). This number accounted to 1.14% of the total number of SCoT loci. Among the 13 detected loci, three were significantly related to temperature and precipitation, one was significantly related to temperature, and nine were significantly related to precipitation (Table 5). Among these associated environmental variables, Bio14 and Bio18 were associated with the highest numbers of EAL. LFMM identified seventeen EAL among the 27 outlier loci, which associated with at least one environmental variable (Table 6). This number accounted to 1.50% of the total number of SCoT loci. Among the 17 detected loci, seven were significantly associated with temperature and precipitation, and ten were significantly associated with precipitation (Table 6). Similar to the Pearson’s correlation analysis, LFMM showed that Bio14 and Bio18 were associated with the highest numbers of EAL. However, Bio15 was also identified as environmental variable with high numbers of EAL. A total of 27 EAL with 12 common EAL were detected using the combined two detection methods (Fig. 5b). To further test the contribution of environmental variables to the spatial genetic structure, the Bayesian analysis with all loci except EAL of population structure was performed. After removing the EAL, the two genetic groups also dissolved (Fig. 1b).

**Table 5:** The EAL as indicated by Pearson’s correlation coefficients

	Pearson’s correlation coefficients								
Locus code	Bio3	Bio5	Bio6	Bio7	Bio11	Bio12	Bio14	Bio15	Bio18
2–024			0.536^*^						0.713^**^
2–046									0.607^*^
2-064									
2–070									0.643^**^
2–085									
2–086									
2–087								0.584^*^	
2-096									
2–108									
3–075									
6–014									
14–038									
14–045							0.570^*^		0.675^**^
16-002									
22–002							0.570^*^		0.622^*^
22-003							0.595^*^		0.658^**^
22-009									
22–011							0.526^*^		
22-015									
22–016							0.531^*^		0.602^*^
22-018									
22–022							0.587^*^		0.584^*^
22-023									
22–077									
22–083	−0.720^**^								
31–009				0.533^*^		−0.732^**^	−0.635^*^		−0.773^**^
31-014				0.578^*^		−0.650^**^	−0.581^*^		−0.569^*^

^*^, *P* < 0.05; ^**^, *P* < 0.01

**Table 6:** The EAL as indicated by |z|-score

Locus code	|z|-score								
	Bio3	Bio5	Bio6	Bio7	Bio11	Bio12	Bio14	Bio15	Bio18
2–024			4.131^***^		3.954^***^		3.487^***^		6.657^***^
2–046		3.652^***^						3.765^***^	4.653^***^
2–064									
2–070								3.774^***^	5.195^***^
2–085								4.172^***^	3.966^***^
2–086									
2–087		4.258^***^		3.191^***^				4.569^***^	
2–096								3.737^***^	3.994^***^
2–108									
3–075									3.949^***^
6–014									
14–038									
14–045							3.981^***^		5.754^***^
16–002	5.970^***^		4.952^***^	4.517^***^				3.798^***^	
22–002							3.644^***^		4.689^***^
22–003							4.039^***^		5.105^***^
22–009									
22–011									
22–015									
22–016									4.722^***^
22–018									
22–022							3.406^***^		3.871^***^
22–023									
22–077									4.168^***^
22–083	8.429^***^	5.581^***^		4.424^***^	4.021^***^			4.964^***^	3.309^***^
31–009				5.021^***^		8.265^***^	7.486^***^		9.638^***^
31–014				4.176^***^		5.174^***^	4.682^***^		4.961^***^

Statistically significant correlation by ^***^ (*P* < 0.001)

## Discussion

This study analyzed the adaptive evolution of *C. coggygria* to environmental factors through SCoT markers. The overall outlier detection rates in the present study were 8.84% (100 out of 1131) in FDIST and 6.54% in BayeScan. The detection rates are consistent with previously reported rates on landscape genomics studies (2.85% to 10%), such as 2.85% in *Alnus glutinosa* [16] and 4.5% in *Picea mariana* [17] from SNPs, 9% in *Arabis alpina* [18] and 10% in 13 alpine species [12] from AFLPs, and 4.22% in *Cephalotaxus oliveri* [19] from ISSRs. Although SCoT markers are non-neutral biased nature, their detection rate in this study is not significantly higher than that of other molecular markers. Due to lacking DNA sequence information, these loci could not be validated and might be suspected as false-positive loci. In order to minimize false positive rates, we used the loci that were jointly identified by two methods at the same time.

### Spatial population genetic structure

Landscape genomics studies have focused mostly on the spatial population genetic structure of species [20, 21]. The influence of environmental variables on population genetic structure has been increasingly revealed. However, addressing spatial population genetic structure contributed by environmental variables is a serious challenge because of the complex reciprocal interactions of multifactors (i.e., gene flow, natural selection, and historical events) [22–24]. Our survey on SCoT data demonstrated significant hierarchical population genetic structure across all the studied populations*.* The two genetic groups, the East group (P1 to P5) and the West group (P6 to P15) are geographically separated. The spatial pattern is quite different from previous findings in Wang et al. [13]. This is mainly due to the difference in markers. The neutral cpDNA markers mainly reflect seed mediated gene flow and population dynamics. SCOT markers can simultaneously reflect seed and pollen mediated gene flow, as well as population dynamics and species adaptive evolution. Our results showing significant IBD pattern in *C. coggygria* indicated restricted gene flow. However, the restricted gene flow can only increase the degree of genetic differentiation among populations and cannot explain the spatial separation of the two genetic groups. Three hypotheses can be used to explain this large-scale intraspecific genetic disjunction. First, *C. coggygria* was compressed to two refugia (i.e., gene pools) during climate fluctuations in the past, and the current distribution pattern resulted from the redistribution of the two gene pools. Second, a geographical barrier existed between the two groups, and long-term blocking of gene flow by geographical barrier led to the genetic divergence of the two groups. Third and last, significant environmental differences occurred between the two regions, and these heterogeneous environmental conditions resulted in divergent selection and eventually led to the genetic divergence of the two groups. Previous phylogeographical study on *C. coggygria* suggested that this species survives in situ and occupies multiple localized glacial refugia rather than compressing to two refugia during the Pleistocene glaciations [13]. Therefore, the first hypothesis failed to explain the genetic divergence of *C. coggygria.* Assuming that *C. coggygria* agreed with the second hypothesis, significant genetic differentiation could be expected between the two groups with sufficiently strong geographic barrier. However, our AMOVA analysis based on all loci and STRUCTURE analysis with all loci except EAL (i.e., most of the neutral loci) both showed weak genetic differentiation between the two groups. Thus the second hypothesis was also not appropriate for *C. coggygria.* Considering that the third hypothesis was appropriate for *C. coggygria*, a shallow genetic divergence might occur between the two groups because of the interaction between natural selection and gene flow. This expectation is consistent with our detection results (*F*_CT_ = 0.053). To verify that the differences between the groups were caused by environmental factors, we compared the STRUCTURE analyses with all loci and all loci except EAL. When the EAL driven by environmental variables were excluded, the two genetic groups were also dissolved (Fig. 1a and b). Therefore, the genetic divergence of the two groups is due to the divergent selection driven by environmental factors. To further infer the relative contribution of environmental variables in driving population genetic structure, RDA was performed. The RDA results suggested that six environmental variables related to temperature and precipitation remarkably influenced the spatial population genetic structure. Among these environmental variables, Bio18 was the most important environmental factor in driving population genetic structure. Furthermore, our results showed that these environmental variables could significantly subdivide the populations into two groups (Fig. 4). In general, the third hypothesis holds true for *C. coggygria*.

### EAL driven by environmental factors

Previous phylogeographical studies have confirmed that the warm temperate vegetation adapts to climate change through migration or local adaptation [25]. Plants with long-term local adaptation often face the divergent selection driven by environmental variables, thereby leading to the adaptive evolution of species genome [4]. For some regions under extreme selection pressures, such as desert areas, species undergo convergent evolution at the genomic or phenotypic scale [26]. However, for most regions, such as the distribution area of *C. coggygria*, extreme selection pressures that drive species convergent evolution do not exist. In this case, species follows a variety of evolution directions. However, the environmental variables that significantly affect the genome and play a decisive role on the direction of species evolution are still unknown.

In this study, we selected *C. coggygria* as a model in addressing the abovementioned issues. We hypothesized that species ecological characters would drive adaptive evolution and produce large number of EAL. Here, the species ecological characters refer to the sensitivity, adaptability and resistance of species to environmental factors during local adaptation. Thus, examining the ecological characters of *C. coggygria* is urgently necessary. This species tolerates cold and drought, and avoids wetness. According to the adopted nine environmental variables, min temperature of coldest month (Bio6) and mean temperature of coldest quarter (Bio11) were associated with the ecological character of tolerating cold; precipitation of driest month (Bio14) was associated with tolerating drought, precipitation of warmest quarter (Bio18) were associated with avoiding wetness. In China’s warm-temperate zone, rainy and hot seasons overlap most of the time. Thus, precipitation of warmest quarter (Bio18) is tantamount to precipitation of wettest quarter (Bio16). The results of auto correlation analysis of environmental variables also confirmed this climate characteristic in this region. Therefore, we expected that the large numbers of EAL were associated with the environmental variables associated with the ecological characters, i.e. Bio6, Bio11, Bio14 and Bio18. As expected, the results of the Pearson’s correlation analysis and LFMM both showed that Bio14 and Bio18 were associated with the highest number of EAL. However, the environmental variables related to tolerating cold, Bio6 and Bio11, were not associated with high number of EAL. Our results suggested the environmental variables associated with the ecological characters of tolerating drought and avoiding wetness played more important roles in adaptive evolution in *C. coggygria*. Thus, most aspects of the characterized EAL of *C. coggygria* agreed with the hypothesis that ecological characters determine adaptation.

Whether the deduction that ecological character determines adaptation is universal must also be discussed. To date, landscape genomics studies in China’s warm-temperate zone are rare. Therefore, comparison with other species cannot help in confirming the universality of our results in this region. Thus, we reviewed some published works on landscape genomics in other regions in recent years. Prunier et al. [17] argued that the adaptive SNPs in *Picea mariana* are related to temperature and correspond to the nature of cold resistance of budset. De Kort et al. [16] mentioned that the detection of several temperature-dependent SNPs in *Alnus glutinosa* is related to its resistance to drought. Wang et al. [19] stated that the ecological characters of *Cephalotaxus oliveri* in response to temperature and precipitation sensitivity determine its adaptive evolution. Roschanski et al. [27] reported that the detected adaptive SNPs in *Abies alba* are associated with winter and drought and correspond to the ecological characters related to drought and cold tolerance. On the basis of this considerable evidence, we suggested that the ecological characters of species might be related to species adaptive evolution. Under high intensity of selection pressure, species usually loses ecological character and evolves toward extreme environments for survival. Without the strong selection pressure, species ecological character appears and evolution develops toward variety to ensure a better survival of species. This study provides a reference that ecological character, i.e. species sensitivity, adaptability and resistance to environmental factors, determines the adaptive evolution of species in regions lacking strong selection pressure.

## Conclusions

The differences in the intensity of selection pressure affect the direction of species evolution. In this study, we sampled *C. coggygria* from China’s warm-temperate zone, a region lacking strong selection pressure. Nine SCoT markers were used to investigate the adaptive genetic variation in *C. coggygria*. Our results showed that significant hierarchical population genetic structure of *C. coggygria* is due to the divergent selection driven by environmental factors. The ecological characters of *C. coggygria*, tolerating drought and avoiding wetness, determine its adaptive evolution. Therefore, species ecological character determines the adaptive evolution of species in regions lacking strong selection pressure.

## Methods

### Sample collection

A total of 156 individuals from fifteen natural populations of *C. coggygria* were collected from the entire distribution range in China (Fig. 3). Population samples included five to 12 individuals, and each sample was collected at least 10 meters apart. All individuals were collected when the population size was less than ten. Young, healthy leaves were collected and stored in silica gel at room temperature until DNA extraction and genotyping. The geographical coordinates for each sampled population are presented in Table 1.

### Molecular protocols

Genomic DNA was isolated from approximately 30 mg of dried leaves using Plant DNA Extraction Kit DP305 (Tiangen, Beijing, China) following the protocols of the manufacturer. DNA concentration was measured using Microcolume Spectrophotometer ND5000 (BioTeke, Beijing, China). After preliminary screening, nine SCoT primers (SCoT2, SCoT3, SCoT6, SCoT14, SCoT16, SCoT22, SCoT30, SCoT31, and SCoT33) from Collard and Mackill [14] were selected for polymerase chain reaction (PCR). SCoT2, SCoT16, and SCoT33 were 5′ fluorescent primers labeled with FAM; SCoT3, SCoT22, and SCoT30 were labeled with HEX; SCoT6, SCoT14, and SCoT31 were labeled with TAMRA. PCR was conducted in a 20 μL-reaction mixture consisting of 20 ng template DNA, 1 × reaction buffer (pH 8.3), 0.2 mM dNTPs, 0.3 μM primer, 1 unit of Taq polymerase, and DNA-free water. In an iCycler gene amplification system (Bioteke, Beijing, China), PCR was started with an initial denaturation at 94 °C for 5 min followed by 35 cycles at 94 °C for 40 s, primer-specific annealing temperature (50 °C for SCoT2; 52 °C for SCoT3, SCoT6, SCoT22, SCoT30, and SCoT31; 56 °C for SCoT16 and SCoT33; and 60 °C for SCoT14) for 45 s and 72 °C for 1 min, a final extension at 72 °C for 5 min, and termination by a final hold at 4 °C. PCR products were mixed with 10 μL of HiDi formamide and 0.1 μL of ROX1000 size standard (Applied Biosystems, Foster City, USA). These products were then separated on an ABI 3730 DNA Analyzer at BGI (Beijing, China).

### Data analysis

Electropherograms were viewed with GeneMarker 2.2.0 (SoftGenetics, State College, Pennsylvania, USA). To minimize scoring false alleles, peaks between 60 and 1000 bp and heights above 300 relative fluorescent units were scored as a presence (1) or absence (0) matrix for each sample. Subsequent statistical analyses were performed on the basis of this matrix.

AFLPSURV 1.0 [28] was used to calculate the genetic parameters for each population. The estimates included the number of polymorphic alleles (*N*_A_), percentage of polymorphic alleles (*PPA*), gene diversity of Nei (*H*_E_) [29], pairwise *F*st between populations, and gene frequencies per allele.

The analysis of hierarchical population structure was implemented using the Bayesian-based program STRUCTURE 2.3.4 [30]. For the analysis, a no-admixture model with independent allele frequencies was selected. *K* values were tested from 1 to 10, and 10 replicates were performed for each *K.* Burn-in periods of 1 × 10^5^ and 2 × 10^4^ Monte Carlo and Markov chains were specified. The choice of the optimal value of *K* was based on the method introduced by Evanno et al. [31], and this method was implemented in STRUCTURE HARVESTER [32]. Hierarchical and non-hierarchical AMOVA were calculated in ARLEQUIN 3.5.1.2 [33] in inferring the distribution of genetic differentiation at various levels. Mantel tests of isolation-by-distance (IBD) were performed in IBD 3.23 [34] in determining the relation of geographical distance (km) to genetic differentiation (*F*_ST_). RDA was conducted using CANOCO 4.5 [35] in disentangling the relative contribution of environmental variables in driving population genetic structure. In RDA, allele frequencies per population (Additional file 2) were used as the response variable and environmental variables (Additional file 3) were used as explanatory variables. Environmental data from 1950 to 2000 at 2.5 arcmin resolution were downloaded from the world climate database (http://www.diva-gis.org/climate). Additional data for each population were extracted using the DIVA-GIS 7.5.0 [36]. In China’s warm-temperate zone, rainy and hot are over the same period, cold and drought are over the same period. Thus, some environmental variables of temperature and precipitation might be significantly acossiated. To avoid overestimation of the contribution of environmental variables to population structure, the strong correlated environmental variables (*r* > 0.95) are excluded. Auto correlation analysis of environmental variables was performed using Pearson’s regression in SPSS 19 (SPSS Inc., Chicago, IL, USA).

Two approaches were used to identify the outlier loci. The first approach was based on the FDIST2 approach proposed by Beaumont and Nichols [37] and was implemented in the program Arlequin 3.5.1.2 [33]. The hierarchical island model in Arlequin was selected. The running parameters were set as follows: 100 simulated demes and 20,000 coalescent simulations. The loci outside the 95% confidence interval were regarded as outlier loci. To reduce the false discovery rate, loci with minor allele frequency < 5% were excluded. The second approach was based on the Bayesian approach and implemented in BayeScan 2.01 [38]. The running parameters were set as follows: sample size of 5000, thinning interval of 10, 20 pilot runs with a run length of 5000, and additional burn-in of 50,000 iterations. The loci with posterior probability >0.76 were regarded as outlier loci.

In further detecting the EAL potentially driven by environmental variations, two methods of environmental association analyses were performed. The first method is Pearson’s correlation analysis, which was implemented using SPSS 19 (SPSS Inc., Chicago, IL, USA). This regression analysis ignored the population structure, which might produce a relaxed result of EAL. Similar to RDA, allele frequencies per population were used as the response variable and environmental variables were utilized as explanatory variables. The loci with |r| > 0.50 and *P* < 0.05 were regarded as EAL. The second method is LFMM, which was implemented in LFMM 1.2 [39]. This Bayesian mixed model considered the population structure, thereby avoiding the bias caused by population history and isolation by distance and producing a robust result of EAL. The running parameters were set as follows: 10,000 sweeps, 1000 burn-in sweeps, and number of latent factors as suggested by STRUCTURE. The loci with |z| > 3 and *P* < 0.001 were regarded as EAL.

## Additional files

Additional file 1: The outlier loci identified by FDIST2 and BayeScan. (DOCX 27 kb)

Additional file 2; Gene frequencies per allele of 1131 alleles for each population. (DOCX 143 kb)

Additional file 3: Environmental variables for each location from the WorldClim database. (DOCX 15 kb)
