# Supplementary material for: Landscape genomics reveal that ecological character determines adaptation: a case study in smoke tree (Cotinus coggygria Scop.)
Source: BMC Evol Biol. 2017 Aug 23;17:202. doi: 10.1186/s12862-017-1055-3 (PMC5569454; doi:10.1186/s12862-017-1055-3)
Supplement: Supplementary file 1 — The outlier loci identified by FDIST2 and BayeScan. (DOCX 27 kb) [file 12862_2017_1055_MOESM1_ESM.docx]

**Additional file 1** The outlier loci identified by FDIST2 and BayeScan.

|  | FDIST2 | |  | BayeScan | |
| --- | --- | --- | --- | --- | --- |
| Locus code | Observe F_ST_ | *F*_ST_ P-value |  | Alpha coefficient | Posterior probability |
| 3-018 | -0.010 | 0.026 |  |  |  |
| 3-021 |  |  |  | 1.412 | 0.818 |
| 3-024 | -0.011 | 0.029 |  |  |  |
| 3-027 | -0.042 | 0.005 |  |  |  |
| 3-028 | 0.024 | 0.048 |  |  |  |
| 3-036 | -0.010 | 0.040 |  |  |  |
| 3-040 |  |  |  | 2.137 | 0.999 |
| 3-047 |  |  |  | 2.080 | 0.996 |
| 3-051 | 0.008 | 0.014 |  |  |  |
| 3-068 | 0.019 | 0.034 |  |  |  |
| 3-069 | -0.018 | 0.020 |  |  |  |
| 3-075 | 0.406 | 0.047 |  | 2.376 | 1.000 |
| 3-079 |  |  |  | 2.112 | 0.994 |
| 3-080 |  |  |  | 2.065 | 0.994 |
| 3-087 | -0.009 | 0.025 |  |  |  |
| 3-104 | 0.014 | 0.029 |  |  |  |
| 3-105 | 0.003 | 0.005 |  |  |  |
| 3-135 |  |  |  | 2.358 | 0.999 |
| 3-136 |  |  |  | 2.021 | 0.975 |
| 6-011 | -0.031 | 0.011 |  |  |  |
| 6-014 | 0.492 | 0.008 |  | 1.535 | 0.923 |
| 6-060 | -0.005 | 0.049 |  |  |  |
| 6-061 | -0.034 | 0.009 |  |  |  |
| 6-075 | 0.016 | 0.031 |  |  |  |
| 6-086 | 0.004 | 0.007 |  |  |  |
| 6-108 | -0.013 | 0.020 |  |  |  |
| 6-109 | -0.031 | 0.009 |  |  |  |
| 6-110 | 0.017 | 0.031 |  |  |  |
| 2-009 | -0.053 | 0.001 |  |  |  |
| 2-013 |  |  |  | 2.482 | 1.000 |
| 2-014 | 0.017 | 0.031 |  |  |  |
| 2-023 |  |  |  | 2.484 | 0.999 |
| 2-024 | 0.429 | 0.037 |  | 1.957 | 0.969 |
| 2-025 |  |  |  | 1.810 | 0.904 |
| 2-028 |  |  |  | 2.032 | 0.996 |
| 2-046 | 0.434 | 0.035 |  | 2.058 | 0.997 |
| 2-049 |  |  |  | 2.167 | 0.989 |
| 2-054 | 0.001 | 0.002 |  |  |  |
| 2-055 | 0.024 | 0.048 |  |  |  |
| 2-064 | 0.486 | 0.020 |  | 2.585 | 1.000 |
| 2-068 |  |  |  | 2.676 | 1.000 |
| 2-070 | 0.435 | 0.019 |  | 1.654 | 0.944 |
| 2-072 | 0.000 | 0.001 |  |  |  |
| 2-073 | 0.016 | 0.030 |  |  |  |
| 2-081 | -0.035 | 0.004 |  |  |  |
| 2-085 | 0.440 | 0.021 |  | 1.603 | 0.905 |
| 2-086 | 0.499 | 0.009 |  | 2.038 | 0.998 |
| 2-087 | 0.396 | 0.049 |  | 1.923 | 0.967 |
| 2-090 |  |  |  | 1.674 | 0.917 |
| 2-096 | 0.426 | 0.035 |  | 1.587 | 0.909 |
| 2-108 | 0.409 | 0.046 |  | 1.592 | 0.920 |
| 2-111 |  |  |  | 2.136 | 1.000 |
| 2-128 | 0.452 | 0.013 |  |  |  |
| 14-012 | 0.468 | 0.011 |  |  |  |
| 14-037 | 0.504 | 0.007 |  |  |  |
| 14-038 | 0.493 | 0.007 |  | 1.159 | 0.773 |
| 14-045 | 0.692 | 0.032 |  | 2.930 | 1.000 |
| 14-052 |  |  |  | 3.094 | 1.000 |
| 14-053 |  |  |  | 1.812 | 0.929 |
| 16-002 | 0.440 | 0.035 |  | 2.292 | 0.998 |
| 16-003 | 0.009 | 0.020 |  |  |  |
| 16-007 |  |  |  | 1.595 | 0.888 |
| 16-018 | 0.016 | 0.035 |  |  |  |
| 16-027 | -0.035 | 0.008 |  |  |  |
| 16-033 |  |  |  | 1.712 | 0.907 |
| 16-045 | -0.028 | 0.007 |  |  |  |
| 16-056 | -0.031 | 0.009 |  |  |  |
| 16-061 | -0.024 | 0.019 |  |  |  |
| 16-089 |  |  |  | 1.689 | 0.945 |
| 16-090 |  |  |  | 1.548 | 0.891 |
| 16-092 | 0.011 | 0.020 |  |  |  |
| 16-113 | -0.043 | 0.004 |  |  |  |
| 22-002 | 0.628 | 0.048 |  | 2.670 | 1.000 |
| 22-003 | 0.655 | 0.025 |  | 2.864 | 1.000 |
| 22-006 | 0.429 | 0.035 |  |  |  |
| 22-009 | 0.547 | 0.008 |  | 2.327 | 1.000 |
| 22-011 | 0.459 | 0.025 |  | 1.867 | 0.965 |
| 22-015 | 0.409 | 0.044 |  | 1.478 | 0.862 |
| 22-016 | 0.633 | 0.018 |  | 2.839 | 1.000 |
| 22-018 | 0.420 | 0.026 |  | 1.512 | 0.897 |
| 22-022 | 0.436 | 0.042 |  | 1.797 | 0.952 |
| 22-023 | 0.516 | 0.014 |  | 2.441 | 1.000 |
| 22-026 |  |  |  | 1.354 | 0.778 |
| 22-028 | -0.009 | 0.036 |  |  |  |
| 22-035 | -0.037 | 0.006 |  |  |  |
| 22-040 |  |  |  | 2.012 | 0.982 |
| 22-042 |  |  |  | 2.803 | 1.000 |
| 22-043 |  |  |  | 2.040 | 0.980 |
| 22-044 |  |  |  | 2.223 | 0.998 |
| 22-051 |  |  |  | 2.600 | 1.000 |
| 22-053 |  |  |  | 2.127 | 0.993 |
| 22-054 |  |  |  | 2.369 | 1.000 |
| 22-055 | -0.008 | 0.030 |  |  |  |
| 22-058 | -0.007 | 0.033 |  |  |  |
| 22-062 |  |  |  | 2.639 | 1.000 |
| 22-064 | -0.004 | 0.028 |  |  |  |
| 22-066 | -0.002 | 0.039 |  |  |  |
| 22-069 |  |  |  | 2.248 | 0.993 |
| 22-070 |  |  |  | 1.591 | 0.867 |
| 22-072 | 0.010 | 0.025 |  |  |  |
| 22-076 |  |  |  | 2.092 | 0.987 |
| 22-077 | 0.403 | 0.048 |  | 2.396 | 1.000 |
| 22-078 |  |  |  | 1.646 | 0.912 |
| 22-079 |  |  |  | 2.142 | 1.000 |
| 22-083 | 0.430 | 0.032 |  | 2.233 | 1.000 |
| 22-105 |  |  |  | 2.276 | 1.000 |
| 22-106 |  |  |  | 1.828 | 0.906 |
| 22-107 |  |  |  | 2.516 | 1.000 |
| 22-108 |  |  |  | 2.917 | 1.000 |
| 22-109 |  |  |  | 1.276 | 0.799 |
| 22-148 | 0.014 | 0.031 |  |  |  |
| 30-011 | 0.001 | 0.003 |  |  |  |
| 30-014 |  |  |  | 1.547 | 0.888 |
| 30-037 | 0.016 | 0.042 |  |  |  |
| 30-046 |  |  |  | 2.524 | 1.000 |
| 30-049 | 0.016 | 0.042 |  |  |  |
| 30-069 | -0.014 | 0.030 |  |  |  |
| 30-070 | 0.021 | 0.043 |  |  |  |
| 30-073 | -0.036 | 0.004 |  |  |  |
| 30-077 | -0.062 | 0.001 |  |  |  |
| 30-080 | -0.020 | 0.012 |  |  |  |
| 31-009 | 0.545 | 0.007 |  | 2.973 | 1.000 |
| 31-014 | 0.401 | 0.032 |  | 1.687 | 0.889 |
| 31-018 | 0.402 | 0.019 |  |  |  |
| 31-031 |  |  |  | 1.602 | 0.844 |
| 31-033 |  |  |  | 2.393 | 0.995 |
| 31-038 |  |  |  | 1.912 | 0.973 |
| 31-044 |  |  |  | 2.547 | 1.000 |
| 31-045 |  |  |  | 2.438 | 1.000 |
| 31-063 | 0.011 | 0.021 |  |  |  |
| 31-064 | -0.023 | 0.015 |  |  |  |
| 31-068 | 0.001 | 0.001 |  |  |  |
| 33-002 | 0.011 | 0.022 |  |  |  |
| 33-023 | -0.007 | 0.030 |  |  |  |
| 33-026 | -0.010 | 0.040 |  |  |  |
| 33-027 | -0.011 | 0.035 |  |  |  |
| 33-040 | 0.007 | 0.012 |  |  |  |
| 33-054 | -0.005 | 0.047 |  |  |  |
| 33-056 | 0.001 | 0.001 |  |  |  |
| 33-084 | -0.066 | 0.000 |  |  |  |
| 33-085 | -0.066 | 0.000 |  |  |  |
| 33-086 | -0.065 | 0.000 |  |  |  |
| 33-087 | -0.064 | 0.001 |  |  |  |
| 33-098 | -0.018 | 0.019 |  |  |  |
| 33-101 | 0.003 | 0.005 |  |  |  |
| 33-105 | -0.014 | 0.028 |  |  |  |
| 33-106 | -0.039 | 0.005 |  |  |  |
