# Supplementary material for: Landscape genomics reveal that ecological character determines adaptation: a case study in smoke tree (Cotinus coggygria Scop.)
Source: BMC Evol Biol. 2017 Aug 23;17:202. doi: 10.1186/s12862-017-1055-3 (PMC5569454; doi:10.1186/s12862-017-1055-3)
Supplement: Supplementary file 2 — Gene frequencies per allele of 1131 alleles for each population. (DOCX 143 kb) [file 12862_2017_1055_MOESM2_ESM.docx]

**Additional file 2** Gene frequencies per allele of 1131 alleles for each population.

| Locus code | Allele frequency | | | | | | | | | | | | | | |
| --- | --- | --- | --- | --- | --- | --- | --- | --- | --- | --- | --- | --- | --- | --- | --- |
|  | 1.HBWD | 2.HNSM | 3.SDBD | 4.HNJL | 5.SDYM | 6.SXLK | 7.HNLJ | 8.SXTB | 9.SXTT | 10.SXLJ | 11.SXWL | 12.HNYT | 13.SXHM | 14.SXTL | 15.HBTG |
| 3-001 | 0.00000 | 0.00000 | 0.00000 | 0.08333 | 0.08333 | 0.27273 | 0.08333 | 0.00000 | 0.00000 | 0.00000 | 0.00000 | 0.08333 | 0.00000 | 0.00000 | 0.16667 |
| 3-002 | 0.00000 | 0.00000 | 0.00000 | 0.08333 | 0.00000 | 0.09091 | 0.00000 | 0.00000 | 0.00000 | 0.00000 | 0.00000 | 0.00000 | 0.00000 | 0.00000 | 0.16667 |
| 3-003 | 0.00000 | 0.00000 | 0.00000 | 0.08333 | 0.00000 | 0.00000 | 0.00000 | 0.00000 | 0.00000 | 0.00000 | 0.00000 | 0.00000 | 0.00000 | 0.00000 | 0.00000 |
| 3-004 | 0.00000 | 0.00000 | 0.00000 | 0.08333 | 0.08333 | 0.09091 | 0.00000 | 0.00000 | 0.00000 | 0.00000 | 0.00000 | 0.00000 | 0.00000 | 0.10000 | 0.00000 |
| 3-005 | 0.00000 | 0.08333 | 0.00000 | 0.08333 | 0.00000 | 0.18182 | 0.00000 | 0.00000 | 0.00000 | 0.00000 | 0.00000 | 0.00000 | 0.00000 | 0.00000 | 0.16667 |
| 3-006 | 0.00000 | 0.00000 | 0.00000 | 0.00000 | 0.00000 | 0.09091 | 0.00000 | 0.00000 | 0.00000 | 0.00000 | 0.00000 | 0.00000 | 0.00000 | 0.00000 | 0.00000 |
| 3-007 | 0.00000 | 0.00000 | 0.00000 | 0.08333 | 0.00000 | 0.09091 | 0.08333 | 0.00000 | 0.00000 | 0.00000 | 0.00000 | 0.08333 | 0.00000 | 0.00000 | 0.00000 |
| 3-008 | 0.00000 | 0.00000 | 0.00000 | 0.08333 | 0.00000 | 0.09091 | 0.00000 | 0.00000 | 0.00000 | 0.00000 | 0.00000 | 0.00000 | 0.12500 | 0.20000 | 0.16667 |
| 3-009 | 0.00000 | 0.00000 | 0.00000 | 0.08333 | 0.08333 | 0.00000 | 0.08333 | 0.00000 | 0.00000 | 0.00000 | 0.00000 | 0.00000 | 0.00000 | 0.10000 | 0.00000 |
| 3-010 | 0.00000 | 0.00000 | 0.00000 | 0.08333 | 0.00000 | 0.00000 | 0.00000 | 0.00000 | 0.00000 | 0.00000 | 0.00000 | 0.00000 | 0.12500 | 0.00000 | 0.00000 |
| 3-011 | 0.00000 | 0.00000 | 0.00000 | 0.16667 | 0.00000 | 0.00000 | 0.00000 | 0.00000 | 0.00000 | 0.00000 | 0.00000 | 0.08333 | 0.00000 | 0.00000 | 0.16667 |
| 3-012 | 0.00000 | 0.08333 | 0.00000 | 0.00000 | 0.00000 | 0.09091 | 0.00000 | 0.00000 | 0.00000 | 0.00000 | 0.00000 | 0.00000 | 0.00000 | 0.10000 | 0.00000 |
| 3-013 | 0.00000 | 0.16667 | 0.00000 | 0.16667 | 0.00000 | 0.27273 | 0.00000 | 0.00000 | 0.00000 | 0.08333 | 0.22222 | 0.16667 | 0.12500 | 0.50000 | 0.00000 |
| 3-014 | 0.00000 | 0.08333 | 0.00000 | 0.16667 | 0.00000 | 0.09091 | 0.16667 | 0.00000 | 0.00000 | 0.00000 | 0.00000 | 0.00000 | 0.00000 | 0.00000 | 0.00000 |
| 3-015 | 0.00000 | 0.00000 | 0.00000 | 0.00000 | 0.00000 | 0.18182 | 0.08333 | 0.00000 | 0.00000 | 0.00000 | 0.00000 | 0.00000 | 0.00000 | 0.00000 | 0.00000 |
| 3-016 | 0.00000 | 0.08333 | 0.00000 | 0.00000 | 0.00000 | 0.18182 | 0.00000 | 0.00000 | 0.00000 | 0.08333 | 0.00000 | 0.08333 | 0.12500 | 0.40000 | 0.16667 |
| 3-017 | 0.00000 | 0.08333 | 0.00000 | 0.08333 | 0.00000 | 0.09091 | 0.08333 | 0.00000 | 0.00000 | 0.08333 | 0.00000 | 0.00000 | 0.12500 | 0.50000 | 0.00000 |
| 3-018 | 0.00000 | 0.08333 | 0.08333 | 0.16667 | 0.16667 | 0.18182 | 0.00000 | 0.25000 | 0.00000 | 0.00000 | 0.22222 | 0.16667 | 0.00000 | 0.20000 | 0.33333 |
| 3-019 | 0.00000 | 0.16667 | 0.16667 | 0.50000 | 0.00000 | 0.27273 | 0.08333 | 0.83333 | 0.09091 | 0.08333 | 0.22222 | 0.16667 | 0.12500 | 0.30000 | 0.16667 |
| 3-020 | 0.00000 | 0.66667 | 0.16667 | 0.33333 | 0.08333 | 0.27273 | 0.16667 | 0.08333 | 0.00000 | 0.50000 | 0.00000 | 0.08333 | 0.00000 | 0.20000 | 0.00000 |
| 3-021 | 0.00000 | 0.75000 | 0.16667 | 0.58333 | 0.08333 | 0.36364 | 0.16667 | 0.08333 | 0.00000 | 0.33333 | 0.00000 | 0.16667 | 0.00000 | 0.50000 | 0.00000 |
| 3-022 | 0.00000 | 0.00000 | 0.00000 | 0.08333 | 0.00000 | 0.00000 | 0.00000 | 0.00000 | 0.00000 | 0.00000 | 0.00000 | 0.08333 | 0.00000 | 0.00000 | 0.16667 |
| 3-023 | 0.00000 | 0.08333 | 0.00000 | 0.16667 | 0.00000 | 0.09091 | 0.08333 | 0.00000 | 0.00000 | 0.00000 | 0.00000 | 0.00000 | 0.12500 | 0.10000 | 0.00000 |
| 3-024 | 0.00000 | 0.25000 | 0.00000 | 0.08333 | 0.16667 | 0.18182 | 0.08333 | 0.00000 | 0.00000 | 0.08333 | 0.00000 | 0.08333 | 0.00000 | 0.10000 | 0.16667 |
| 3-025 | 0.00000 | 0.50000 | 0.25000 | 0.33333 | 0.16667 | 0.18182 | 0.58333 | 0.50000 | 0.00000 | 0.25000 | 0.22222 | 0.00000 | 0.00000 | 0.00000 | 0.00000 |
| 3-026 | 0.00000 | 0.25000 | 0.25000 | 0.41667 | 0.33333 | 0.45455 | 0.33333 | 0.41667 | 0.09091 | 0.41667 | 0.11111 | 0.41667 | 0.75000 | 0.50000 | 0.83333 |
| 3-027 | 0.00000 | 0.08333 | 0.08333 | 0.00000 | 0.00000 | 0.09091 | 0.08333 | 0.08333 | 0.00000 | 0.16667 | 0.00000 | 0.08333 | 0.00000 | 0.00000 | 0.00000 |
| 3-028 | 0.00000 | 0.33333 | 0.00000 | 0.00000 | 0.16667 | 0.09091 | 0.16667 | 0.00000 | 0.00000 | 0.08333 | 0.00000 | 0.16667 | 0.12500 | 0.10000 | 0.00000 |
| 3-029 | 0.00000 | 0.00000 | 0.00000 | 0.08333 | 0.08333 | 0.00000 | 0.08333 | 0.00000 | 0.00000 | 0.00000 | 0.00000 | 0.00000 | 0.00000 | 0.00000 | 0.00000 |
| 3-030 | 0.00000 | 0.75000 | 0.50000 | 0.66667 | 0.50000 | 0.45455 | 0.58333 | 0.16667 | 0.36364 | 0.25000 | 0.11111 | 0.50000 | 0.00000 | 0.40000 | 1.00000 |
| 3-031 | 0.00000 | 0.58333 | 0.16667 | 0.08333 | 0.08333 | 0.18182 | 0.08333 | 0.00000 | 0.09091 | 0.16667 | 0.00000 | 0.08333 | 0.00000 | 0.00000 | 0.00000 |
| 3-032 | 0.00000 | 0.00000 | 0.00000 | 0.16667 | 0.00000 | 0.18182 | 0.00000 | 0.00000 | 0.00000 | 0.00000 | 0.00000 | 0.08333 | 0.00000 | 0.30000 | 0.16667 |
| 3-033 | 0.00000 | 0.00000 | 0.00000 | 0.00000 | 0.16667 | 0.09091 | 0.00000 | 0.33333 | 0.18182 | 0.00000 | 0.33333 | 0.16667 | 0.50000 | 0.20000 | 0.16667 |
| 3-034 | 0.00000 | 0.00000 | 0.00000 | 0.00000 | 0.00000 | 0.00000 | 0.00000 | 0.00000 | 0.00000 | 0.08333 | 0.00000 | 0.00000 | 0.00000 | 0.00000 | 0.00000 |
| 3-035 | 0.00000 | 0.00000 | 0.08333 | 0.00000 | 0.16667 | 0.09091 | 0.00000 | 0.00000 | 0.00000 | 0.00000 | 0.00000 | 0.00000 | 0.00000 | 0.00000 | 0.00000 |
| 3-036 | 0.00000 | 0.08333 | 0.00000 | 0.16667 | 0.08333 | 0.18182 | 0.08333 | 0.00000 | 0.00000 | 0.00000 | 0.00000 | 0.08333 | 0.00000 | 0.00000 | 0.00000 |
| 3-037 | 0.00000 | 0.25000 | 0.00000 | 0.00000 | 0.16667 | 0.36364 | 0.08333 | 0.00000 | 0.00000 | 0.08333 | 0.00000 | 0.08333 | 0.12500 | 0.00000 | 0.00000 |
| 3-038 | 0.00000 | 0.08333 | 0.16667 | 0.00000 | 0.16667 | 0.09091 | 0.00000 | 0.00000 | 0.00000 | 0.00000 | 0.00000 | 0.00000 | 0.00000 | 0.00000 | 0.00000 |
| 3-039 | 0.00000 | 0.33333 | 0.00000 | 0.16667 | 0.16667 | 0.00000 | 0.16667 | 0.00000 | 0.00000 | 0.08333 | 0.00000 | 0.08333 | 0.00000 | 0.30000 | 0.00000 |
| 3-040 | 0.00000 | 0.75000 | 0.33333 | 0.16667 | 0.16667 | 0.54545 | 0.66667 | 0.00000 | 0.00000 | 0.58333 | 0.00000 | 0.16667 | 0.00000 | 0.40000 | 0.16667 |
| 3-041 | 0.00000 | 0.16667 | 0.00000 | 0.08333 | 0.00000 | 0.27273 | 0.08333 | 0.00000 | 0.00000 | 0.08333 | 0.00000 | 0.25000 | 0.00000 | 0.00000 | 0.16667 |
| 3-042 | 0.00000 | 0.33333 | 0.33333 | 0.25000 | 0.25000 | 0.36364 | 0.25000 | 0.00000 | 0.00000 | 0.00000 | 0.22222 | 0.25000 | 0.12500 | 0.70000 | 0.00000 |
| 3-043 | 0.00000 | 0.00000 | 0.00000 | 0.16667 | 0.00000 | 0.00000 | 0.00000 | 0.00000 | 0.00000 | 0.00000 | 0.00000 | 0.00000 | 0.00000 | 0.10000 | 0.16667 |
| 3-044 | 0.00000 | 0.00000 | 0.08333 | 0.08333 | 0.25000 | 0.09091 | 0.00000 | 0.00000 | 0.00000 | 0.00000 | 0.00000 | 0.08333 | 0.00000 | 0.00000 | 0.00000 |
| 3-045 | 0.00000 | 0.00000 | 0.08333 | 0.08333 | 0.00000 | 0.18182 | 0.00000 | 0.00000 | 0.00000 | 0.00000 | 0.00000 | 0.00000 | 0.00000 | 0.00000 | 0.00000 |
| 3-046 | 0.00000 | 0.00000 | 0.08333 | 0.25000 | 0.00000 | 0.00000 | 0.00000 | 0.00000 | 0.00000 | 0.00000 | 0.00000 | 0.00000 | 0.00000 | 0.00000 | 0.00000 |
| 3-047 | 0.20000 | 0.58333 | 0.58333 | 0.08333 | 0.33333 | 0.54545 | 0.25000 | 0.00000 | 0.09091 | 0.00000 | 0.00000 | 0.00000 | 0.00000 | 0.00000 | 0.00000 |
| 3-048 | 0.00000 | 0.08333 | 0.00000 | 0.08333 | 0.08333 | 0.09091 | 0.00000 | 0.00000 | 0.09091 | 0.00000 | 0.00000 | 0.00000 | 0.00000 | 0.00000 | 0.00000 |
| 3-049 | 0.00000 | 0.00000 | 0.08333 | 0.25000 | 0.00000 | 0.09091 | 0.00000 | 0.08333 | 0.00000 | 0.00000 | 0.00000 | 0.00000 | 0.00000 | 0.00000 | 0.00000 |
| 3-050 | 0.00000 | 0.00000 | 0.00000 | 0.33333 | 0.08333 | 0.18182 | 0.00000 | 0.00000 | 0.00000 | 0.00000 | 0.00000 | 0.00000 | 0.00000 | 0.00000 | 0.00000 |
| 3-051 | 0.00000 | 0.08333 | 0.08333 | 0.16667 | 0.25000 | 0.18182 | 0.00000 | 0.00000 | 0.00000 | 0.16667 | 0.00000 | 0.08333 | 0.00000 | 0.20000 | 0.00000 |
| 3-052 | 0.00000 | 0.16667 | 0.41667 | 0.08333 | 0.33333 | 0.18182 | 0.00000 | 0.00000 | 0.00000 | 0.00000 | 0.00000 | 0.00000 | 0.00000 | 0.10000 | 0.16667 |
| 3-053 | 0.00000 | 0.08333 | 0.33333 | 0.00000 | 0.33333 | 0.00000 | 0.00000 | 0.00000 | 0.00000 | 0.00000 | 0.00000 | 0.00000 | 0.00000 | 0.10000 | 0.00000 |
| 3-054 | 0.00000 | 0.00000 | 0.08333 | 0.00000 | 0.08333 | 0.18182 | 0.00000 | 0.00000 | 0.00000 | 0.00000 | 0.00000 | 0.00000 | 0.00000 | 0.00000 | 0.00000 |
| 3-055 | 0.00000 | 0.00000 | 0.00000 | 0.00000 | 0.16667 | 0.00000 | 0.00000 | 0.00000 | 0.00000 | 0.00000 | 0.00000 | 0.00000 | 0.00000 | 0.00000 | 0.00000 |
| 3-056 | 0.00000 | 0.00000 | 0.00000 | 0.00000 | 0.00000 | 0.09091 | 0.00000 | 0.08333 | 0.00000 | 0.00000 | 0.00000 | 0.00000 | 0.00000 | 0.00000 | 0.00000 |
| 3-057 | 0.00000 | 0.08333 | 0.00000 | 0.00000 | 0.08333 | 0.00000 | 0.00000 | 0.08333 | 0.00000 | 0.00000 | 0.00000 | 0.00000 | 0.00000 | 0.00000 | 0.00000 |
| 3-058 | 0.20000 | 0.58333 | 0.75000 | 0.66667 | 0.91667 | 0.54545 | 0.75000 | 1.00000 | 0.81818 | 0.83333 | 0.66667 | 0.66667 | 0.87500 | 0.50000 | 0.83333 |
| 3-059 | 0.20000 | 0.66667 | 0.83333 | 0.66667 | 0.91667 | 0.54545 | 0.66667 | 1.00000 | 0.81818 | 0.83333 | 0.66667 | 0.66667 | 0.87500 | 0.50000 | 0.83333 |
| 3-060 | 0.00000 | 0.08333 | 0.08333 | 0.16667 | 0.25000 | 0.09091 | 0.00000 | 0.00000 | 0.00000 | 0.00000 | 0.00000 | 0.00000 | 0.00000 | 0.00000 | 0.00000 |
| 3-061 | 0.00000 | 0.00000 | 0.08333 | 0.08333 | 0.08333 | 0.18182 | 0.00000 | 0.00000 | 0.00000 | 0.00000 | 0.00000 | 0.00000 | 0.00000 | 0.00000 | 0.00000 |
| 3-062 | 0.40000 | 0.33333 | 0.33333 | 0.16667 | 0.25000 | 0.63636 | 0.66667 | 0.00000 | 0.63636 | 0.00000 | 0.11111 | 0.41667 | 0.00000 | 0.40000 | 0.33333 |
| 3-063 | 0.20000 | 0.25000 | 0.08333 | 0.33333 | 0.25000 | 0.72727 | 0.25000 | 0.00000 | 0.36364 | 0.00000 | 0.11111 | 0.41667 | 0.00000 | 0.40000 | 0.16667 |
| 3-064 | 0.00000 | 0.00000 | 0.16667 | 0.08333 | 0.00000 | 0.09091 | 0.33333 | 0.00000 | 0.36364 | 0.00000 | 0.00000 | 0.00000 | 0.00000 | 0.10000 | 0.00000 |
| 3-065 | 0.80000 | 0.41667 | 0.66667 | 0.50000 | 0.75000 | 0.27273 | 0.66667 | 1.00000 | 0.63636 | 0.83333 | 0.66667 | 0.41667 | 0.87500 | 0.20000 | 0.66667 |
| 3-066 | 0.80000 | 0.50000 | 0.66667 | 0.58333 | 0.75000 | 0.27273 | 0.66667 | 1.00000 | 0.45455 | 0.83333 | 0.66667 | 0.41667 | 0.87500 | 0.10000 | 0.66667 |
| 3-067 | 0.00000 | 0.16667 | 0.00000 | 0.00000 | 0.08333 | 0.09091 | 0.00000 | 0.16667 | 0.00000 | 0.08333 | 0.00000 | 0.00000 | 0.00000 | 0.00000 | 0.00000 |
| 3-068 | 0.20000 | 0.41667 | 0.16667 | 0.33333 | 0.16667 | 0.36364 | 0.33333 | 0.33333 | 0.00000 | 0.41667 | 0.33333 | 0.08333 | 0.50000 | 0.10000 | 0.00000 |
| 3-069 | 0.00000 | 0.08333 | 0.08333 | 0.08333 | 0.16667 | 0.09091 | 0.00000 | 0.16667 | 0.00000 | 0.08333 | 0.22222 | 0.00000 | 0.25000 | 0.00000 | 0.00000 |
| 3-070 | 0.00000 | 0.16667 | 0.66667 | 0.25000 | 0.58333 | 0.45455 | 0.33333 | 0.08333 | 0.00000 | 0.08333 | 0.00000 | 0.08333 | 0.00000 | 0.30000 | 0.16667 |
| 3-071 | 0.00000 | 0.00000 | 0.00000 | 0.08333 | 0.00000 | 0.00000 | 0.00000 | 0.00000 | 0.00000 | 0.00000 | 0.00000 | 0.00000 | 0.00000 | 0.10000 | 0.00000 |
| 3-072 | 0.00000 | 0.00000 | 0.16667 | 0.00000 | 0.00000 | 0.00000 | 0.00000 | 0.00000 | 0.00000 | 0.00000 | 0.00000 | 0.00000 | 0.00000 | 0.00000 | 0.00000 |
| 3-073 | 0.20000 | 0.58333 | 0.33333 | 0.25000 | 0.16667 | 0.54545 | 0.58333 | 0.25000 | 0.27273 | 0.58333 | 0.00000 | 0.33333 | 0.12500 | 0.10000 | 0.00000 |
| 3-074 | 0.20000 | 0.33333 | 0.25000 | 0.08333 | 0.16667 | 0.45455 | 0.58333 | 0.25000 | 0.27273 | 0.58333 | 0.00000 | 0.33333 | 0.12500 | 0.10000 | 0.00000 |
| 3-075 | 0.00000 | 0.50000 | 0.50000 | 0.41667 | 0.66667 | 0.54545 | 0.00000 | 0.08333 | 0.00000 | 0.33333 | 0.00000 | 0.00000 | 0.00000 | 0.00000 | 0.00000 |
| 3-076 | 0.00000 | 0.25000 | 0.08333 | 0.08333 | 0.08333 | 0.27273 | 0.00000 | 0.00000 | 0.00000 | 0.00000 | 0.00000 | 0.00000 | 0.25000 | 0.10000 | 0.00000 |
| 3-077 | 0.00000 | 0.00000 | 0.16667 | 0.00000 | 0.08333 | 0.00000 | 0.00000 | 0.00000 | 0.00000 | 0.00000 | 0.00000 | 0.00000 | 0.00000 | 0.00000 | 0.00000 |
| 3-078 | 0.00000 | 0.00000 | 0.00000 | 0.08333 | 0.08333 | 0.00000 | 0.00000 | 0.00000 | 0.00000 | 0.00000 | 0.00000 | 0.00000 | 0.12500 | 0.20000 | 0.00000 |
| 3-079 | 0.20000 | 0.66667 | 0.75000 | 0.58333 | 0.66667 | 0.45455 | 0.33333 | 0.41667 | 0.00000 | 0.08333 | 0.00000 | 0.08333 | 0.00000 | 0.20000 | 0.16667 |
| 3-080 | 0.00000 | 0.25000 | 0.58333 | 0.41667 | 0.66667 | 0.45455 | 0.33333 | 0.41667 | 0.00000 | 0.00000 | 0.00000 | 0.08333 | 0.00000 | 0.00000 | 0.00000 |
| 3-081 | 0.00000 | 0.00000 | 0.00000 | 0.00000 | 0.08333 | 0.00000 | 0.00000 | 0.00000 | 0.00000 | 0.00000 | 0.00000 | 0.00000 | 0.00000 | 0.00000 | 0.00000 |
| 3-082 | 0.00000 | 0.00000 | 0.08333 | 0.16667 | 0.08333 | 0.00000 | 0.00000 | 0.00000 | 0.00000 | 0.00000 | 0.00000 | 0.00000 | 0.00000 | 0.00000 | 0.00000 |
| 3-083 | 0.00000 | 0.08333 | 0.08333 | 0.08333 | 0.25000 | 0.18182 | 0.00000 | 0.00000 | 0.00000 | 0.00000 | 0.00000 | 0.08333 | 0.00000 | 0.00000 | 0.00000 |
| 3-084 | 0.00000 | 0.08333 | 0.08333 | 0.08333 | 0.33333 | 0.18182 | 0.00000 | 0.00000 | 0.00000 | 0.00000 | 0.00000 | 0.00000 | 0.00000 | 0.00000 | 0.00000 |
| 3-085 | 0.00000 | 0.00000 | 0.00000 | 0.00000 | 0.08333 | 0.00000 | 0.00000 | 0.00000 | 0.00000 | 0.00000 | 0.00000 | 0.00000 | 0.00000 | 0.00000 | 0.00000 |
| 3-086 | 0.00000 | 0.00000 | 0.08333 | 0.08333 | 0.00000 | 0.00000 | 0.00000 | 0.00000 | 0.00000 | 0.00000 | 0.00000 | 0.00000 | 0.00000 | 0.00000 | 0.00000 |
| 3-087 | 0.20000 | 0.50000 | 0.50000 | 0.41667 | 0.50000 | 0.54545 | 0.58333 | 0.50000 | 0.09091 | 0.50000 | 0.22222 | 0.41667 | 0.75000 | 0.50000 | 0.50000 |
| 3-088 | 0.00000 | 0.25000 | 0.16667 | 0.41667 | 0.58333 | 0.54545 | 0.58333 | 0.91667 | 0.36364 | 0.25000 | 0.33333 | 0.33333 | 0.75000 | 0.40000 | 0.66667 |
| 3-089 | 0.00000 | 0.00000 | 0.16667 | 0.00000 | 0.00000 | 0.00000 | 0.00000 | 0.00000 | 0.00000 | 0.00000 | 0.00000 | 0.00000 | 0.00000 | 0.00000 | 0.00000 |
| 3-090 | 0.00000 | 0.00000 | 0.16667 | 0.00000 | 0.00000 | 0.00000 | 0.00000 | 0.00000 | 0.00000 | 0.00000 | 0.00000 | 0.00000 | 0.00000 | 0.00000 | 0.00000 |
| 3-091 | 0.00000 | 0.00000 | 0.00000 | 0.08333 | 0.00000 | 0.00000 | 0.00000 | 0.00000 | 0.00000 | 0.00000 | 0.00000 | 0.00000 | 0.00000 | 0.00000 | 0.00000 |
| 3-092 | 0.00000 | 0.08333 | 0.08333 | 0.16667 | 0.08333 | 0.09091 | 0.00000 | 0.00000 | 0.00000 | 0.00000 | 0.00000 | 0.00000 | 0.00000 | 0.00000 | 0.00000 |
| 3-093 | 0.00000 | 0.16667 | 0.25000 | 0.08333 | 0.00000 | 0.09091 | 0.00000 | 0.00000 | 0.00000 | 0.00000 | 0.00000 | 0.00000 | 0.00000 | 0.00000 | 0.00000 |
| 3-094 | 0.00000 | 0.16667 | 0.16667 | 0.00000 | 0.00000 | 0.00000 | 0.00000 | 0.00000 | 0.00000 | 0.08333 | 0.00000 | 0.00000 | 0.00000 | 0.00000 | 0.00000 |
| 3-095 | 0.00000 | 0.08333 | 0.25000 | 0.08333 | 0.00000 | 0.00000 | 0.00000 | 0.00000 | 0.00000 | 0.00000 | 0.00000 | 0.00000 | 0.00000 | 0.00000 | 0.00000 |
| 3-096 | 0.00000 | 0.00000 | 0.08333 | 0.00000 | 0.00000 | 0.00000 | 0.00000 | 0.00000 | 0.00000 | 0.00000 | 0.00000 | 0.00000 | 0.00000 | 0.00000 | 0.00000 |
| 3-097 | 0.00000 | 0.08333 | 0.00000 | 0.08333 | 0.08333 | 0.18182 | 0.00000 | 0.00000 | 0.00000 | 0.00000 | 0.00000 | 0.00000 | 0.00000 | 0.00000 | 0.00000 |
| 3-098 | 0.00000 | 0.00000 | 0.00000 | 0.08333 | 0.00000 | 0.00000 | 0.00000 | 0.00000 | 0.00000 | 0.00000 | 0.00000 | 0.00000 | 0.00000 | 0.00000 | 0.00000 |
| 3-099 | 0.00000 | 0.00000 | 0.08333 | 0.00000 | 0.00000 | 0.00000 | 0.00000 | 0.00000 | 0.00000 | 0.00000 | 0.00000 | 0.00000 | 0.00000 | 0.00000 | 0.00000 |
| 3-100 | 0.00000 | 0.00000 | 0.08333 | 0.00000 | 0.00000 | 0.00000 | 0.00000 | 0.00000 | 0.00000 | 0.00000 | 0.00000 | 0.00000 | 0.00000 | 0.00000 | 0.00000 |
| 3-101 | 0.00000 | 0.00000 | 0.00000 | 0.00000 | 0.08333 | 0.00000 | 0.00000 | 0.08333 | 0.00000 | 0.00000 | 0.00000 | 0.00000 | 0.00000 | 0.00000 | 0.00000 |
| 3-102 | 0.00000 | 0.00000 | 0.08333 | 0.00000 | 0.00000 | 0.00000 | 0.00000 | 0.00000 | 0.09091 | 0.00000 | 0.00000 | 0.00000 | 0.00000 | 0.00000 | 0.00000 |
| 3-103 | 0.00000 | 0.00000 | 0.08333 | 0.00000 | 0.08333 | 0.00000 | 0.00000 | 0.00000 | 0.00000 | 0.00000 | 0.00000 | 0.00000 | 0.00000 | 0.00000 | 0.00000 |
| 3-104 | 0.00000 | 0.16667 | 0.00000 | 0.16667 | 0.00000 | 0.27273 | 0.08333 | 0.00000 | 0.00000 | 0.00000 | 0.11111 | 0.00000 | 0.12500 | 0.10000 | 0.00000 |
| 3-105 | 0.00000 | 0.16667 | 0.08333 | 0.08333 | 0.00000 | 0.27273 | 0.08333 | 0.00000 | 0.00000 | 0.00000 | 0.11111 | 0.00000 | 0.12500 | 0.20000 | 0.00000 |
| 3-106 | 0.00000 | 0.00000 | 0.08333 | 0.00000 | 0.00000 | 0.00000 | 0.00000 | 0.00000 | 0.00000 | 0.00000 | 0.00000 | 0.00000 | 0.00000 | 0.00000 | 0.00000 |
| 3-107 | 0.00000 | 0.00000 | 0.08333 | 0.00000 | 0.00000 | 0.00000 | 0.00000 | 0.00000 | 0.00000 | 0.00000 | 0.00000 | 0.00000 | 0.00000 | 0.00000 | 0.00000 |
| 3-108 | 0.00000 | 0.00000 | 0.08333 | 0.08333 | 0.00000 | 0.09091 | 0.00000 | 0.00000 | 0.00000 | 0.00000 | 0.00000 | 0.00000 | 0.00000 | 0.00000 | 0.00000 |
| 3-109 | 0.00000 | 0.00000 | 0.00000 | 0.00000 | 0.08333 | 0.00000 | 0.00000 | 0.00000 | 0.00000 | 0.00000 | 0.00000 | 0.00000 | 0.00000 | 0.00000 | 0.00000 |
| 3-110 | 0.00000 | 0.00000 | 0.00000 | 0.00000 | 0.16667 | 0.00000 | 0.00000 | 0.00000 | 0.18182 | 0.00000 | 0.00000 | 0.00000 | 0.00000 | 0.10000 | 0.16667 |
| 3-111 | 0.00000 | 0.00000 | 0.00000 | 0.00000 | 0.08333 | 0.00000 | 0.00000 | 0.00000 | 0.18182 | 0.00000 | 0.00000 | 0.00000 | 0.12500 | 0.10000 | 0.16667 |
| 3-112 | 0.00000 | 0.00000 | 0.08333 | 0.00000 | 0.00000 | 0.00000 | 0.00000 | 0.33333 | 0.00000 | 0.00000 | 0.22222 | 0.00000 | 0.25000 | 0.00000 | 0.00000 |
| 3-113 | 0.00000 | 0.00000 | 0.08333 | 0.00000 | 0.00000 | 0.00000 | 0.00000 | 0.33333 | 0.00000 | 0.00000 | 0.22222 | 0.00000 | 0.62500 | 0.00000 | 0.00000 |
| 3-114 | 0.00000 | 0.00000 | 0.16667 | 0.00000 | 0.00000 | 0.18182 | 0.00000 | 0.00000 | 0.00000 | 0.00000 | 0.00000 | 0.00000 | 0.00000 | 0.00000 | 0.00000 |
| 3-115 | 0.00000 | 0.16667 | 0.00000 | 0.00000 | 0.08333 | 0.45455 | 0.16667 | 0.00000 | 0.00000 | 0.00000 | 0.00000 | 0.00000 | 0.00000 | 0.00000 | 0.00000 |
| 3-116 | 0.00000 | 0.00000 | 0.00000 | 0.00000 | 0.08333 | 0.36364 | 0.16667 | 0.08333 | 0.00000 | 0.00000 | 0.11111 | 0.00000 | 0.00000 | 0.00000 | 0.00000 |
| 3-117 | 0.00000 | 0.00000 | 0.00000 | 0.00000 | 0.00000 | 0.09091 | 0.00000 | 0.00000 | 0.00000 | 0.00000 | 0.00000 | 0.00000 | 0.00000 | 0.00000 | 0.00000 |
| 3-118 | 0.00000 | 0.00000 | 0.16667 | 0.00000 | 0.00000 | 0.00000 | 0.00000 | 0.00000 | 0.00000 | 0.00000 | 0.00000 | 0.00000 | 0.00000 | 0.00000 | 0.00000 |
| 3-119 | 0.00000 | 0.00000 | 0.00000 | 0.00000 | 0.08333 | 0.00000 | 0.00000 | 0.00000 | 0.00000 | 0.00000 | 0.00000 | 0.00000 | 0.00000 | 0.00000 | 0.00000 |
| 3-120 | 0.00000 | 0.00000 | 0.08333 | 0.00000 | 0.00000 | 0.00000 | 0.00000 | 0.00000 | 0.00000 | 0.00000 | 0.00000 | 0.00000 | 0.00000 | 0.00000 | 0.00000 |
| 3-121 | 0.00000 | 0.00000 | 0.08333 | 0.00000 | 0.00000 | 0.00000 | 0.00000 | 0.00000 | 0.00000 | 0.00000 | 0.00000 | 0.00000 | 0.00000 | 0.00000 | 0.00000 |
| 3-122 | 0.00000 | 0.00000 | 0.00000 | 0.00000 | 0.16667 | 0.00000 | 0.00000 | 0.08333 | 0.00000 | 0.00000 | 0.00000 | 0.00000 | 0.00000 | 0.00000 | 0.00000 |
| 3-123 | 0.00000 | 0.00000 | 0.00000 | 0.00000 | 0.08333 | 0.00000 | 0.00000 | 0.00000 | 0.00000 | 0.00000 | 0.00000 | 0.00000 | 0.00000 | 0.00000 | 0.00000 |
| 3-124 | 0.00000 | 0.08333 | 0.00000 | 0.00000 | 0.00000 | 0.00000 | 0.00000 | 0.00000 | 0.00000 | 0.00000 | 0.00000 | 0.00000 | 0.00000 | 0.00000 | 0.00000 |
| 3-125 | 0.00000 | 0.00000 | 0.00000 | 0.00000 | 0.00000 | 0.00000 | 0.00000 | 0.41667 | 0.00000 | 0.00000 | 0.00000 | 0.00000 | 0.25000 | 0.10000 | 0.33333 |
| 3-126 | 0.00000 | 0.00000 | 0.08333 | 0.00000 | 0.00000 | 0.00000 | 0.00000 | 0.00000 | 0.00000 | 0.00000 | 0.00000 | 0.00000 | 0.00000 | 0.00000 | 0.00000 |
| 3-127 | 0.00000 | 0.00000 | 0.00000 | 0.00000 | 0.08333 | 0.00000 | 0.00000 | 0.00000 | 0.00000 | 0.00000 | 0.00000 | 0.00000 | 0.00000 | 0.00000 | 0.00000 |
| 3-128 | 0.00000 | 0.00000 | 0.25000 | 0.00000 | 0.00000 | 0.09091 | 0.00000 | 0.00000 | 0.00000 | 0.00000 | 0.00000 | 0.00000 | 0.12500 | 0.10000 | 0.33333 |
| 3-129 | 0.00000 | 0.00000 | 0.33333 | 0.00000 | 0.08333 | 0.00000 | 0.00000 | 0.00000 | 0.00000 | 0.00000 | 0.00000 | 0.00000 | 0.00000 | 0.00000 | 0.00000 |
| 3-130 | 0.00000 | 0.00000 | 0.00000 | 0.08333 | 0.00000 | 0.00000 | 0.00000 | 0.00000 | 0.00000 | 0.00000 | 0.00000 | 0.00000 | 0.00000 | 0.00000 | 0.00000 |
| 3-131 | 0.00000 | 0.00000 | 0.16667 | 0.00000 | 0.00000 | 0.00000 | 0.00000 | 0.00000 | 0.00000 | 0.00000 | 0.00000 | 0.00000 | 0.00000 | 0.00000 | 0.00000 |
| 3-132 | 0.00000 | 0.00000 | 0.00000 | 0.00000 | 0.00000 | 0.00000 | 0.00000 | 0.00000 | 0.00000 | 0.00000 | 0.00000 | 0.00000 | 0.00000 | 0.10000 | 0.00000 |
| 3-133 | 0.00000 | 0.00000 | 0.08333 | 0.00000 | 0.08333 | 0.00000 | 0.00000 | 0.00000 | 0.00000 | 0.00000 | 0.00000 | 0.00000 | 0.00000 | 0.10000 | 0.00000 |
| 3-134 | 0.00000 | 0.00000 | 0.00000 | 0.00000 | 0.08333 | 0.00000 | 0.00000 | 0.00000 | 0.00000 | 0.00000 | 0.00000 | 0.00000 | 0.00000 | 0.00000 | 0.00000 |
| 3-135 | 0.40000 | 0.66667 | 0.58333 | 0.66667 | 0.75000 | 0.63636 | 0.75000 | 0.58333 | 0.00000 | 0.08333 | 0.00000 | 0.16667 | 0.12500 | 0.20000 | 0.00000 |
| 3-136 | 0.40000 | 0.66667 | 0.58333 | 0.66667 | 0.75000 | 0.54545 | 0.66667 | 0.41667 | 0.00000 | 0.25000 | 0.00000 | 0.08333 | 0.12500 | 0.30000 | 0.00000 |
| 3-137 | 0.00000 | 0.00000 | 0.16667 | 0.00000 | 0.00000 | 0.00000 | 0.00000 | 0.00000 | 0.00000 | 0.00000 | 0.00000 | 0.00000 | 0.00000 | 0.00000 | 0.00000 |
| 3-138 | 0.00000 | 0.00000 | 0.25000 | 0.00000 | 0.00000 | 0.00000 | 0.00000 | 0.00000 | 0.00000 | 0.00000 | 0.00000 | 0.00000 | 0.00000 | 0.00000 | 0.00000 |
| 3-139 | 0.00000 | 0.00000 | 0.00000 | 0.00000 | 0.00000 | 0.00000 | 0.00000 | 0.25000 | 0.00000 | 0.00000 | 0.11111 | 0.00000 | 0.00000 | 0.00000 | 0.00000 |
| 3-140 | 0.00000 | 0.00000 | 0.00000 | 0.00000 | 0.00000 | 0.00000 | 0.00000 | 0.25000 | 0.00000 | 0.00000 | 0.11111 | 0.00000 | 0.00000 | 0.00000 | 0.00000 |
| 3-141 | 0.00000 | 0.00000 | 0.00000 | 0.00000 | 0.00000 | 0.00000 | 0.00000 | 0.00000 | 0.00000 | 0.00000 | 0.00000 | 0.00000 | 0.00000 | 0.00000 | 0.00000 |
| 3-142 | 0.00000 | 0.00000 | 0.00000 | 0.00000 | 0.00000 | 0.00000 | 0.00000 | 0.00000 | 0.09091 | 0.00000 | 0.00000 | 0.00000 | 0.00000 | 0.00000 | 0.00000 |
| 3-143 | 0.00000 | 0.00000 | 0.00000 | 0.00000 | 0.00000 | 0.00000 | 0.00000 | 0.00000 | 0.00000 | 0.08333 | 0.00000 | 0.00000 | 0.00000 | 0.00000 | 0.00000 |
| 3-144 | 0.00000 | 0.00000 | 0.00000 | 0.00000 | 0.00000 | 0.00000 | 0.08333 | 0.08333 | 0.00000 | 0.00000 | 0.00000 | 0.00000 | 0.00000 | 0.00000 | 0.00000 |
| 6-001 | 0.00000 | 0.00000 | 0.00000 | 0.16667 | 0.09091 | 0.00000 | 0.00000 | 0.00000 | 0.00000 | 0.00000 | 0.00000 | 0.00000 | 0.00000 | 0.00000 | 0.20000 |
| 6-002 | 0.00000 | 0.00000 | 0.00000 | 0.00000 | 0.09091 | 0.09091 | 0.10000 | 0.00000 | 0.00000 | 0.00000 | 0.11111 | 0.08333 | 0.12500 | 0.00000 | 0.00000 |
| 6-003 | 0.00000 | 0.00000 | 0.00000 | 0.08333 | 0.00000 | 0.00000 | 0.00000 | 0.00000 | 0.00000 | 0.00000 | 0.00000 | 0.00000 | 0.00000 | 0.00000 | 0.00000 |
| 6-004 | 0.00000 | 0.00000 | 0.00000 | 0.08333 | 0.00000 | 0.00000 | 0.00000 | 0.00000 | 0.00000 | 0.00000 | 0.00000 | 0.00000 | 0.00000 | 0.00000 | 0.00000 |
| 6-005 | 0.00000 | 0.00000 | 0.00000 | 0.08333 | 0.00000 | 0.00000 | 0.00000 | 0.00000 | 0.00000 | 0.00000 | 0.00000 | 0.00000 | 0.00000 | 0.00000 | 0.00000 |
| 6-006 | 0.00000 | 0.00000 | 0.00000 | 0.00000 | 0.00000 | 0.09091 | 0.00000 | 0.00000 | 0.00000 | 0.00000 | 0.00000 | 0.00000 | 0.00000 | 0.10000 | 0.00000 |
| 6-007 | 0.00000 | 0.00000 | 0.00000 | 0.00000 | 0.00000 | 0.09091 | 0.00000 | 0.00000 | 0.00000 | 0.00000 | 0.00000 | 0.00000 | 0.00000 | 0.00000 | 0.00000 |
| 6-008 | 0.00000 | 0.08333 | 0.00000 | 0.00000 | 0.00000 | 0.09091 | 0.00000 | 0.00000 | 0.00000 | 0.08333 | 0.00000 | 0.00000 | 0.12500 | 0.20000 | 0.00000 |
| 6-009 | 0.00000 | 0.33333 | 0.00000 | 0.16667 | 0.00000 | 0.18182 | 0.00000 | 0.00000 | 0.00000 | 0.00000 | 0.00000 | 0.08333 | 0.12500 | 0.50000 | 0.00000 |
| 6-010 | 0.00000 | 0.08333 | 0.00000 | 0.16667 | 0.00000 | 0.27273 | 0.10000 | 0.00000 | 0.00000 | 0.08333 | 0.22222 | 0.16667 | 0.12500 | 0.50000 | 0.00000 |
| 6-011 | 0.00000 | 0.08333 | 0.00000 | 0.16667 | 0.00000 | 0.09091 | 0.10000 | 0.00000 | 0.00000 | 0.00000 | 0.11111 | 0.16667 | 0.00000 | 0.10000 | 0.00000 |
| 6-012 | 0.00000 | 0.00000 | 0.00000 | 0.08333 | 0.00000 | 0.00000 | 0.00000 | 0.00000 | 0.00000 | 0.00000 | 0.00000 | 0.00000 | 0.12500 | 0.00000 | 0.00000 |
| 6-013 | 0.00000 | 0.00000 | 0.00000 | 0.00000 | 0.00000 | 0.00000 | 0.10000 | 0.00000 | 0.00000 | 0.00000 | 0.00000 | 0.00000 | 0.00000 | 0.00000 | 0.20000 |
| 6-014 | 0.00000 | 0.66667 | 0.00000 | 0.25000 | 0.00000 | 0.00000 | 0.00000 | 0.00000 | 0.00000 | 0.00000 | 0.00000 | 0.00000 | 0.12500 | 0.00000 | 0.00000 |
| 6-015 | 0.00000 | 0.16667 | 0.00000 | 0.00000 | 0.09091 | 0.00000 | 0.00000 | 0.00000 | 0.00000 | 0.00000 | 0.00000 | 0.00000 | 0.00000 | 0.00000 | 0.00000 |
| 6-016 | 0.00000 | 0.00000 | 0.08333 | 0.16667 | 0.00000 | 0.00000 | 0.30000 | 0.00000 | 0.00000 | 0.08333 | 0.00000 | 0.00000 | 0.00000 | 0.30000 | 0.00000 |
| 6-017 | 0.00000 | 0.00000 | 0.00000 | 0.16667 | 0.00000 | 0.00000 | 0.00000 | 0.00000 | 0.00000 | 0.00000 | 0.00000 | 0.00000 | 0.00000 | 0.00000 | 0.00000 |
| 6-018 | 0.00000 | 0.00000 | 0.00000 | 0.08333 | 0.00000 | 0.00000 | 0.00000 | 0.00000 | 0.00000 | 0.00000 | 0.00000 | 0.00000 | 0.00000 | 0.00000 | 0.00000 |
| 6-019 | 0.00000 | 0.00000 | 0.08333 | 0.00000 | 0.09091 | 0.00000 | 0.00000 | 0.00000 | 0.00000 | 0.00000 | 0.00000 | 0.00000 | 0.00000 | 0.00000 | 0.00000 |
| 6-020 | 0.00000 | 0.00000 | 0.08333 | 0.00000 | 0.00000 | 0.00000 | 0.00000 | 0.00000 | 0.00000 | 0.00000 | 0.00000 | 0.00000 | 0.00000 | 0.00000 | 0.00000 |
| 6-021 | 0.00000 | 0.00000 | 0.08333 | 0.00000 | 0.00000 | 0.27273 | 0.00000 | 0.00000 | 0.00000 | 0.08333 | 0.00000 | 0.08333 | 0.12500 | 0.00000 | 0.00000 |
| 6-022 | 0.00000 | 0.08333 | 0.00000 | 0.16667 | 0.00000 | 0.00000 | 0.00000 | 0.08333 | 0.00000 | 0.33333 | 0.11111 | 0.25000 | 0.50000 | 0.00000 | 0.20000 |
| 6-023 | 0.00000 | 0.25000 | 0.00000 | 0.08333 | 0.00000 | 0.00000 | 0.00000 | 0.00000 | 0.00000 | 0.00000 | 0.00000 | 0.00000 | 0.00000 | 0.00000 | 0.00000 |
| 6-024 | 0.00000 | 0.00000 | 0.00000 | 0.00000 | 0.00000 | 0.00000 | 0.00000 | 0.00000 | 0.00000 | 0.00000 | 0.00000 | 0.00000 | 0.00000 | 0.10000 | 0.00000 |
| 6-025 | 0.00000 | 0.00000 | 0.00000 | 0.00000 | 0.00000 | 0.09091 | 0.00000 | 0.00000 | 0.00000 | 0.00000 | 0.00000 | 0.00000 | 0.00000 | 0.00000 | 0.00000 |
| 6-026 | 0.00000 | 0.00000 | 0.00000 | 0.08333 | 0.09091 | 0.00000 | 0.10000 | 0.00000 | 0.00000 | 0.00000 | 0.00000 | 0.00000 | 0.00000 | 0.00000 | 0.00000 |
| 6-027 | 0.00000 | 0.00000 | 0.00000 | 0.16667 | 0.00000 | 0.00000 | 0.00000 | 0.00000 | 0.00000 | 0.00000 | 0.00000 | 0.00000 | 0.12500 | 0.00000 | 0.00000 |
| 6-028 | 0.00000 | 0.00000 | 0.00000 | 0.00000 | 0.00000 | 0.09091 | 0.10000 | 0.00000 | 0.00000 | 0.00000 | 0.00000 | 0.16667 | 0.12500 | 0.00000 | 0.00000 |
| 6-029 | 0.00000 | 0.16667 | 0.00000 | 0.08333 | 0.00000 | 0.18182 | 0.10000 | 0.00000 | 0.00000 | 0.00000 | 0.00000 | 0.16667 | 0.12500 | 0.40000 | 0.00000 |
| 6-030 | 0.00000 | 0.00000 | 0.00000 | 0.00000 | 0.00000 | 0.00000 | 0.00000 | 0.00000 | 0.00000 | 0.00000 | 0.00000 | 0.00000 | 0.12500 | 0.00000 | 0.00000 |
| 6-031 | 0.00000 | 0.00000 | 0.00000 | 0.16667 | 0.00000 | 0.09091 | 0.00000 | 0.00000 | 0.00000 | 0.00000 | 0.00000 | 0.00000 | 0.00000 | 0.20000 | 0.00000 |
| 6-032 | 0.00000 | 0.00000 | 0.08333 | 0.08333 | 0.00000 | 0.18182 | 0.00000 | 0.16667 | 0.00000 | 0.00000 | 0.00000 | 0.00000 | 0.00000 | 0.00000 | 0.00000 |
| 6-033 | 0.00000 | 0.00000 | 0.00000 | 0.08333 | 0.18182 | 0.18182 | 0.00000 | 0.08333 | 0.00000 | 0.00000 | 0.00000 | 0.00000 | 0.12500 | 0.00000 | 0.00000 |
| 6-034 | 0.00000 | 0.00000 | 0.00000 | 0.00000 | 0.00000 | 0.09091 | 0.00000 | 0.00000 | 0.00000 | 0.00000 | 0.11111 | 0.00000 | 0.00000 | 0.00000 | 0.00000 |
| 6-035 | 0.00000 | 0.00000 | 0.00000 | 0.00000 | 0.09091 | 0.00000 | 0.00000 | 0.00000 | 0.00000 | 0.00000 | 0.11111 | 0.00000 | 0.00000 | 0.00000 | 0.00000 |
| 6-036 | 0.00000 | 0.25000 | 0.00000 | 0.08333 | 0.18182 | 0.45455 | 0.20000 | 0.00000 | 0.00000 | 0.00000 | 0.00000 | 0.16667 | 0.00000 | 0.30000 | 0.20000 |
| 6-037 | 1.00000 | 0.66667 | 0.50000 | 0.58333 | 0.72727 | 0.72727 | 0.60000 | 1.00000 | 1.00000 | 0.83333 | 0.77778 | 0.75000 | 0.87500 | 0.50000 | 0.80000 |
| 6-038 | 0.80000 | 0.66667 | 0.50000 | 0.66667 | 0.90909 | 0.72727 | 0.70000 | 1.00000 | 1.00000 | 0.83333 | 0.77778 | 0.75000 | 0.87500 | 0.50000 | 1.00000 |
| 6-039 | 0.00000 | 0.08333 | 0.16667 | 0.00000 | 0.00000 | 0.00000 | 0.00000 | 0.00000 | 0.00000 | 0.00000 | 0.00000 | 0.00000 | 0.00000 | 0.00000 | 0.00000 |
| 6-040 | 0.20000 | 0.58333 | 0.33333 | 0.41667 | 0.45455 | 0.18182 | 0.50000 | 0.08333 | 0.00000 | 0.50000 | 0.11111 | 0.00000 | 0.00000 | 0.10000 | 0.20000 |
| 6-041 | 0.00000 | 0.25000 | 0.08333 | 0.00000 | 0.09091 | 0.00000 | 0.00000 | 0.00000 | 0.00000 | 0.00000 | 0.11111 | 0.00000 | 0.00000 | 0.00000 | 0.00000 |
| 6-042 | 0.00000 | 0.00000 | 0.08333 | 0.00000 | 0.00000 | 0.00000 | 0.10000 | 0.00000 | 0.00000 | 0.00000 | 0.00000 | 0.00000 | 0.00000 | 0.00000 | 0.00000 |
| 6-043 | 0.00000 | 0.00000 | 0.00000 | 0.00000 | 0.00000 | 0.00000 | 0.00000 | 0.00000 | 0.00000 | 0.00000 | 0.00000 | 0.00000 | 0.12500 | 0.00000 | 0.00000 |
| 6-044 | 0.00000 | 0.08333 | 0.08333 | 0.00000 | 0.18182 | 0.00000 | 0.00000 | 0.00000 | 0.00000 | 0.00000 | 0.11111 | 0.00000 | 0.12500 | 0.00000 | 0.00000 |
| 6-045 | 0.00000 | 0.00000 | 0.16667 | 0.00000 | 0.00000 | 0.00000 | 0.00000 | 0.00000 | 0.00000 | 0.00000 | 0.11111 | 0.00000 | 0.00000 | 0.00000 | 0.00000 |
| 6-046 | 0.00000 | 0.00000 | 0.08333 | 0.00000 | 0.00000 | 0.00000 | 0.00000 | 0.00000 | 0.00000 | 0.08333 | 0.00000 | 0.00000 | 0.00000 | 0.00000 | 0.00000 |
| 6-047 | 0.00000 | 0.00000 | 0.08333 | 0.00000 | 0.00000 | 0.00000 | 0.00000 | 0.00000 | 0.00000 | 0.00000 | 0.00000 | 0.00000 | 0.00000 | 0.00000 | 0.00000 |
| 6-048 | 0.00000 | 0.00000 | 0.00000 | 0.08333 | 0.09091 | 0.00000 | 0.00000 | 0.00000 | 0.00000 | 0.00000 | 0.00000 | 0.00000 | 0.12500 | 0.10000 | 0.00000 |
| 6-049 | 0.00000 | 0.00000 | 0.08333 | 0.08333 | 0.09091 | 0.00000 | 0.00000 | 0.00000 | 0.00000 | 0.00000 | 0.00000 | 0.00000 | 0.00000 | 0.00000 | 0.00000 |
| 6-050 | 0.00000 | 0.00000 | 0.16667 | 0.00000 | 0.00000 | 0.00000 | 0.00000 | 0.00000 | 0.00000 | 0.00000 | 0.00000 | 0.00000 | 0.00000 | 0.00000 | 0.00000 |
| 6-051 | 0.00000 | 0.00000 | 0.08333 | 0.08333 | 0.00000 | 0.00000 | 0.00000 | 0.00000 | 0.00000 | 0.00000 | 0.00000 | 0.00000 | 0.12500 | 0.00000 | 0.00000 |
| 6-052 | 0.00000 | 0.00000 | 0.00000 | 0.08333 | 0.00000 | 0.00000 | 0.00000 | 0.00000 | 0.00000 | 0.00000 | 0.00000 | 0.00000 | 0.00000 | 0.00000 | 0.00000 |
| 6-053 | 0.00000 | 0.00000 | 0.08333 | 0.08333 | 0.00000 | 0.00000 | 0.00000 | 0.00000 | 0.00000 | 0.00000 | 0.00000 | 0.00000 | 0.00000 | 0.00000 | 0.00000 |
| 6-054 | 0.00000 | 0.00000 | 0.08333 | 0.00000 | 0.00000 | 0.00000 | 0.00000 | 0.00000 | 0.00000 | 0.00000 | 0.00000 | 0.00000 | 0.00000 | 0.00000 | 0.00000 |
| 6-055 | 0.00000 | 0.00000 | 0.00000 | 0.16667 | 0.09091 | 0.00000 | 0.00000 | 0.00000 | 0.00000 | 0.00000 | 0.00000 | 0.00000 | 0.00000 | 0.00000 | 0.00000 |
| 6-056 | 0.00000 | 0.00000 | 0.00000 | 0.08333 | 0.18182 | 0.00000 | 0.00000 | 0.00000 | 0.00000 | 0.00000 | 0.00000 | 0.08333 | 0.00000 | 0.00000 | 0.00000 |
| 6-057 | 0.00000 | 0.00000 | 0.00000 | 0.08333 | 0.00000 | 0.00000 | 0.00000 | 0.00000 | 0.00000 | 0.00000 | 0.00000 | 0.08333 | 0.00000 | 0.00000 | 0.00000 |
| 6-058 | 0.00000 | 0.00000 | 0.00000 | 0.00000 | 0.36364 | 0.09091 | 0.00000 | 0.08333 | 0.00000 | 0.00000 | 0.00000 | 0.08333 | 0.00000 | 0.00000 | 0.00000 |
| 6-059 | 0.00000 | 0.00000 | 0.08333 | 0.00000 | 0.27273 | 0.00000 | 0.00000 | 0.00000 | 0.00000 | 0.00000 | 0.00000 | 0.08333 | 0.00000 | 0.20000 | 0.00000 |
| 6-060 | 0.00000 | 0.08333 | 0.08333 | 0.00000 | 0.18182 | 0.09091 | 0.00000 | 0.00000 | 0.00000 | 0.08333 | 0.00000 | 0.00000 | 0.00000 | 0.20000 | 0.00000 |
| 6-061 | 0.00000 | 0.00000 | 0.08333 | 0.08333 | 0.18182 | 0.09091 | 0.00000 | 0.08333 | 0.00000 | 0.08333 | 0.11111 | 0.00000 | 0.00000 | 0.00000 | 0.00000 |
| 6-062 | 0.00000 | 0.00000 | 0.08333 | 0.00000 | 0.00000 | 0.00000 | 0.00000 | 0.00000 | 0.00000 | 0.00000 | 0.00000 | 0.00000 | 0.00000 | 0.00000 | 0.00000 |
| 6-063 | 0.00000 | 0.00000 | 0.00000 | 0.00000 | 0.09091 | 0.00000 | 0.10000 | 0.00000 | 0.00000 | 0.00000 | 0.00000 | 0.08333 | 0.00000 | 0.00000 | 0.00000 |
| 6-064 | 0.00000 | 0.08333 | 0.00000 | 0.08333 | 0.00000 | 0.00000 | 0.00000 | 0.00000 | 0.00000 | 0.00000 | 0.00000 | 0.00000 | 0.00000 | 0.10000 | 0.00000 |
| 6-065 | 0.00000 | 0.00000 | 0.08333 | 0.00000 | 0.00000 | 0.00000 | 0.00000 | 0.00000 | 0.00000 | 0.00000 | 0.00000 | 0.00000 | 0.00000 | 0.20000 | 0.00000 |
| 6-066 | 0.00000 | 0.00000 | 0.00000 | 0.08333 | 0.00000 | 0.00000 | 0.10000 | 0.00000 | 0.00000 | 0.00000 | 0.00000 | 0.00000 | 0.00000 | 0.00000 | 0.00000 |
| 6-067 | 0.00000 | 0.00000 | 0.00000 | 0.00000 | 0.09091 | 0.00000 | 0.00000 | 0.00000 | 0.00000 | 0.00000 | 0.00000 | 0.00000 | 0.00000 | 0.00000 | 0.00000 |
| 6-068 | 0.00000 | 0.00000 | 0.00000 | 0.00000 | 0.09091 | 0.00000 | 0.00000 | 0.00000 | 0.00000 | 0.00000 | 0.00000 | 0.00000 | 0.00000 | 0.00000 | 0.00000 |
| 6-069 | 0.00000 | 0.00000 | 0.00000 | 0.00000 | 0.09091 | 0.00000 | 0.00000 | 0.00000 | 0.00000 | 0.00000 | 0.00000 | 0.00000 | 0.00000 | 0.00000 | 0.00000 |
| 6-070 | 0.00000 | 0.00000 | 0.00000 | 0.00000 | 0.09091 | 0.00000 | 0.00000 | 0.00000 | 0.00000 | 0.00000 | 0.00000 | 0.00000 | 0.00000 | 0.00000 | 0.00000 |
| 6-071 | 0.00000 | 0.08333 | 0.08333 | 0.00000 | 0.00000 | 0.00000 | 0.00000 | 0.00000 | 0.00000 | 0.00000 | 0.00000 | 0.16667 | 0.00000 | 0.00000 | 0.00000 |
| 6-072 | 0.00000 | 0.41667 | 0.16667 | 0.08333 | 0.00000 | 0.18182 | 0.00000 | 0.08333 | 0.00000 | 0.33333 | 0.11111 | 0.08333 | 0.00000 | 0.10000 | 0.00000 |
| 6-073 | 0.00000 | 0.00000 | 0.00000 | 0.08333 | 0.00000 | 0.00000 | 0.00000 | 0.00000 | 0.00000 | 0.00000 | 0.00000 | 0.00000 | 0.00000 | 0.00000 | 0.00000 |
| 6-074 | 0.00000 | 0.00000 | 0.16667 | 0.16667 | 0.00000 | 0.00000 | 0.00000 | 0.00000 | 0.00000 | 0.00000 | 0.00000 | 0.00000 | 0.00000 | 0.00000 | 0.00000 |
| 6-075 | 0.00000 | 0.08333 | 0.08333 | 0.16667 | 0.27273 | 0.18182 | 0.10000 | 0.00000 | 0.00000 | 0.08333 | 0.00000 | 0.00000 | 0.00000 | 0.10000 | 0.00000 |
| 6-076 | 0.00000 | 0.00000 | 0.00000 | 0.00000 | 0.09091 | 0.00000 | 0.00000 | 0.00000 | 0.00000 | 0.00000 | 0.00000 | 0.00000 | 0.00000 | 0.00000 | 0.00000 |
| 6-077 | 0.00000 | 0.00000 | 0.16667 | 0.16667 | 0.36364 | 0.00000 | 0.00000 | 0.16667 | 0.00000 | 0.16667 | 0.00000 | 0.00000 | 0.00000 | 0.00000 | 0.00000 |
| 6-078 | 0.00000 | 0.00000 | 0.08333 | 0.00000 | 0.00000 | 0.00000 | 0.00000 | 0.00000 | 0.00000 | 0.00000 | 0.00000 | 0.00000 | 0.00000 | 0.00000 | 0.00000 |
| 6-079 | 0.00000 | 0.00000 | 0.08333 | 0.08333 | 0.00000 | 0.00000 | 0.00000 | 0.00000 | 0.00000 | 0.08333 | 0.00000 | 0.00000 | 0.00000 | 0.00000 | 0.00000 |
| 6-080 | 0.00000 | 0.00000 | 0.00000 | 0.00000 | 0.00000 | 0.00000 | 0.00000 | 0.00000 | 0.00000 | 0.08333 | 0.00000 | 0.00000 | 0.00000 | 0.00000 | 0.00000 |
| 6-081 | 0.00000 | 0.00000 | 0.16667 | 0.08333 | 0.00000 | 0.00000 | 0.00000 | 0.00000 | 0.00000 | 0.00000 | 0.00000 | 0.00000 | 0.00000 | 0.00000 | 0.00000 |
| 6-082 | 0.00000 | 0.00000 | 0.00000 | 0.00000 | 0.09091 | 0.00000 | 0.00000 | 0.00000 | 0.00000 | 0.00000 | 0.00000 | 0.00000 | 0.00000 | 0.00000 | 0.00000 |
| 6-083 | 0.00000 | 0.00000 | 0.00000 | 0.00000 | 0.09091 | 0.00000 | 0.00000 | 0.00000 | 0.00000 | 0.08333 | 0.00000 | 0.00000 | 0.00000 | 0.00000 | 0.00000 |
| 6-084 | 0.00000 | 0.00000 | 0.00000 | 0.08333 | 0.00000 | 0.18182 | 0.10000 | 0.00000 | 0.00000 | 0.00000 | 0.00000 | 0.00000 | 0.12500 | 0.00000 | 0.00000 |
| 6-085 | 0.00000 | 0.16667 | 0.00000 | 0.16667 | 0.00000 | 0.27273 | 0.10000 | 0.00000 | 0.00000 | 0.08333 | 0.22222 | 0.16667 | 0.12500 | 0.40000 | 0.00000 |
| 6-086 | 0.00000 | 0.16667 | 0.08333 | 0.16667 | 0.00000 | 0.18182 | 0.10000 | 0.00000 | 0.00000 | 0.16667 | 0.22222 | 0.16667 | 0.12500 | 0.40000 | 0.00000 |
| 6-087 | 0.00000 | 0.00000 | 0.00000 | 0.08333 | 0.09091 | 0.09091 | 0.00000 | 0.00000 | 0.00000 | 0.00000 | 0.11111 | 0.00000 | 0.00000 | 0.00000 | 0.00000 |
| 6-088 | 0.00000 | 0.00000 | 0.08333 | 0.00000 | 0.09091 | 0.00000 | 0.00000 | 0.00000 | 0.00000 | 0.00000 | 0.00000 | 0.00000 | 0.00000 | 0.00000 | 0.00000 |
| 6-089 | 0.00000 | 0.00000 | 0.00000 | 0.08333 | 0.00000 | 0.00000 | 0.00000 | 0.00000 | 0.00000 | 0.00000 | 0.00000 | 0.00000 | 0.00000 | 0.00000 | 0.00000 |
| 6-090 | 0.00000 | 0.00000 | 0.08333 | 0.00000 | 0.27273 | 0.00000 | 0.00000 | 0.00000 | 0.00000 | 0.00000 | 0.00000 | 0.08333 | 0.00000 | 0.00000 | 0.00000 |
| 6-091 | 0.00000 | 0.00000 | 0.00000 | 0.08333 | 0.00000 | 0.00000 | 0.00000 | 0.00000 | 0.00000 | 0.00000 | 0.00000 | 0.00000 | 0.00000 | 0.00000 | 0.00000 |
| 6-092 | 0.00000 | 0.00000 | 0.08333 | 0.00000 | 0.00000 | 0.00000 | 0.00000 | 0.00000 | 0.00000 | 0.00000 | 0.00000 | 0.00000 | 0.00000 | 0.00000 | 0.00000 |
| 6-093 | 0.00000 | 0.08333 | 0.00000 | 0.00000 | 0.00000 | 0.00000 | 0.00000 | 0.00000 | 0.00000 | 0.00000 | 0.00000 | 0.08333 | 0.00000 | 0.20000 | 0.60000 |
| 6-094 | 0.00000 | 0.08333 | 0.00000 | 0.00000 | 0.00000 | 0.00000 | 0.00000 | 0.00000 | 0.00000 | 0.00000 | 0.00000 | 0.16667 | 0.00000 | 0.20000 | 0.60000 |
| 6-095 | 0.00000 | 0.00000 | 0.00000 | 0.00000 | 0.18182 | 0.00000 | 0.00000 | 0.00000 | 0.00000 | 0.00000 | 0.00000 | 0.00000 | 0.00000 | 0.00000 | 0.00000 |
| 6-096 | 0.00000 | 0.00000 | 0.00000 | 0.00000 | 0.00000 | 0.00000 | 0.00000 | 0.00000 | 0.00000 | 0.00000 | 0.00000 | 0.08333 | 0.00000 | 0.00000 | 0.00000 |
| 6-097 | 0.00000 | 0.00000 | 0.00000 | 0.00000 | 0.00000 | 0.00000 | 0.00000 | 0.00000 | 0.00000 | 0.00000 | 0.00000 | 0.00000 | 0.00000 | 0.10000 | 0.00000 |
| 6-098 | 0.00000 | 0.00000 | 0.00000 | 0.00000 | 0.00000 | 0.00000 | 0.00000 | 0.00000 | 0.00000 | 0.00000 | 0.00000 | 0.00000 | 0.00000 | 0.20000 | 0.00000 |
| 6-099 | 0.00000 | 0.00000 | 0.00000 | 0.08333 | 0.00000 | 0.00000 | 0.00000 | 0.00000 | 0.00000 | 0.00000 | 0.00000 | 0.00000 | 0.00000 | 0.00000 | 0.00000 |
| 6-100 | 0.00000 | 0.00000 | 0.00000 | 0.00000 | 0.09091 | 0.00000 | 0.00000 | 0.00000 | 0.00000 | 0.00000 | 0.00000 | 0.00000 | 0.00000 | 0.00000 | 0.00000 |
| 6-101 | 0.00000 | 0.00000 | 0.00000 | 0.00000 | 0.09091 | 0.00000 | 0.00000 | 0.00000 | 0.00000 | 0.00000 | 0.00000 | 0.00000 | 0.00000 | 0.00000 | 0.00000 |
| 6-102 | 0.00000 | 0.00000 | 0.00000 | 0.00000 | 0.00000 | 0.00000 | 0.00000 | 0.00000 | 0.10000 | 0.00000 | 0.00000 | 0.00000 | 0.00000 | 0.00000 | 0.00000 |
| 6-103 | 0.00000 | 0.00000 | 0.00000 | 0.00000 | 0.09091 | 0.00000 | 0.00000 | 0.00000 | 0.00000 | 0.00000 | 0.00000 | 0.00000 | 0.00000 | 0.00000 | 0.00000 |
| 6-104 | 0.00000 | 0.00000 | 0.00000 | 0.00000 | 0.18182 | 0.00000 | 0.00000 | 0.00000 | 0.00000 | 0.00000 | 0.00000 | 0.00000 | 0.00000 | 0.00000 | 0.00000 |
| 6-105 | 0.00000 | 0.00000 | 0.00000 | 0.00000 | 0.18182 | 0.00000 | 0.00000 | 0.00000 | 0.00000 | 0.00000 | 0.00000 | 0.00000 | 0.00000 | 0.00000 | 0.00000 |
| 6-106 | 0.00000 | 0.00000 | 0.00000 | 0.00000 | 0.09091 | 0.00000 | 0.00000 | 0.00000 | 0.00000 | 0.00000 | 0.00000 | 0.08333 | 0.00000 | 0.00000 | 0.00000 |
| 6-107 | 0.00000 | 0.00000 | 0.00000 | 0.00000 | 0.00000 | 0.00000 | 0.00000 | 0.00000 | 0.00000 | 0.00000 | 0.00000 | 0.08333 | 0.12500 | 0.00000 | 0.00000 |
| 6-108 | 0.20000 | 0.25000 | 0.25000 | 0.16667 | 0.18182 | 0.27273 | 0.00000 | 0.08333 | 0.10000 | 0.16667 | 0.11111 | 0.08333 | 0.12500 | 0.00000 | 0.00000 |
| 6-109 | 0.40000 | 0.66667 | 0.66667 | 0.58333 | 0.72727 | 0.63636 | 0.60000 | 0.66667 | 0.60000 | 0.66667 | 0.55556 | 0.66667 | 0.62500 | 0.20000 | 0.40000 |
| 6-110 | 0.60000 | 0.83333 | 0.58333 | 0.58333 | 0.72727 | 0.63636 | 0.60000 | 0.83333 | 0.40000 | 0.83333 | 0.55556 | 0.75000 | 0.75000 | 0.40000 | 0.20000 |
| 6-111 | 0.20000 | 0.50000 | 0.08333 | 0.00000 | 0.09091 | 0.18182 | 0.10000 | 0.33333 | 0.10000 | 0.41667 | 0.22222 | 0.00000 | 0.12500 | 0.20000 | 0.20000 |
| 6-112 | 0.00000 | 0.00000 | 0.00000 | 0.00000 | 0.00000 | 0.00000 | 0.00000 | 0.00000 | 0.00000 | 0.00000 | 0.00000 | 0.00000 | 0.00000 | 0.10000 | 0.00000 |
| 6-113 | 0.00000 | 0.00000 | 0.00000 | 0.08333 | 0.00000 | 0.09091 | 0.00000 | 0.00000 | 0.00000 | 0.00000 | 0.00000 | 0.00000 | 0.00000 | 0.00000 | 0.00000 |
| 6-114 | 0.00000 | 0.00000 | 0.16667 | 0.00000 | 0.00000 | 0.00000 | 0.00000 | 0.00000 | 0.00000 | 0.00000 | 0.00000 | 0.00000 | 0.00000 | 0.00000 | 0.00000 |
| 6-115 | 0.00000 | 0.00000 | 0.16667 | 0.08333 | 0.00000 | 0.00000 | 0.00000 | 0.00000 | 0.00000 | 0.00000 | 0.00000 | 0.00000 | 0.00000 | 0.00000 | 0.00000 |
| 6-116 | 0.00000 | 0.00000 | 0.16667 | 0.00000 | 0.09091 | 0.09091 | 0.00000 | 0.00000 | 0.00000 | 0.00000 | 0.00000 | 0.00000 | 0.00000 | 0.00000 | 0.00000 |
| 2-001 | 0.00000 | 0.08333 | 0.00000 | 0.25000 | 0.08333 | 0.09091 | 0.00000 | 0.00000 | 0.00000 | 0.00000 | 0.11111 | 0.00000 | 0.00000 | 0.00000 | 0.00000 |
| 2-002 | 0.00000 | 0.08333 | 0.00000 | 0.08333 | 0.16667 | 0.00000 | 0.16667 | 0.00000 | 0.00000 | 0.00000 | 0.00000 | 0.00000 | 0.00000 | 0.00000 | 0.00000 |
| 2-003 | 0.00000 | 0.00000 | 0.00000 | 0.00000 | 0.00000 | 0.09091 | 0.08333 | 0.00000 | 0.00000 | 0.00000 | 0.00000 | 0.00000 | 0.00000 | 0.00000 | 0.00000 |
| 2-004 | 0.00000 | 0.00000 | 0.00000 | 0.08333 | 0.00000 | 0.00000 | 0.00000 | 0.00000 | 0.00000 | 0.00000 | 0.00000 | 0.00000 | 0.12500 | 0.00000 | 0.00000 |
| 2-005 | 0.00000 | 0.00000 | 0.00000 | 0.00000 | 0.00000 | 0.09091 | 0.00000 | 0.00000 | 0.00000 | 0.00000 | 0.00000 | 0.00000 | 0.00000 | 0.00000 | 0.00000 |
| 2-006 | 0.00000 | 0.00000 | 0.08333 | 0.08333 | 0.08333 | 0.00000 | 0.00000 | 0.00000 | 0.00000 | 0.00000 | 0.00000 | 0.00000 | 0.00000 | 0.00000 | 0.00000 |
| 2-007 | 0.00000 | 0.00000 | 0.00000 | 0.25000 | 0.08333 | 0.18182 | 0.00000 | 0.00000 | 0.00000 | 0.00000 | 0.00000 | 0.00000 | 0.00000 | 0.40000 | 0.00000 |
| 2-008 | 0.00000 | 0.00000 | 0.00000 | 0.16667 | 0.00000 | 0.09091 | 0.00000 | 0.00000 | 0.00000 | 0.00000 | 0.00000 | 0.00000 | 0.00000 | 0.00000 | 0.00000 |
| 2-009 | 0.00000 | 0.08333 | 0.00000 | 0.16667 | 0.08333 | 0.09091 | 0.08333 | 0.00000 | 0.00000 | 0.00000 | 0.00000 | 0.08333 | 0.12500 | 0.10000 | 0.00000 |
| 2-010 | 0.00000 | 0.00000 | 0.08333 | 0.16667 | 0.16667 | 0.00000 | 0.08333 | 0.00000 | 0.00000 | 0.00000 | 0.00000 | 0.00000 | 0.00000 | 0.00000 | 0.00000 |
| 2-011 | 0.00000 | 0.16667 | 0.00000 | 0.25000 | 0.00000 | 0.09091 | 0.00000 | 0.00000 | 0.00000 | 0.00000 | 0.00000 | 0.08333 | 0.00000 | 0.10000 | 0.00000 |
| 2-012 | 0.00000 | 0.08333 | 0.00000 | 0.00000 | 0.00000 | 0.09091 | 0.00000 | 0.00000 | 0.00000 | 0.00000 | 0.00000 | 0.00000 | 0.00000 | 0.20000 | 0.00000 |
| 2-013 | 1.00000 | 0.91667 | 0.66667 | 0.66667 | 0.91667 | 0.18182 | 0.25000 | 0.11111 | 0.50000 | 0.33333 | 0.77778 | 0.83333 | 1.00000 | 0.50000 | 1.00000 |
| 2-014 | 0.20000 | 0.16667 | 0.16667 | 0.16667 | 0.08333 | 0.27273 | 0.25000 | 0.00000 | 0.00000 | 0.00000 | 0.22222 | 0.16667 | 0.12500 | 0.50000 | 0.00000 |
| 2-015 | 0.00000 | 0.00000 | 0.08333 | 0.08333 | 0.08333 | 0.00000 | 0.16667 | 0.00000 | 0.00000 | 0.00000 | 0.00000 | 0.00000 | 0.00000 | 0.00000 | 0.00000 |
| 2-016 | 0.00000 | 0.00000 | 0.00000 | 0.00000 | 0.00000 | 0.00000 | 0.00000 | 0.00000 | 0.00000 | 0.00000 | 0.00000 | 0.08333 | 0.25000 | 0.00000 | 0.00000 |
| 2-017 | 0.00000 | 0.25000 | 0.00000 | 0.16667 | 0.08333 | 0.27273 | 0.08333 | 0.00000 | 0.00000 | 0.00000 | 0.22222 | 0.41667 | 0.12500 | 0.40000 | 0.00000 |
| 2-018 | 0.00000 | 0.08333 | 0.00000 | 0.08333 | 0.08333 | 0.00000 | 0.00000 | 0.00000 | 0.00000 | 0.00000 | 0.00000 | 0.25000 | 0.00000 | 0.00000 | 0.00000 |
| 2-019 | 0.00000 | 0.00000 | 0.00000 | 0.08333 | 0.00000 | 0.00000 | 0.00000 | 0.00000 | 0.00000 | 0.00000 | 0.00000 | 0.00000 | 0.00000 | 0.00000 | 0.00000 |
| 2-020 | 0.00000 | 0.08333 | 0.41667 | 0.41667 | 0.41667 | 0.00000 | 0.08333 | 0.00000 | 0.00000 | 0.00000 | 0.11111 | 0.41667 | 0.37500 | 0.10000 | 0.16667 |
| 2-021 | 0.00000 | 0.16667 | 0.08333 | 0.41667 | 0.16667 | 0.00000 | 0.16667 | 0.00000 | 0.16667 | 0.08333 | 0.11111 | 0.41667 | 0.12500 | 0.30000 | 0.33333 |
| 2-022 | 0.00000 | 0.08333 | 0.00000 | 0.00000 | 0.00000 | 0.00000 | 0.00000 | 0.00000 | 0.00000 | 0.00000 | 0.00000 | 0.00000 | 0.00000 | 0.00000 | 0.00000 |
| 2-023 | 0.00000 | 0.16667 | 0.25000 | 0.33333 | 0.08333 | 0.00000 | 0.16667 | 0.00000 | 0.16667 | 0.08333 | 0.77778 | 0.75000 | 1.00000 | 0.40000 | 0.66667 |
| 2-024 | 0.60000 | 0.25000 | 0.58333 | 0.58333 | 0.75000 | 0.00000 | 0.25000 | 0.11111 | 0.33333 | 0.08333 | 0.00000 | 0.08333 | 0.00000 | 0.00000 | 0.16667 |
| 2-025 | 0.60000 | 0.66667 | 0.66667 | 0.83333 | 0.83333 | 0.09091 | 0.25000 | 0.11111 | 0.50000 | 0.08333 | 0.66667 | 0.58333 | 0.87500 | 0.50000 | 0.50000 |
| 2-026 | 0.00000 | 0.08333 | 0.00000 | 0.16667 | 0.08333 | 0.00000 | 0.00000 | 0.00000 | 0.00000 | 0.08333 | 0.00000 | 0.00000 | 0.12500 | 0.10000 | 0.00000 |
| 2-027 | 0.00000 | 0.00000 | 0.00000 | 0.08333 | 0.00000 | 0.00000 | 0.00000 | 0.00000 | 0.00000 | 0.00000 | 0.00000 | 0.00000 | 0.00000 | 0.00000 | 0.00000 |
| 2-028 | 0.00000 | 0.66667 | 0.58333 | 0.41667 | 0.58333 | 0.00000 | 0.16667 | 0.11111 | 0.00000 | 0.00000 | 0.11111 | 0.41667 | 0.00000 | 0.00000 | 0.16667 |
| 2-029 | 0.00000 | 0.16667 | 0.08333 | 0.33333 | 0.33333 | 0.27273 | 0.08333 | 0.11111 | 0.00000 | 0.08333 | 0.22222 | 0.41667 | 0.25000 | 0.50000 | 0.00000 |
| 2-030 | 0.00000 | 0.08333 | 0.00000 | 0.08333 | 0.08333 | 0.00000 | 0.00000 | 0.00000 | 0.00000 | 0.00000 | 0.00000 | 0.00000 | 0.00000 | 0.00000 | 0.00000 |
| 2-031 | 0.00000 | 0.00000 | 0.00000 | 0.25000 | 0.00000 | 0.00000 | 0.00000 | 0.00000 | 0.00000 | 0.08333 | 0.00000 | 0.00000 | 0.00000 | 0.00000 | 0.00000 |
| 2-032 | 0.00000 | 0.00000 | 0.00000 | 0.00000 | 0.08333 | 0.00000 | 0.00000 | 0.00000 | 0.00000 | 0.00000 | 0.00000 | 0.00000 | 0.00000 | 0.00000 | 0.00000 |
| 2-033 | 0.40000 | 0.66667 | 0.66667 | 0.41667 | 0.58333 | 0.00000 | 0.25000 | 0.00000 | 0.16667 | 0.08333 | 0.22222 | 0.66667 | 0.50000 | 0.30000 | 0.50000 |
| 2-034 | 0.00000 | 0.00000 | 0.25000 | 0.25000 | 0.08333 | 0.00000 | 0.00000 | 0.00000 | 0.16667 | 0.08333 | 0.00000 | 0.00000 | 0.12500 | 0.00000 | 0.00000 |
| 2-035 | 0.00000 | 0.00000 | 0.41667 | 0.33333 | 0.41667 | 0.00000 | 0.00000 | 0.00000 | 0.00000 | 0.08333 | 0.00000 | 0.00000 | 0.00000 | 0.00000 | 0.00000 |
| 2-036 | 0.00000 | 0.00000 | 0.00000 | 0.08333 | 0.08333 | 0.00000 | 0.00000 | 0.00000 | 0.00000 | 0.00000 | 0.00000 | 0.00000 | 0.00000 | 0.00000 | 0.00000 |
| 2-037 | 0.00000 | 0.08333 | 0.00000 | 0.08333 | 0.00000 | 0.00000 | 0.00000 | 0.11111 | 0.00000 | 0.00000 | 0.00000 | 0.00000 | 0.00000 | 0.00000 | 0.00000 |
| 2-038 | 0.00000 | 0.16667 | 0.16667 | 0.33333 | 0.08333 | 0.27273 | 0.08333 | 0.00000 | 0.00000 | 0.16667 | 0.22222 | 0.25000 | 0.12500 | 0.60000 | 0.00000 |
| 2-039 | 0.00000 | 0.00000 | 0.08333 | 0.00000 | 0.08333 | 0.00000 | 0.00000 | 0.00000 | 0.00000 | 0.08333 | 0.00000 | 0.00000 | 0.00000 | 0.00000 | 0.00000 |
| 2-040 | 0.00000 | 0.08333 | 0.00000 | 0.25000 | 0.00000 | 0.09091 | 0.00000 | 0.00000 | 0.00000 | 0.00000 | 0.00000 | 0.00000 | 0.00000 | 0.00000 | 0.00000 |
| 2-041 | 0.00000 | 0.08333 | 0.08333 | 0.25000 | 0.08333 | 0.00000 | 0.00000 | 0.00000 | 0.00000 | 0.00000 | 0.00000 | 0.00000 | 0.00000 | 0.00000 | 0.00000 |
| 2-042 | 0.00000 | 0.08333 | 0.08333 | 0.25000 | 0.00000 | 0.00000 | 0.08333 | 0.00000 | 0.00000 | 0.00000 | 0.00000 | 0.08333 | 0.00000 | 0.20000 | 0.00000 |
| 2-043 | 0.00000 | 0.33333 | 0.16667 | 0.33333 | 0.00000 | 0.09091 | 0.08333 | 0.00000 | 0.00000 | 0.00000 | 0.00000 | 0.16667 | 0.00000 | 0.50000 | 0.33333 |
| 2-044 | 0.00000 | 0.41667 | 0.25000 | 0.50000 | 0.41667 | 0.09091 | 0.16667 | 0.00000 | 0.00000 | 0.08333 | 0.00000 | 0.08333 | 0.25000 | 0.10000 | 0.33333 |
| 2-045 | 0.00000 | 0.00000 | 0.16667 | 0.33333 | 0.25000 | 0.09091 | 0.08333 | 0.00000 | 0.00000 | 0.00000 | 0.00000 | 0.00000 | 0.00000 | 0.00000 | 0.00000 |
| 2-046 | 0.20000 | 0.50000 | 0.66667 | 0.41667 | 0.58333 | 0.18182 | 0.00000 | 0.00000 | 0.00000 | 0.00000 | 0.00000 | 0.25000 | 0.00000 | 0.10000 | 0.00000 |
| 2-047 | 0.00000 | 0.25000 | 0.08333 | 0.41667 | 0.33333 | 0.00000 | 0.00000 | 0.00000 | 0.00000 | 0.16667 | 0.00000 | 0.00000 | 0.00000 | 0.00000 | 0.00000 |
| 2-048 | 0.00000 | 0.00000 | 0.08333 | 0.16667 | 0.08333 | 0.09091 | 0.00000 | 0.00000 | 0.00000 | 0.00000 | 0.00000 | 0.08333 | 0.00000 | 0.00000 | 0.00000 |
| 2-049 | 1.00000 | 0.41667 | 0.75000 | 0.66667 | 0.83333 | 0.09091 | 0.08333 | 0.11111 | 0.16667 | 0.16667 | 0.00000 | 0.41667 | 0.37500 | 0.40000 | 0.66667 |
| 2-050 | 0.00000 | 0.33333 | 0.08333 | 0.00000 | 0.41667 | 0.09091 | 0.08333 | 0.00000 | 0.00000 | 0.08333 | 0.11111 | 0.00000 | 0.00000 | 0.00000 | 0.00000 |
| 2-051 | 0.20000 | 0.58333 | 0.66667 | 0.58333 | 0.75000 | 0.18182 | 0.41667 | 0.22222 | 0.50000 | 0.25000 | 0.55556 | 0.66667 | 0.75000 | 0.50000 | 0.66667 |
| 2-052 | 0.00000 | 0.16667 | 0.16667 | 0.33333 | 0.25000 | 0.09091 | 0.00000 | 0.00000 | 0.00000 | 0.00000 | 0.00000 | 0.00000 | 0.00000 | 0.10000 | 0.00000 |
| 2-053 | 0.00000 | 0.16667 | 0.08333 | 0.08333 | 0.08333 | 0.00000 | 0.00000 | 0.00000 | 0.00000 | 0.00000 | 0.00000 | 0.00000 | 0.00000 | 0.00000 | 0.00000 |
| 2-054 | 0.60000 | 0.58333 | 0.58333 | 0.58333 | 0.66667 | 0.54545 | 0.50000 | 0.33333 | 0.50000 | 0.50000 | 0.55556 | 0.83333 | 0.75000 | 0.30000 | 1.00000 |
| 2-055 | 0.00000 | 0.16667 | 0.16667 | 0.25000 | 0.08333 | 0.00000 | 0.00000 | 0.00000 | 0.33333 | 0.25000 | 0.11111 | 0.08333 | 0.12500 | 0.10000 | 0.50000 |
| 2-056 | 0.00000 | 0.00000 | 0.00000 | 0.16667 | 0.00000 | 0.00000 | 0.00000 | 0.00000 | 0.00000 | 0.08333 | 0.00000 | 0.00000 | 0.00000 | 0.10000 | 0.33333 |
| 2-057 | 0.00000 | 0.25000 | 0.33333 | 0.00000 | 0.08333 | 0.00000 | 0.00000 | 0.00000 | 0.00000 | 0.00000 | 0.00000 | 0.25000 | 0.12500 | 0.00000 | 0.16667 |
| 2-058 | 0.00000 | 0.16667 | 0.16667 | 0.16667 | 0.00000 | 0.00000 | 0.00000 | 0.00000 | 0.00000 | 0.00000 | 0.00000 | 0.00000 | 0.00000 | 0.00000 | 0.00000 |
| 2-059 | 0.00000 | 0.50000 | 0.25000 | 0.16667 | 0.50000 | 0.00000 | 0.00000 | 0.00000 | 0.00000 | 0.00000 | 0.00000 | 0.33333 | 0.00000 | 0.20000 | 0.33333 |
| 2-060 | 0.00000 | 0.41667 | 0.16667 | 0.16667 | 0.58333 | 0.00000 | 0.00000 | 0.00000 | 0.00000 | 0.00000 | 0.00000 | 0.33333 | 0.00000 | 0.10000 | 0.16667 |
| 2-061 | 0.00000 | 0.16667 | 0.00000 | 0.25000 | 0.08333 | 0.27273 | 0.08333 | 0.00000 | 0.00000 | 0.00000 | 0.00000 | 0.00000 | 0.00000 | 0.00000 | 0.00000 |
| 2-062 | 0.20000 | 0.66667 | 0.16667 | 0.41667 | 0.41667 | 0.27273 | 0.08333 | 0.00000 | 0.00000 | 0.00000 | 0.00000 | 0.08333 | 0.00000 | 0.10000 | 0.16667 |
| 2-063 | 0.00000 | 0.08333 | 0.16667 | 0.33333 | 0.00000 | 0.00000 | 0.00000 | 0.00000 | 0.00000 | 0.00000 | 0.00000 | 0.00000 | 0.00000 | 0.00000 | 0.00000 |
| 2-064 | 0.00000 | 0.66667 | 0.66667 | 0.50000 | 0.33333 | 0.00000 | 0.00000 | 0.11111 | 0.33333 | 0.00000 | 0.00000 | 0.00000 | 0.62500 | 0.00000 | 0.00000 |
| 2-065 | 0.20000 | 0.00000 | 0.16667 | 0.33333 | 0.33333 | 0.00000 | 0.00000 | 0.00000 | 0.00000 | 0.08333 | 0.22222 | 0.00000 | 0.00000 | 0.00000 | 0.16667 |
| 2-066 | 0.00000 | 0.00000 | 0.08333 | 0.08333 | 0.16667 | 0.00000 | 0.00000 | 0.00000 | 0.00000 | 0.00000 | 0.00000 | 0.00000 | 0.00000 | 0.00000 | 0.00000 |
| 2-067 | 0.20000 | 0.00000 | 0.33333 | 0.08333 | 0.16667 | 0.00000 | 0.00000 | 0.00000 | 0.00000 | 0.08333 | 0.00000 | 0.16667 | 0.00000 | 0.00000 | 0.00000 |
| 2-068 | 1.00000 | 0.83333 | 0.66667 | 0.66667 | 0.83333 | 0.09091 | 0.00000 | 0.11111 | 0.33333 | 0.16667 | 0.77778 | 0.75000 | 0.87500 | 0.50000 | 0.83333 |
| 2-069 | 0.00000 | 0.00000 | 0.25000 | 0.00000 | 0.08333 | 0.00000 | 0.00000 | 0.00000 | 0.00000 | 0.00000 | 0.00000 | 0.00000 | 0.00000 | 0.00000 | 0.00000 |
| 2-070 | 0.00000 | 0.00000 | 0.58333 | 0.25000 | 0.33333 | 0.00000 | 0.00000 | 0.00000 | 0.00000 | 0.00000 | 0.00000 | 0.00000 | 0.00000 | 0.00000 | 0.00000 |
| 2-071 | 0.00000 | 0.00000 | 0.50000 | 0.25000 | 0.08333 | 0.27273 | 0.00000 | 0.00000 | 0.00000 | 0.00000 | 0.00000 | 0.08333 | 0.12500 | 0.20000 | 0.00000 |
| 2-072 | 0.00000 | 0.08333 | 0.16667 | 0.16667 | 0.00000 | 0.09091 | 0.00000 | 0.00000 | 0.00000 | 0.00000 | 0.00000 | 0.00000 | 0.12500 | 0.10000 | 0.00000 |
| 2-073 | 0.00000 | 0.16667 | 0.16667 | 0.33333 | 0.00000 | 0.27273 | 0.08333 | 0.00000 | 0.00000 | 0.00000 | 0.22222 | 0.16667 | 0.12500 | 0.30000 | 0.00000 |
| 2-074 | 0.00000 | 0.25000 | 0.00000 | 0.16667 | 0.16667 | 0.09091 | 0.00000 | 0.00000 | 0.00000 | 0.00000 | 0.00000 | 0.00000 | 0.00000 | 0.00000 | 0.00000 |
| 2-075 | 0.00000 | 0.16667 | 0.50000 | 0.16667 | 0.16667 | 0.00000 | 0.08333 | 0.00000 | 0.00000 | 0.25000 | 0.00000 | 0.08333 | 0.00000 | 0.00000 | 0.00000 |
| 2-076 | 0.00000 | 0.00000 | 0.16667 | 0.25000 | 0.00000 | 0.18182 | 0.00000 | 0.00000 | 0.00000 | 0.00000 | 0.00000 | 0.00000 | 0.00000 | 0.00000 | 0.00000 |
| 2-077 | 0.00000 | 0.00000 | 0.41667 | 0.25000 | 0.08333 | 0.00000 | 0.00000 | 0.00000 | 0.00000 | 0.25000 | 0.00000 | 0.00000 | 0.00000 | 0.00000 | 0.00000 |
| 2-078 | 0.00000 | 0.00000 | 0.08333 | 0.16667 | 0.00000 | 0.00000 | 0.00000 | 0.00000 | 0.00000 | 0.00000 | 0.00000 | 0.00000 | 0.00000 | 0.00000 | 0.00000 |
| 2-079 | 0.20000 | 0.00000 | 0.00000 | 0.16667 | 0.08333 | 0.00000 | 0.00000 | 0.00000 | 0.00000 | 0.00000 | 0.00000 | 0.00000 | 0.00000 | 0.00000 | 0.00000 |
| 2-080 | 0.00000 | 0.00000 | 0.08333 | 0.16667 | 0.00000 | 0.00000 | 0.00000 | 0.00000 | 0.00000 | 0.00000 | 0.00000 | 0.00000 | 0.00000 | 0.00000 | 0.00000 |
| 2-081 | 1.00000 | 0.83333 | 0.75000 | 0.66667 | 0.83333 | 0.81818 | 0.91667 | 0.66667 | 0.83333 | 0.83333 | 0.77778 | 0.75000 | 0.87500 | 0.50000 | 0.83333 |
| 2-082 | 0.00000 | 0.00000 | 0.08333 | 0.08333 | 0.16667 | 0.09091 | 0.00000 | 0.00000 | 0.00000 | 0.00000 | 0.00000 | 0.00000 | 0.00000 | 0.00000 | 0.00000 |
| 2-083 | 0.00000 | 0.00000 | 0.08333 | 0.25000 | 0.08333 | 0.00000 | 0.00000 | 0.00000 | 0.00000 | 0.00000 | 0.00000 | 0.00000 | 0.00000 | 0.00000 | 0.00000 |
| 2-084 | 0.00000 | 0.50000 | 0.08333 | 0.08333 | 0.41667 | 0.00000 | 0.00000 | 0.00000 | 0.00000 | 0.08333 | 0.00000 | 0.00000 | 0.00000 | 0.10000 | 0.00000 |
| 2-085 | 0.00000 | 0.08333 | 0.25000 | 0.41667 | 0.58333 | 0.00000 | 0.00000 | 0.00000 | 0.00000 | 0.00000 | 0.00000 | 0.00000 | 0.00000 | 0.00000 | 0.00000 |
| 2-086 | 0.00000 | 0.41667 | 0.08333 | 0.50000 | 0.58333 | 0.00000 | 0.00000 | 0.00000 | 0.00000 | 0.00000 | 0.00000 | 0.00000 | 0.00000 | 0.00000 | 0.00000 |
| 2-087 | 0.00000 | 0.58333 | 0.58333 | 0.41667 | 0.41667 | 0.00000 | 0.00000 | 0.00000 | 0.00000 | 0.00000 | 0.00000 | 0.16667 | 0.00000 | 0.20000 | 0.33333 |
| 2-088 | 0.00000 | 0.00000 | 0.00000 | 0.16667 | 0.16667 | 0.00000 | 0.00000 | 0.00000 | 0.00000 | 0.00000 | 0.00000 | 0.00000 | 0.00000 | 0.00000 | 0.00000 |
| 2-089 | 0.00000 | 0.33333 | 0.08333 | 0.16667 | 0.25000 | 0.00000 | 0.00000 | 0.00000 | 0.00000 | 0.00000 | 0.00000 | 0.00000 | 0.00000 | 0.00000 | 0.00000 |
| 2-090 | 0.20000 | 0.33333 | 0.83333 | 0.41667 | 0.50000 | 0.18182 | 0.00000 | 0.00000 | 0.00000 | 0.08333 | 0.11111 | 0.08333 | 0.37500 | 0.10000 | 0.00000 |
| 2-091 | 0.00000 | 0.00000 | 0.33333 | 0.08333 | 0.08333 | 0.27273 | 0.00000 | 0.00000 | 0.00000 | 0.00000 | 0.00000 | 0.00000 | 0.00000 | 0.00000 | 0.00000 |
| 2-092 | 0.00000 | 0.00000 | 0.08333 | 0.00000 | 0.00000 | 0.00000 | 0.00000 | 0.00000 | 0.00000 | 0.00000 | 0.00000 | 0.00000 | 0.00000 | 0.00000 | 0.00000 |
| 2-093 | 0.00000 | 0.08333 | 0.08333 | 0.16667 | 0.08333 | 0.00000 | 0.00000 | 0.00000 | 0.00000 | 0.08333 | 0.00000 | 0.00000 | 0.12500 | 0.00000 | 0.00000 |
| 2-094 | 0.00000 | 0.00000 | 0.08333 | 0.25000 | 0.08333 | 0.00000 | 0.00000 | 0.00000 | 0.00000 | 0.00000 | 0.00000 | 0.00000 | 0.00000 | 0.00000 | 0.00000 |
| 2-095 | 0.00000 | 0.08333 | 0.08333 | 0.08333 | 0.08333 | 0.09091 | 0.00000 | 0.00000 | 0.00000 | 0.00000 | 0.00000 | 0.00000 | 0.00000 | 0.00000 | 0.00000 |
| 2-096 | 0.00000 | 0.25000 | 0.33333 | 0.16667 | 0.66667 | 0.00000 | 0.00000 | 0.00000 | 0.00000 | 0.00000 | 0.00000 | 0.00000 | 0.12500 | 0.00000 | 0.00000 |
| 2-097 | 0.00000 | 0.00000 | 0.25000 | 0.08333 | 0.33333 | 0.00000 | 0.00000 | 0.00000 | 0.00000 | 0.00000 | 0.00000 | 0.00000 | 0.12500 | 0.00000 | 0.00000 |
| 2-098 | 0.00000 | 0.00000 | 0.08333 | 0.00000 | 0.00000 | 0.00000 | 0.00000 | 0.00000 | 0.00000 | 0.00000 | 0.00000 | 0.00000 | 0.00000 | 0.00000 | 0.00000 |
| 2-099 | 0.00000 | 0.00000 | 0.16667 | 0.00000 | 0.08333 | 0.00000 | 0.00000 | 0.00000 | 0.00000 | 0.00000 | 0.00000 | 0.00000 | 0.00000 | 0.00000 | 0.00000 |
| 2-100 | 0.00000 | 0.00000 | 0.08333 | 0.00000 | 0.16667 | 0.00000 | 0.00000 | 0.00000 | 0.00000 | 0.00000 | 0.00000 | 0.00000 | 0.00000 | 0.00000 | 0.00000 |
| 2-101 | 0.00000 | 0.08333 | 0.16667 | 0.00000 | 0.08333 | 0.00000 | 0.00000 | 0.00000 | 0.00000 | 0.00000 | 0.00000 | 0.00000 | 0.00000 | 0.00000 | 0.00000 |
| 2-102 | 0.00000 | 0.08333 | 0.00000 | 0.00000 | 0.00000 | 0.00000 | 0.00000 | 0.00000 | 0.00000 | 0.00000 | 0.00000 | 0.00000 | 0.00000 | 0.00000 | 0.00000 |
| 2-103 | 0.00000 | 0.00000 | 0.00000 | 0.08333 | 0.00000 | 0.00000 | 0.00000 | 0.00000 | 0.00000 | 0.00000 | 0.00000 | 0.00000 | 0.00000 | 0.00000 | 0.00000 |
| 2-104 | 0.00000 | 0.00000 | 0.08333 | 0.00000 | 0.00000 | 0.00000 | 0.00000 | 0.00000 | 0.00000 | 0.00000 | 0.00000 | 0.00000 | 0.00000 | 0.00000 | 0.00000 |
| 2-105 | 0.00000 | 0.00000 | 0.00000 | 0.00000 | 0.08333 | 0.00000 | 0.00000 | 0.00000 | 0.00000 | 0.00000 | 0.00000 | 0.00000 | 0.00000 | 0.10000 | 0.00000 |
| 2-106 | 0.00000 | 0.00000 | 0.08333 | 0.08333 | 0.08333 | 0.00000 | 0.00000 | 0.00000 | 0.00000 | 0.00000 | 0.00000 | 0.00000 | 0.00000 | 0.00000 | 0.00000 |
| 2-107 | 0.00000 | 0.00000 | 0.08333 | 0.00000 | 0.00000 | 0.00000 | 0.00000 | 0.00000 | 0.00000 | 0.00000 | 0.00000 | 0.00000 | 0.00000 | 0.00000 | 0.00000 |
| 2-108 | 0.00000 | 0.58333 | 0.16667 | 0.33333 | 0.41667 | 0.09091 | 0.00000 | 0.00000 | 0.00000 | 0.00000 | 0.00000 | 0.00000 | 0.00000 | 0.00000 | 0.00000 |
| 2-109 | 0.00000 | 0.41667 | 0.16667 | 0.25000 | 0.33333 | 0.09091 | 0.00000 | 0.00000 | 0.00000 | 0.00000 | 0.00000 | 0.00000 | 0.00000 | 0.00000 | 0.00000 |
| 2-110 | 0.00000 | 0.25000 | 0.08333 | 0.50000 | 0.00000 | 0.36364 | 0.50000 | 0.00000 | 0.00000 | 0.08333 | 0.00000 | 0.08333 | 0.00000 | 0.10000 | 0.00000 |
| 2-111 | 0.00000 | 0.50000 | 0.41667 | 0.25000 | 0.66667 | 0.18182 | 0.58333 | 0.00000 | 0.00000 | 0.08333 | 0.00000 | 0.00000 | 0.00000 | 0.00000 | 0.00000 |
| 2-112 | 0.00000 | 0.16667 | 0.00000 | 0.16667 | 0.25000 | 0.18182 | 0.08333 | 0.00000 | 0.00000 | 0.08333 | 0.00000 | 0.00000 | 0.00000 | 0.00000 | 0.00000 |
| 2-113 | 0.00000 | 0.16667 | 0.00000 | 0.08333 | 0.08333 | 0.09091 | 0.00000 | 0.00000 | 0.00000 | 0.00000 | 0.00000 | 0.08333 | 0.00000 | 0.00000 | 0.00000 |
| 2-114 | 0.00000 | 0.08333 | 0.00000 | 0.08333 | 0.08333 | 0.00000 | 0.00000 | 0.00000 | 0.00000 | 0.00000 | 0.00000 | 0.00000 | 0.00000 | 0.00000 | 0.00000 |
| 2-115 | 0.00000 | 0.00000 | 0.00000 | 0.00000 | 0.08333 | 0.09091 | 0.00000 | 0.00000 | 0.00000 | 0.00000 | 0.00000 | 0.00000 | 0.00000 | 0.00000 | 0.00000 |
| 2-116 | 0.00000 | 0.00000 | 0.16667 | 0.00000 | 0.08333 | 0.00000 | 0.00000 | 0.00000 | 0.00000 | 0.00000 | 0.00000 | 0.00000 | 0.00000 | 0.00000 | 0.00000 |
| 2-117 | 0.00000 | 0.00000 | 0.08333 | 0.00000 | 0.00000 | 0.00000 | 0.00000 | 0.00000 | 0.00000 | 0.00000 | 0.00000 | 0.00000 | 0.00000 | 0.00000 | 0.00000 |
| 2-118 | 0.00000 | 0.00000 | 0.00000 | 0.00000 | 0.00000 | 0.00000 | 0.00000 | 0.00000 | 0.33333 | 0.00000 | 0.00000 | 0.00000 | 0.00000 | 0.00000 | 0.00000 |
| 2-119 | 0.00000 | 0.00000 | 0.16667 | 0.00000 | 0.08333 | 0.00000 | 0.00000 | 0.00000 | 0.00000 | 0.00000 | 0.00000 | 0.00000 | 0.00000 | 0.00000 | 0.00000 |
| 2-120 | 0.20000 | 0.50000 | 0.41667 | 0.25000 | 0.66667 | 0.27273 | 0.08333 | 0.77778 | 0.33333 | 0.58333 | 0.44444 | 0.25000 | 0.62500 | 0.50000 | 0.16667 |
| 2-121 | 0.20000 | 0.83333 | 0.75000 | 0.66667 | 0.66667 | 0.36364 | 0.66667 | 0.55556 | 0.16667 | 0.83333 | 0.22222 | 0.58333 | 0.62500 | 0.40000 | 0.50000 |
| 2-122 | 0.00000 | 0.16667 | 0.00000 | 0.33333 | 0.58333 | 0.09091 | 0.00000 | 0.00000 | 0.00000 | 0.00000 | 0.00000 | 0.00000 | 0.00000 | 0.10000 | 0.16667 |
| 2-123 | 0.00000 | 0.08333 | 0.08333 | 0.16667 | 0.41667 | 0.09091 | 0.00000 | 0.00000 | 0.00000 | 0.08333 | 0.00000 | 0.00000 | 0.00000 | 0.10000 | 0.16667 |
| 2-124 | 0.00000 | 0.25000 | 0.00000 | 0.16667 | 0.25000 | 0.00000 | 0.00000 | 0.00000 | 0.00000 | 0.00000 | 0.00000 | 0.00000 | 0.00000 | 0.00000 | 0.00000 |
| 2-125 | 0.00000 | 0.00000 | 0.00000 | 0.00000 | 0.00000 | 0.09091 | 0.00000 | 0.00000 | 0.00000 | 0.00000 | 0.00000 | 0.00000 | 0.00000 | 0.00000 | 0.00000 |
| 2-126 | 0.00000 | 0.00000 | 0.00000 | 0.00000 | 0.00000 | 0.00000 | 0.00000 | 0.00000 | 0.00000 | 0.08333 | 0.00000 | 0.00000 | 0.00000 | 0.00000 | 0.00000 |
| 2-127 | 0.00000 | 0.00000 | 0.00000 | 0.00000 | 0.00000 | 0.00000 | 0.00000 | 0.00000 | 0.00000 | 0.08333 | 0.00000 | 0.00000 | 0.00000 | 0.00000 | 0.00000 |
| 2-128 | 0.00000 | 0.16667 | 0.66667 | 0.00000 | 0.08333 | 0.09091 | 0.00000 | 0.00000 | 0.00000 | 0.00000 | 0.00000 | 0.00000 | 0.00000 | 0.00000 | 0.00000 |
| 2-129 | 0.00000 | 0.08333 | 0.41667 | 0.00000 | 0.00000 | 0.00000 | 0.00000 | 0.00000 | 0.00000 | 0.00000 | 0.00000 | 0.00000 | 0.00000 | 0.00000 | 0.00000 |
| 2-130 | 0.00000 | 0.00000 | 0.33333 | 0.00000 | 0.00000 | 0.09091 | 0.00000 | 0.00000 | 0.00000 | 0.00000 | 0.00000 | 0.00000 | 0.00000 | 0.00000 | 0.00000 |
| 2-131 | 0.00000 | 0.00000 | 0.25000 | 0.00000 | 0.00000 | 0.00000 | 0.00000 | 0.00000 | 0.00000 | 0.00000 | 0.00000 | 0.00000 | 0.00000 | 0.00000 | 0.00000 |
| 2-132 | 0.00000 | 0.00000 | 0.00000 | 0.00000 | 0.08333 | 0.09091 | 0.00000 | 0.00000 | 0.00000 | 0.08333 | 0.00000 | 0.00000 | 0.00000 | 0.00000 | 0.00000 |
| 2-133 | 0.00000 | 0.08333 | 0.16667 | 0.00000 | 0.08333 | 0.00000 | 0.00000 | 0.00000 | 0.00000 | 0.00000 | 0.00000 | 0.00000 | 0.00000 | 0.00000 | 0.00000 |
| 2-134 | 0.00000 | 0.00000 | 0.08333 | 0.00000 | 0.00000 | 0.00000 | 0.00000 | 0.00000 | 0.00000 | 0.00000 | 0.00000 | 0.00000 | 0.00000 | 0.00000 | 0.00000 |
| 2-135 | 0.00000 | 0.00000 | 0.08333 | 0.00000 | 0.00000 | 0.00000 | 0.00000 | 0.00000 | 0.00000 | 0.00000 | 0.00000 | 0.00000 | 0.00000 | 0.00000 | 0.00000 |
| 2-136 | 0.00000 | 0.00000 | 0.25000 | 0.00000 | 0.00000 | 0.00000 | 0.00000 | 0.00000 | 0.00000 | 0.00000 | 0.00000 | 0.00000 | 0.00000 | 0.00000 | 0.00000 |
| 2-137 | 0.00000 | 0.00000 | 0.25000 | 0.00000 | 0.08333 | 0.00000 | 0.00000 | 0.00000 | 0.00000 | 0.00000 | 0.00000 | 0.00000 | 0.00000 | 0.00000 | 0.00000 |
| 2-138 | 0.00000 | 0.00000 | 0.00000 | 0.00000 | 0.08333 | 0.00000 | 0.00000 | 0.00000 | 0.00000 | 0.00000 | 0.00000 | 0.00000 | 0.00000 | 0.00000 | 0.00000 |
| 2-139 | 0.00000 | 0.00000 | 0.08333 | 0.00000 | 0.08333 | 0.00000 | 0.00000 | 0.00000 | 0.00000 | 0.00000 | 0.00000 | 0.00000 | 0.00000 | 0.00000 | 0.00000 |
| 2-140 | 0.00000 | 0.00000 | 0.08333 | 0.00000 | 0.00000 | 0.00000 | 0.00000 | 0.00000 | 0.00000 | 0.00000 | 0.00000 | 0.00000 | 0.00000 | 0.00000 | 0.00000 |
| 2-141 | 0.00000 | 0.00000 | 0.00000 | 0.00000 | 0.08333 | 0.00000 | 0.00000 | 0.00000 | 0.00000 | 0.00000 | 0.00000 | 0.00000 | 0.00000 | 0.00000 | 0.00000 |
| 2-142 | 0.00000 | 0.00000 | 0.00000 | 0.00000 | 0.08333 | 0.00000 | 0.00000 | 0.00000 | 0.00000 | 0.00000 | 0.00000 | 0.00000 | 0.00000 | 0.00000 | 0.00000 |
| 2-143 | 0.00000 | 0.00000 | 0.08333 | 0.08333 | 0.08333 | 0.00000 | 0.08333 | 0.00000 | 0.00000 | 0.00000 | 0.00000 | 0.00000 | 0.00000 | 0.00000 | 0.00000 |
| 2-144 | 0.00000 | 0.08333 | 0.08333 | 0.08333 | 0.16667 | 0.00000 | 0.08333 | 0.00000 | 0.00000 | 0.00000 | 0.00000 | 0.00000 | 0.00000 | 0.00000 | 0.00000 |
| 2-145 | 0.00000 | 0.00000 | 0.00000 | 0.00000 | 0.16667 | 0.09091 | 0.08333 | 0.00000 | 0.00000 | 0.00000 | 0.00000 | 0.00000 | 0.00000 | 0.00000 | 0.00000 |
| 2-146 | 0.00000 | 0.00000 | 0.00000 | 0.08333 | 0.00000 | 0.09091 | 0.08333 | 0.00000 | 0.00000 | 0.00000 | 0.00000 | 0.00000 | 0.00000 | 0.00000 | 0.00000 |
| 2-147 | 0.00000 | 0.00000 | 0.00000 | 0.08333 | 0.08333 | 0.00000 | 0.00000 | 0.00000 | 0.00000 | 0.00000 | 0.00000 | 0.00000 | 0.00000 | 0.00000 | 0.00000 |
| 2-148 | 0.00000 | 0.00000 | 0.00000 | 0.00000 | 0.00000 | 0.00000 | 0.25000 | 0.00000 | 0.00000 | 0.00000 | 0.00000 | 0.00000 | 0.00000 | 0.00000 | 0.00000 |
| 2-149 | 0.00000 | 0.16667 | 0.08333 | 0.25000 | 0.16667 | 0.18182 | 0.41667 | 0.33333 | 0.00000 | 0.16667 | 0.00000 | 0.00000 | 0.00000 | 0.00000 | 0.00000 |
| 2-150 | 0.00000 | 0.25000 | 0.08333 | 0.08333 | 0.08333 | 0.00000 | 0.25000 | 0.00000 | 0.00000 | 0.08333 | 0.00000 | 0.00000 | 0.00000 | 0.00000 | 0.00000 |
| 2-151 | 0.00000 | 0.25000 | 0.08333 | 0.08333 | 0.33333 | 0.18182 | 0.33333 | 0.00000 | 0.00000 | 0.25000 | 0.00000 | 0.00000 | 0.00000 | 0.00000 | 0.00000 |
| 2-152 | 0.00000 | 0.33333 | 0.25000 | 0.33333 | 0.50000 | 0.09091 | 0.33333 | 0.00000 | 0.00000 | 0.16667 | 0.00000 | 0.00000 | 0.00000 | 0.00000 | 0.00000 |
| 2-153 | 0.00000 | 0.25000 | 0.08333 | 0.25000 | 0.25000 | 0.00000 | 0.00000 | 0.00000 | 0.00000 | 0.00000 | 0.00000 | 0.00000 | 0.00000 | 0.00000 | 0.00000 |
| 2-154 | 0.00000 | 0.00000 | 0.00000 | 0.00000 | 0.08333 | 0.00000 | 0.00000 | 0.00000 | 0.00000 | 0.00000 | 0.00000 | 0.00000 | 0.00000 | 0.00000 | 0.00000 |
| 2-155 | 0.00000 | 0.00000 | 0.08333 | 0.00000 | 0.00000 | 0.00000 | 0.00000 | 0.00000 | 0.00000 | 0.00000 | 0.00000 | 0.00000 | 0.00000 | 0.00000 | 0.00000 |
| 2-156 | 0.00000 | 0.00000 | 0.08333 | 0.00000 | 0.00000 | 0.00000 | 0.00000 | 0.00000 | 0.00000 | 0.00000 | 0.00000 | 0.00000 | 0.00000 | 0.00000 | 0.00000 |
| 2-157 | 0.00000 | 0.16667 | 0.08333 | 0.00000 | 0.00000 | 0.09091 | 0.08333 | 0.11111 | 0.00000 | 0.00000 | 0.00000 | 0.00000 | 0.00000 | 0.00000 | 0.00000 |
| 2-158 | 0.00000 | 0.00000 | 0.08333 | 0.00000 | 0.08333 | 0.00000 | 0.00000 | 0.00000 | 0.00000 | 0.00000 | 0.00000 | 0.00000 | 0.00000 | 0.00000 | 0.00000 |
| 2-159 | 0.00000 | 0.00000 | 0.25000 | 0.00000 | 0.08333 | 0.00000 | 0.00000 | 0.00000 | 0.00000 | 0.00000 | 0.00000 | 0.00000 | 0.00000 | 0.00000 | 0.00000 |
| 2-160 | 0.00000 | 0.00000 | 0.00000 | 0.00000 | 0.00000 | 0.00000 | 0.00000 | 0.00000 | 0.00000 | 0.16667 | 0.00000 | 0.00000 | 0.00000 | 0.00000 | 0.00000 |
| 2-161 | 0.00000 | 0.08333 | 0.16667 | 0.00000 | 0.00000 | 0.00000 | 0.00000 | 0.22222 | 0.00000 | 0.25000 | 0.00000 | 0.00000 | 0.00000 | 0.00000 | 0.00000 |
| 2-162 | 0.00000 | 0.00000 | 0.00000 | 0.00000 | 0.08333 | 0.00000 | 0.00000 | 0.00000 | 0.00000 | 0.00000 | 0.00000 | 0.00000 | 0.00000 | 0.00000 | 0.00000 |
| 2-163 | 0.00000 | 0.00000 | 0.16667 | 0.00000 | 0.00000 | 0.00000 | 0.00000 | 0.00000 | 0.00000 | 0.00000 | 0.00000 | 0.00000 | 0.00000 | 0.00000 | 0.00000 |
| 14-001 | 0.00000 | 0.00000 | 0.00000 | 0.00000 | 0.00000 | 0.18182 | 0.00000 | 0.00000 | 0.00000 | 0.00000 | 0.00000 | 0.00000 | 0.00000 | 0.00000 | 0.00000 |
| 14-002 | 0.00000 | 0.08333 | 0.00000 | 0.25000 | 0.00000 | 0.00000 | 0.00000 | 0.10000 | 0.00000 | 0.00000 | 0.00000 | 0.09091 | 0.00000 | 0.00000 | 0.16667 |
| 14-003 | 0.00000 | 0.08333 | 0.00000 | 0.16667 | 0.00000 | 0.00000 | 0.00000 | 0.00000 | 0.00000 | 0.00000 | 0.00000 | 0.00000 | 0.00000 | 0.00000 | 0.00000 |
| 14-004 | 0.00000 | 0.00000 | 0.00000 | 0.08333 | 0.00000 | 0.00000 | 0.00000 | 0.00000 | 0.00000 | 0.00000 | 0.00000 | 0.00000 | 0.00000 | 0.00000 | 0.00000 |
| 14-005 | 0.00000 | 0.00000 | 0.00000 | 0.00000 | 0.00000 | 0.00000 | 0.16667 | 0.00000 | 0.00000 | 0.00000 | 0.00000 | 0.00000 | 0.00000 | 0.00000 | 0.00000 |
| 14-006 | 0.00000 | 0.08333 | 0.00000 | 0.00000 | 0.00000 | 0.00000 | 0.00000 | 0.00000 | 0.00000 | 0.00000 | 0.00000 | 0.00000 | 0.00000 | 0.00000 | 0.00000 |
| 14-007 | 0.00000 | 0.00000 | 0.00000 | 0.00000 | 0.08333 | 0.00000 | 0.00000 | 0.00000 | 0.00000 | 0.00000 | 0.00000 | 0.00000 | 0.00000 | 0.00000 | 0.00000 |
| 14-008 | 0.00000 | 0.00000 | 0.08333 | 0.00000 | 0.00000 | 0.09091 | 0.08333 | 0.10000 | 0.00000 | 0.00000 | 0.00000 | 0.00000 | 0.00000 | 0.22222 | 0.00000 |
| 14-009 | 0.00000 | 0.00000 | 0.00000 | 0.00000 | 0.00000 | 0.00000 | 0.00000 | 0.10000 | 0.00000 | 0.00000 | 0.00000 | 0.00000 | 0.00000 | 0.11111 | 0.00000 |
| 14-010 | 0.00000 | 0.00000 | 0.08333 | 0.00000 | 0.00000 | 0.00000 | 0.00000 | 0.00000 | 0.00000 | 0.00000 | 0.00000 | 0.00000 | 0.00000 | 0.00000 | 0.00000 |
| 14-011 | 0.00000 | 0.00000 | 0.00000 | 0.00000 | 0.08333 | 0.00000 | 0.00000 | 0.00000 | 0.00000 | 0.00000 | 0.00000 | 0.00000 | 0.00000 | 0.00000 | 0.00000 |
| 14-012 | 0.00000 | 0.58333 | 0.00000 | 0.25000 | 0.00000 | 0.00000 | 0.00000 | 0.00000 | 0.00000 | 0.00000 | 0.00000 | 0.00000 | 0.00000 | 0.00000 | 0.00000 |
| 14-013 | 0.00000 | 0.58333 | 0.08333 | 0.25000 | 0.08333 | 0.00000 | 0.08333 | 0.00000 | 0.00000 | 0.00000 | 0.00000 | 0.00000 | 0.00000 | 0.00000 | 0.00000 |
| 14-014 | 0.25000 | 0.00000 | 0.00000 | 0.00000 | 0.08333 | 0.09091 | 0.08333 | 0.00000 | 0.00000 | 0.00000 | 0.00000 | 0.00000 | 0.00000 | 0.00000 | 0.00000 |
| 14-015 | 0.25000 | 0.33333 | 0.08333 | 0.16667 | 0.16667 | 0.00000 | 0.00000 | 0.00000 | 0.00000 | 0.00000 | 0.00000 | 0.00000 | 0.00000 | 0.00000 | 0.00000 |
| 14-016 | 0.00000 | 0.00000 | 0.00000 | 0.00000 | 0.16667 | 0.09091 | 0.00000 | 0.00000 | 0.00000 | 0.00000 | 0.00000 | 0.00000 | 0.00000 | 0.00000 | 0.00000 |
| 14-017 | 0.00000 | 0.00000 | 0.08333 | 0.00000 | 0.00000 | 0.00000 | 0.00000 | 0.00000 | 0.00000 | 0.00000 | 0.00000 | 0.00000 | 0.00000 | 0.00000 | 0.00000 |
| 14-018 | 0.25000 | 0.08333 | 0.00000 | 0.00000 | 0.00000 | 0.00000 | 0.00000 | 0.00000 | 0.00000 | 0.00000 | 0.00000 | 0.00000 | 0.00000 | 0.00000 | 0.00000 |
| 14-019 | 0.00000 | 0.33333 | 0.00000 | 0.00000 | 0.08333 | 0.00000 | 0.08333 | 0.00000 | 0.00000 | 0.00000 | 0.00000 | 0.00000 | 0.00000 | 0.00000 | 0.00000 |
| 14-020 | 0.00000 | 0.00000 | 0.00000 | 0.08333 | 0.00000 | 0.00000 | 0.08333 | 0.00000 | 0.00000 | 0.00000 | 0.00000 | 0.00000 | 0.00000 | 0.00000 | 0.00000 |
| 14-021 | 0.25000 | 0.66667 | 0.50000 | 0.33333 | 0.08333 | 0.72727 | 0.75000 | 0.60000 | 0.80000 | 0.58333 | 0.77778 | 0.81818 | 0.37500 | 0.55556 | 0.16667 |
| 14-022 | 0.00000 | 0.00000 | 0.08333 | 0.00000 | 0.00000 | 0.00000 | 0.00000 | 0.00000 | 0.00000 | 0.00000 | 0.00000 | 0.00000 | 0.00000 | 0.00000 | 0.00000 |
| 14-023 | 0.00000 | 0.16667 | 0.00000 | 0.00000 | 0.00000 | 0.00000 | 0.00000 | 0.00000 | 0.00000 | 0.00000 | 0.00000 | 0.00000 | 0.00000 | 0.00000 | 0.00000 |
| 14-024 | 0.00000 | 0.00000 | 0.00000 | 0.00000 | 0.00000 | 0.18182 | 0.00000 | 0.00000 | 0.00000 | 0.00000 | 0.00000 | 0.00000 | 0.00000 | 0.11111 | 0.00000 |
| 14-025 | 0.00000 | 0.00000 | 0.00000 | 0.00000 | 0.00000 | 0.18182 | 0.00000 | 0.00000 | 0.00000 | 0.00000 | 0.00000 | 0.00000 | 0.00000 | 0.11111 | 0.00000 |
| 14-026 | 0.25000 | 0.00000 | 0.00000 | 0.00000 | 0.00000 | 0.00000 | 0.00000 | 0.00000 | 0.00000 | 0.00000 | 0.00000 | 0.00000 | 0.00000 | 0.00000 | 0.00000 |
| 14-027 | 0.25000 | 0.00000 | 0.00000 | 0.00000 | 0.08333 | 0.36364 | 0.50000 | 0.40000 | 0.30000 | 0.33333 | 0.66667 | 0.45455 | 0.37500 | 0.22222 | 0.16667 |
| 14-028 | 0.00000 | 0.08333 | 0.00000 | 0.00000 | 0.08333 | 0.00000 | 0.00000 | 0.00000 | 0.00000 | 0.00000 | 0.00000 | 0.00000 | 0.00000 | 0.11111 | 0.00000 |
| 14-029 | 0.00000 | 0.16667 | 0.08333 | 0.16667 | 0.00000 | 0.00000 | 0.00000 | 0.10000 | 0.00000 | 0.00000 | 0.00000 | 0.00000 | 0.00000 | 0.11111 | 0.00000 |
| 14-030 | 0.00000 | 0.25000 | 0.08333 | 0.08333 | 0.00000 | 0.00000 | 0.00000 | 0.00000 | 0.00000 | 0.00000 | 0.00000 | 0.00000 | 0.00000 | 0.00000 | 0.00000 |
| 14-031 | 0.00000 | 0.08333 | 0.08333 | 0.08333 | 0.00000 | 0.00000 | 0.00000 | 0.00000 | 0.00000 | 0.00000 | 0.00000 | 0.00000 | 0.00000 | 0.00000 | 0.00000 |
| 14-032 | 0.00000 | 0.08333 | 0.08333 | 0.08333 | 0.00000 | 0.00000 | 0.08333 | 0.00000 | 0.00000 | 0.00000 | 0.00000 | 0.00000 | 0.00000 | 0.00000 | 0.00000 |
| 14-033 | 0.00000 | 0.00000 | 0.00000 | 0.00000 | 0.00000 | 0.00000 | 0.16667 | 0.00000 | 0.00000 | 0.00000 | 0.00000 | 0.00000 | 0.00000 | 0.00000 | 0.00000 |
| 14-034 | 0.00000 | 0.16667 | 0.16667 | 0.00000 | 0.00000 | 0.00000 | 0.00000 | 0.00000 | 0.00000 | 0.00000 | 0.00000 | 0.00000 | 0.00000 | 0.00000 | 0.00000 |
| 14-035 | 0.00000 | 0.16667 | 0.16667 | 0.08333 | 0.08333 | 0.00000 | 0.00000 | 0.00000 | 0.00000 | 0.00000 | 0.00000 | 0.00000 | 0.00000 | 0.00000 | 0.00000 |
| 14-036 | 0.00000 | 0.00000 | 0.00000 | 0.08333 | 0.00000 | 0.00000 | 0.00000 | 0.00000 | 0.00000 | 0.00000 | 0.00000 | 0.00000 | 0.00000 | 0.00000 | 0.00000 |
| 14-037 | 0.00000 | 0.66667 | 0.00000 | 0.00000 | 0.00000 | 0.00000 | 0.00000 | 0.10000 | 0.00000 | 0.00000 | 0.11111 | 0.00000 | 0.00000 | 0.00000 | 0.00000 |
| 14-038 | 0.00000 | 0.66667 | 0.16667 | 0.00000 | 0.00000 | 0.00000 | 0.00000 | 0.10000 | 0.00000 | 0.00000 | 0.00000 | 0.00000 | 0.00000 | 0.00000 | 0.00000 |
| 14-039 | 0.00000 | 0.08333 | 0.00000 | 0.00000 | 0.00000 | 0.00000 | 0.00000 | 0.00000 | 0.00000 | 0.00000 | 0.00000 | 0.00000 | 0.00000 | 0.00000 | 0.00000 |
| 14-040 | 0.00000 | 0.08333 | 0.08333 | 0.00000 | 0.00000 | 0.00000 | 0.00000 | 0.00000 | 0.00000 | 0.00000 | 0.00000 | 0.00000 | 0.00000 | 0.00000 | 0.00000 |
| 14-041 | 0.00000 | 0.00000 | 0.00000 | 0.08333 | 0.16667 | 0.18182 | 0.00000 | 0.00000 | 0.00000 | 0.41667 | 0.11111 | 0.00000 | 0.25000 | 0.00000 | 0.50000 |
| 14-042 | 0.00000 | 0.00000 | 0.00000 | 0.00000 | 0.00000 | 0.00000 | 0.00000 | 0.00000 | 0.00000 | 0.00000 | 0.00000 | 0.09091 | 0.00000 | 0.00000 | 0.00000 |
| 14-043 | 0.00000 | 0.00000 | 0.00000 | 0.00000 | 0.00000 | 0.00000 | 0.00000 | 0.00000 | 0.00000 | 0.00000 | 0.00000 | 0.18182 | 0.00000 | 0.00000 | 0.00000 |
| 14-044 | 0.00000 | 0.00000 | 0.00000 | 0.00000 | 0.00000 | 0.27273 | 0.00000 | 0.00000 | 0.00000 | 0.41667 | 0.11111 | 0.00000 | 0.25000 | 0.00000 | 0.50000 |
| 14-045 | 0.75000 | 0.75000 | 0.75000 | 0.66667 | 0.75000 | 0.09091 | 0.08333 | 0.10000 | 0.00000 | 0.00000 | 0.00000 | 0.00000 | 0.00000 | 0.11111 | 0.00000 |
| 14-046 | 0.00000 | 0.00000 | 0.00000 | 0.00000 | 0.00000 | 0.00000 | 0.00000 | 0.00000 | 0.00000 | 0.00000 | 0.00000 | 0.00000 | 0.00000 | 0.00000 | 0.00000 |
| 14-047 | 0.00000 | 0.16667 | 0.00000 | 0.41667 | 0.00000 | 0.00000 | 0.08333 | 0.10000 | 0.00000 | 0.00000 | 0.00000 | 0.00000 | 0.00000 | 0.00000 | 0.00000 |
| 14-048 | 0.00000 | 0.00000 | 0.00000 | 0.08333 | 0.00000 | 0.00000 | 0.08333 | 0.00000 | 0.00000 | 0.00000 | 0.00000 | 0.00000 | 0.00000 | 0.00000 | 0.00000 |
| 14-049 | 0.00000 | 0.00000 | 0.00000 | 0.00000 | 0.08333 | 0.00000 | 0.08333 | 0.00000 | 0.00000 | 0.00000 | 0.00000 | 0.00000 | 0.00000 | 0.00000 | 0.00000 |
| 14-050 | 0.00000 | 0.00000 | 0.00000 | 0.00000 | 0.00000 | 0.00000 | 0.08333 | 0.00000 | 0.00000 | 0.00000 | 0.00000 | 0.00000 | 0.00000 | 0.00000 | 0.00000 |
| 14-051 | 0.00000 | 0.00000 | 0.00000 | 0.00000 | 0.00000 | 0.18182 | 0.00000 | 0.00000 | 0.00000 | 0.00000 | 0.00000 | 0.00000 | 0.00000 | 0.11111 | 0.00000 |
| 14-052 | 0.00000 | 0.00000 | 0.00000 | 0.00000 | 0.00000 | 0.54545 | 0.66667 | 0.70000 | 0.90000 | 0.58333 | 0.77778 | 0.81818 | 0.75000 | 0.66667 | 0.33333 |
| 14-053 | 0.00000 | 0.00000 | 0.00000 | 0.00000 | 0.08333 | 0.45455 | 0.58333 | 0.50000 | 0.30000 | 0.33333 | 0.66667 | 0.54545 | 0.37500 | 0.66667 | 0.16667 |
| 14-054 | 0.00000 | 0.00000 | 0.00000 | 0.00000 | 0.00000 | 0.00000 | 0.08333 | 0.00000 | 0.00000 | 0.00000 | 0.00000 | 0.00000 | 0.00000 | 0.11111 | 0.00000 |
| 14-055 | 0.00000 | 0.00000 | 0.00000 | 0.08333 | 0.00000 | 0.00000 | 0.08333 | 0.00000 | 0.00000 | 0.00000 | 0.00000 | 0.09091 | 0.00000 | 0.00000 | 0.00000 |
| 14-056 | 0.25000 | 0.16667 | 0.00000 | 0.08333 | 0.00000 | 0.00000 | 0.00000 | 0.00000 | 0.00000 | 0.00000 | 0.00000 | 0.00000 | 0.00000 | 0.00000 | 0.00000 |
| 14-057 | 0.00000 | 0.08333 | 0.00000 | 0.00000 | 0.00000 | 0.00000 | 0.00000 | 0.00000 | 0.00000 | 0.00000 | 0.00000 | 0.00000 | 0.00000 | 0.00000 | 0.00000 |
| 14-058 | 0.00000 | 0.00000 | 0.25000 | 0.08333 | 0.00000 | 0.00000 | 0.00000 | 0.00000 | 0.00000 | 0.00000 | 0.00000 | 0.00000 | 0.00000 | 0.00000 | 0.00000 |
| 14-059 | 0.00000 | 0.08333 | 0.16667 | 0.00000 | 0.00000 | 0.00000 | 0.00000 | 0.00000 | 0.00000 | 0.08333 | 0.00000 | 0.00000 | 0.00000 | 0.00000 | 0.00000 |
| 14-060 | 0.00000 | 0.08333 | 0.00000 | 0.00000 | 0.00000 | 0.00000 | 0.00000 | 0.00000 | 0.00000 | 0.00000 | 0.00000 | 0.09091 | 0.00000 | 0.00000 | 0.16667 |
| 14-061 | 0.00000 | 0.08333 | 0.00000 | 0.00000 | 0.00000 | 0.00000 | 0.00000 | 0.10000 | 0.00000 | 0.00000 | 0.00000 | 0.09091 | 0.00000 | 0.00000 | 0.16667 |
| 14-062 | 0.00000 | 0.25000 | 0.00000 | 0.00000 | 0.00000 | 0.00000 | 0.25000 | 0.00000 | 0.00000 | 0.00000 | 0.00000 | 0.00000 | 0.00000 | 0.00000 | 0.00000 |
| 14-063 | 0.00000 | 0.00000 | 0.00000 | 0.00000 | 0.08333 | 0.00000 | 0.00000 | 0.00000 | 0.00000 | 0.00000 | 0.00000 | 0.00000 | 0.00000 | 0.00000 | 0.00000 |
| 14-064 | 0.00000 | 0.00000 | 0.00000 | 0.00000 | 0.00000 | 0.09091 | 0.00000 | 0.00000 | 0.00000 | 0.08333 | 0.00000 | 0.00000 | 0.00000 | 0.00000 | 0.00000 |
| 14-065 | 0.00000 | 0.00000 | 0.00000 | 0.00000 | 0.00000 | 0.27273 | 0.00000 | 0.00000 | 0.00000 | 0.41667 | 0.11111 | 0.00000 | 0.25000 | 0.00000 | 0.50000 |
| 14-066 | 0.00000 | 0.00000 | 0.00000 | 0.00000 | 0.08333 | 0.09091 | 0.00000 | 0.00000 | 0.00000 | 0.33333 | 0.11111 | 0.00000 | 0.12500 | 0.00000 | 0.16667 |
| 14-067 | 0.00000 | 0.00000 | 0.00000 | 0.00000 | 0.00000 | 0.27273 | 0.00000 | 0.00000 | 0.00000 | 0.41667 | 0.11111 | 0.00000 | 0.12500 | 0.00000 | 0.50000 |
| 14-068 | 0.00000 | 0.08333 | 0.00000 | 0.00000 | 0.00000 | 0.00000 | 0.00000 | 0.00000 | 0.00000 | 0.00000 | 0.00000 | 0.00000 | 0.00000 | 0.00000 | 0.00000 |
| 14-069 | 0.00000 | 0.00000 | 0.00000 | 0.08333 | 0.00000 | 0.09091 | 0.00000 | 0.00000 | 0.00000 | 0.00000 | 0.00000 | 0.00000 | 0.00000 | 0.33333 | 0.00000 |
| 14-070 | 0.00000 | 0.16667 | 0.00000 | 0.00000 | 0.00000 | 0.00000 | 0.00000 | 0.00000 | 0.00000 | 0.00000 | 0.00000 | 0.00000 | 0.00000 | 0.00000 | 0.00000 |
| 14-071 | 0.00000 | 0.08333 | 0.00000 | 0.16667 | 0.00000 | 0.09091 | 0.00000 | 0.00000 | 0.00000 | 0.00000 | 0.00000 | 0.00000 | 0.00000 | 0.33333 | 0.00000 |
| 14-072 | 0.00000 | 0.08333 | 0.00000 | 0.00000 | 0.00000 | 0.00000 | 0.08333 | 0.00000 | 0.00000 | 0.00000 | 0.00000 | 0.00000 | 0.00000 | 0.11111 | 0.00000 |
| 14-073 | 0.00000 | 0.00000 | 0.00000 | 0.08333 | 0.00000 | 0.00000 | 0.08333 | 0.00000 | 0.00000 | 0.00000 | 0.00000 | 0.00000 | 0.00000 | 0.11111 | 0.00000 |
| 14-074 | 0.00000 | 0.16667 | 0.00000 | 0.00000 | 0.00000 | 0.00000 | 0.00000 | 0.00000 | 0.00000 | 0.00000 | 0.00000 | 0.00000 | 0.00000 | 0.00000 | 0.00000 |
| 14-075 | 0.00000 | 0.00000 | 0.08333 | 0.08333 | 0.00000 | 0.00000 | 0.00000 | 0.00000 | 0.00000 | 0.00000 | 0.00000 | 0.09091 | 0.00000 | 0.00000 | 0.00000 |
| 14-076 | 0.00000 | 0.00000 | 0.00000 | 0.08333 | 0.00000 | 0.00000 | 0.00000 | 0.00000 | 0.00000 | 0.00000 | 0.00000 | 0.09091 | 0.00000 | 0.00000 | 0.00000 |
| 14-077 | 0.00000 | 0.16667 | 0.00000 | 0.00000 | 0.00000 | 0.00000 | 0.16667 | 0.00000 | 0.00000 | 0.00000 | 0.00000 | 0.00000 | 0.00000 | 0.00000 | 0.00000 |
| 14-078 | 0.00000 | 0.08333 | 0.00000 | 0.00000 | 0.00000 | 0.09091 | 0.16667 | 0.00000 | 0.00000 | 0.16667 | 0.00000 | 0.00000 | 0.00000 | 0.00000 | 0.00000 |
| 14-079 | 0.00000 | 0.00000 | 0.00000 | 0.00000 | 0.00000 | 0.00000 | 0.00000 | 0.00000 | 0.00000 | 0.00000 | 0.00000 | 0.09091 | 0.00000 | 0.00000 | 0.00000 |
| 14-080 | 0.00000 | 0.00000 | 0.00000 | 0.00000 | 0.00000 | 0.00000 | 0.00000 | 0.00000 | 0.00000 | 0.08333 | 0.00000 | 0.00000 | 0.00000 | 0.00000 | 0.00000 |
| 14-081 | 0.00000 | 0.00000 | 0.00000 | 0.00000 | 0.00000 | 0.00000 | 0.00000 | 0.00000 | 0.00000 | 0.08333 | 0.00000 | 0.00000 | 0.00000 | 0.00000 | 0.00000 |
| 14-082 | 0.25000 | 0.08333 | 0.25000 | 0.16667 | 0.25000 | 0.09091 | 0.08333 | 0.00000 | 0.00000 | 0.08333 | 0.00000 | 0.00000 | 0.00000 | 0.00000 | 0.00000 |
| 14-083 | 0.00000 | 0.00000 | 0.00000 | 0.00000 | 0.00000 | 0.00000 | 0.33333 | 0.00000 | 0.00000 | 0.00000 | 0.00000 | 0.09091 | 0.00000 | 0.00000 | 0.00000 |
| 14-084 | 0.00000 | 0.00000 | 0.00000 | 0.00000 | 0.00000 | 0.09091 | 0.00000 | 0.00000 | 0.00000 | 0.00000 | 0.00000 | 0.00000 | 0.00000 | 0.00000 | 0.00000 |
| 14-085 | 0.00000 | 0.00000 | 0.00000 | 0.00000 | 0.08333 | 0.09091 | 0.00000 | 0.00000 | 0.00000 | 0.00000 | 0.00000 | 0.00000 | 0.00000 | 0.00000 | 0.00000 |
| 14-086 | 0.00000 | 0.00000 | 0.00000 | 0.00000 | 0.00000 | 0.00000 | 0.00000 | 0.00000 | 0.00000 | 0.00000 | 0.00000 | 0.00000 | 0.00000 | 0.11111 | 0.00000 |
| 14-087 | 0.00000 | 0.00000 | 0.00000 | 0.00000 | 0.00000 | 0.00000 | 0.00000 | 0.00000 | 0.00000 | 0.00000 | 0.00000 | 0.00000 | 0.00000 | 0.11111 | 0.00000 |
| 14-088 | 0.00000 | 0.00000 | 0.00000 | 0.00000 | 0.00000 | 0.00000 | 0.00000 | 0.00000 | 0.00000 | 0.00000 | 0.00000 | 0.00000 | 0.12500 | 0.00000 | 0.00000 |
| 14-089 | 0.00000 | 0.00000 | 0.00000 | 0.00000 | 0.00000 | 0.00000 | 0.00000 | 0.00000 | 0.00000 | 0.00000 | 0.00000 | 0.00000 | 0.12500 | 0.00000 | 0.00000 |
| 14-090 | 0.00000 | 0.00000 | 0.00000 | 0.00000 | 0.08333 | 0.00000 | 0.00000 | 0.00000 | 0.00000 | 0.00000 | 0.00000 | 0.00000 | 0.00000 | 0.00000 | 0.00000 |
| 14-091 | 0.00000 | 0.16667 | 0.00000 | 0.00000 | 0.00000 | 0.00000 | 0.00000 | 0.00000 | 0.00000 | 0.00000 | 0.00000 | 0.00000 | 0.00000 | 0.00000 | 0.00000 |
| 14-092 | 0.00000 | 0.00000 | 0.08333 | 0.00000 | 0.00000 | 0.00000 | 0.00000 | 0.00000 | 0.00000 | 0.00000 | 0.00000 | 0.00000 | 0.12500 | 0.00000 | 0.00000 |
| 14-093 | 0.00000 | 0.00000 | 0.08333 | 0.00000 | 0.00000 | 0.00000 | 0.00000 | 0.00000 | 0.00000 | 0.00000 | 0.00000 | 0.00000 | 0.00000 | 0.00000 | 0.00000 |
| 14-094 | 0.00000 | 0.00000 | 0.00000 | 0.00000 | 0.08333 | 0.00000 | 0.00000 | 0.10000 | 0.00000 | 0.00000 | 0.00000 | 0.09091 | 0.00000 | 0.00000 | 0.16667 |
| 14-095 | 0.00000 | 0.00000 | 0.00000 | 0.00000 | 0.08333 | 0.00000 | 0.00000 | 0.10000 | 0.00000 | 0.00000 | 0.00000 | 0.00000 | 0.00000 | 0.00000 | 0.16667 |
| 14-096 | 0.00000 | 0.00000 | 0.00000 | 0.00000 | 0.08333 | 0.00000 | 0.00000 | 0.00000 | 0.00000 | 0.00000 | 0.00000 | 0.00000 | 0.00000 | 0.00000 | 0.00000 |
| 14-097 | 0.00000 | 0.00000 | 0.00000 | 0.00000 | 0.08333 | 0.00000 | 0.00000 | 0.00000 | 0.00000 | 0.00000 | 0.00000 | 0.00000 | 0.00000 | 0.00000 | 0.00000 |
| 14-098 | 0.00000 | 0.08333 | 0.00000 | 0.00000 | 0.00000 | 0.00000 | 0.00000 | 0.00000 | 0.00000 | 0.00000 | 0.00000 | 0.00000 | 0.00000 | 0.00000 | 0.00000 |
| 14-099 | 0.00000 | 0.08333 | 0.00000 | 0.00000 | 0.00000 | 0.00000 | 0.00000 | 0.00000 | 0.00000 | 0.00000 | 0.00000 | 0.00000 | 0.00000 | 0.00000 | 0.00000 |
| 14-100 | 0.00000 | 0.00000 | 0.00000 | 0.00000 | 0.00000 | 0.00000 | 0.00000 | 0.00000 | 0.10000 | 0.00000 | 0.00000 | 0.00000 | 0.00000 | 0.00000 | 0.00000 |
| 14-101 | 0.00000 | 0.00000 | 0.00000 | 0.00000 | 0.00000 | 0.00000 | 0.00000 | 0.00000 | 0.10000 | 0.00000 | 0.00000 | 0.00000 | 0.00000 | 0.00000 | 0.00000 |
| 14-102 | 0.00000 | 0.00000 | 0.00000 | 0.00000 | 0.00000 | 0.00000 | 0.00000 | 0.00000 | 0.00000 | 0.08333 | 0.00000 | 0.00000 | 0.00000 | 0.00000 | 0.00000 |
| 14-103 | 0.00000 | 0.00000 | 0.00000 | 0.00000 | 0.00000 | 0.00000 | 0.00000 | 0.00000 | 0.00000 | 0.08333 | 0.00000 | 0.00000 | 0.00000 | 0.00000 | 0.00000 |
| 14-104 | 0.00000 | 0.00000 | 0.00000 | 0.00000 | 0.08333 | 0.00000 | 0.00000 | 0.00000 | 0.00000 | 0.00000 | 0.00000 | 0.00000 | 0.00000 | 0.00000 | 0.00000 |
| 16-001 | 0.25000 | 0.16667 | 0.08333 | 0.09091 | 0.25000 | 0.10000 | 0.50000 | 0.16667 | 0.00000 | 0.00000 | 0.00000 | 0.00000 | 0.00000 | 0.00000 | 0.00000 |
| 16-002 | 0.25000 | 0.08333 | 0.33333 | 0.00000 | 0.00000 | 0.10000 | 0.00000 | 0.08333 | 1.00000 | 0.50000 | 0.20000 | 0.12500 | 0.00000 | 0.00000 | 0.00000 |
| 16-003 | 0.25000 | 0.00000 | 0.08333 | 0.00000 | 0.08333 | 0.00000 | 0.08333 | 0.25000 | 0.10000 | 0.00000 | 0.00000 | 0.00000 | 0.00000 | 0.00000 | 0.00000 |
| 16-004 | 0.25000 | 0.41667 | 0.58333 | 0.27273 | 0.50000 | 0.10000 | 0.08333 | 0.08333 | 0.00000 | 0.00000 | 0.00000 | 0.00000 | 0.00000 | 0.00000 | 0.00000 |
| 16-005 | 0.00000 | 0.00000 | 0.08333 | 0.00000 | 0.00000 | 0.00000 | 0.00000 | 0.00000 | 0.00000 | 0.00000 | 0.00000 | 0.00000 | 0.00000 | 0.00000 | 0.00000 |
| 16-006 | 0.00000 | 0.08333 | 0.16667 | 0.27273 | 0.58333 | 0.00000 | 0.00000 | 0.00000 | 0.00000 | 0.10000 | 0.00000 | 0.00000 | 0.00000 | 0.11111 | 0.00000 |
| 16-007 | 0.25000 | 0.00000 | 0.00000 | 0.09091 | 0.16667 | 0.40000 | 0.83333 | 0.41667 | 0.20000 | 0.30000 | 0.20000 | 0.00000 | 0.20000 | 0.00000 | 0.16667 |
| 16-008 | 0.00000 | 0.00000 | 0.00000 | 0.09091 | 0.00000 | 0.10000 | 0.50000 | 0.41667 | 0.00000 | 0.00000 | 0.00000 | 0.00000 | 0.00000 | 0.00000 | 0.33333 |
| 16-009 | 0.00000 | 0.00000 | 0.08333 | 0.00000 | 0.00000 | 0.00000 | 0.00000 | 0.08333 | 0.00000 | 0.00000 | 0.00000 | 0.00000 | 0.00000 | 0.00000 | 0.00000 |
| 16-010 | 0.00000 | 0.08333 | 0.16667 | 0.09091 | 0.00000 | 0.00000 | 0.16667 | 0.00000 | 0.00000 | 0.00000 | 0.00000 | 0.00000 | 0.00000 | 0.00000 | 0.00000 |
| 16-011 | 0.00000 | 0.33333 | 0.16667 | 0.27273 | 0.25000 | 0.40000 | 0.00000 | 0.08333 | 0.00000 | 0.00000 | 0.00000 | 0.00000 | 0.00000 | 0.00000 | 0.00000 |
| 16-012 | 0.00000 | 0.00000 | 0.08333 | 0.00000 | 0.00000 | 0.10000 | 0.50000 | 0.33333 | 0.10000 | 0.10000 | 0.20000 | 0.00000 | 0.00000 | 0.00000 | 0.00000 |
| 16-013 | 0.00000 | 0.00000 | 0.00000 | 0.00000 | 0.08333 | 0.00000 | 0.25000 | 0.00000 | 0.00000 | 0.00000 | 0.00000 | 0.00000 | 0.00000 | 0.00000 | 0.00000 |
| 16-014 | 0.00000 | 0.00000 | 0.16667 | 0.00000 | 0.00000 | 0.10000 | 0.41667 | 0.00000 | 0.00000 | 0.00000 | 0.00000 | 0.00000 | 0.00000 | 0.11111 | 0.00000 |
| 16-015 | 0.00000 | 0.00000 | 0.08333 | 0.09091 | 0.16667 | 0.00000 | 0.08333 | 0.00000 | 0.00000 | 0.00000 | 0.00000 | 0.00000 | 0.00000 | 0.00000 | 0.00000 |
| 16-016 | 0.00000 | 0.00000 | 0.08333 | 0.00000 | 0.08333 | 0.30000 | 0.41667 | 0.16667 | 0.00000 | 0.00000 | 0.00000 | 0.00000 | 0.00000 | 0.00000 | 0.00000 |
| 16-017 | 0.00000 | 0.00000 | 0.00000 | 0.00000 | 0.00000 | 0.10000 | 0.08333 | 0.08333 | 0.00000 | 0.00000 | 0.00000 | 0.00000 | 0.00000 | 0.00000 | 0.00000 |
| 16-018 | 0.00000 | 0.08333 | 0.08333 | 0.09091 | 0.08333 | 0.10000 | 0.33333 | 0.00000 | 0.00000 | 0.10000 | 0.00000 | 0.00000 | 0.00000 | 0.00000 | 0.00000 |
| 16-019 | 0.00000 | 0.00000 | 0.08333 | 0.00000 | 0.00000 | 0.00000 | 0.16667 | 0.08333 | 0.00000 | 0.10000 | 0.00000 | 0.00000 | 0.00000 | 0.00000 | 0.00000 |
| 16-020 | 0.00000 | 0.00000 | 0.00000 | 0.00000 | 0.08333 | 0.00000 | 0.08333 | 0.08333 | 0.00000 | 0.00000 | 0.00000 | 0.00000 | 0.00000 | 0.00000 | 0.00000 |
| 16-021 | 0.00000 | 0.00000 | 0.00000 | 0.09091 | 0.00000 | 0.10000 | 0.16667 | 0.00000 | 0.00000 | 0.00000 | 0.00000 | 0.00000 | 0.00000 | 0.00000 | 0.00000 |
| 16-022 | 0.00000 | 0.16667 | 0.08333 | 0.09091 | 0.25000 | 0.10000 | 0.16667 | 0.00000 | 0.00000 | 0.00000 | 0.00000 | 0.00000 | 0.00000 | 0.00000 | 0.00000 |
| 16-023 | 0.00000 | 0.16667 | 0.00000 | 0.00000 | 0.08333 | 0.10000 | 0.00000 | 0.00000 | 0.00000 | 0.00000 | 0.00000 | 0.00000 | 0.00000 | 0.00000 | 0.00000 |
| 16-024 | 0.00000 | 0.00000 | 0.00000 | 0.00000 | 0.00000 | 0.00000 | 0.08333 | 0.00000 | 0.00000 | 0.10000 | 0.00000 | 0.00000 | 0.00000 | 0.00000 | 0.00000 |
| 16-025 | 0.00000 | 0.00000 | 0.08333 | 0.00000 | 0.00000 | 0.10000 | 0.50000 | 0.00000 | 0.00000 | 0.10000 | 0.00000 | 0.00000 | 0.00000 | 0.11111 | 0.00000 |
| 16-026 | 0.00000 | 0.00000 | 0.08333 | 0.00000 | 0.00000 | 0.10000 | 0.58333 | 0.33333 | 0.00000 | 0.10000 | 0.00000 | 0.00000 | 0.00000 | 0.11111 | 0.00000 |
| 16-027 | 0.00000 | 0.08333 | 0.08333 | 0.00000 | 0.08333 | 0.10000 | 0.16667 | 0.25000 | 0.10000 | 0.10000 | 0.00000 | 0.00000 | 0.00000 | 0.00000 | 0.00000 |
| 16-028 | 0.25000 | 0.50000 | 0.41667 | 0.27273 | 0.33333 | 0.10000 | 0.08333 | 0.08333 | 0.00000 | 0.00000 | 0.00000 | 0.00000 | 0.00000 | 0.00000 | 0.00000 |
| 16-029 | 0.00000 | 0.50000 | 0.16667 | 0.27273 | 0.41667 | 0.20000 | 0.08333 | 0.08333 | 0.00000 | 0.00000 | 0.00000 | 0.00000 | 0.00000 | 0.00000 | 0.00000 |
| 16-030 | 0.00000 | 0.00000 | 0.08333 | 0.00000 | 0.08333 | 0.20000 | 0.41667 | 0.25000 | 0.60000 | 0.00000 | 0.00000 | 0.12500 | 0.00000 | 0.00000 | 0.16667 |
| 16-031 | 0.00000 | 0.00000 | 0.08333 | 0.00000 | 0.08333 | 0.20000 | 0.16667 | 0.41667 | 0.60000 | 0.10000 | 0.00000 | 0.12500 | 0.20000 | 0.00000 | 0.16667 |
| 16-032 | 0.00000 | 0.08333 | 0.25000 | 0.00000 | 0.33333 | 0.00000 | 0.58333 | 0.08333 | 0.10000 | 0.10000 | 0.00000 | 0.00000 | 0.00000 | 0.00000 | 0.00000 |
| 16-033 | 0.25000 | 0.58333 | 0.75000 | 0.54545 | 0.66667 | 0.50000 | 0.33333 | 0.08333 | 0.60000 | 0.00000 | 0.00000 | 0.12500 | 0.00000 | 0.11111 | 0.16667 |
| 16-034 | 0.00000 | 0.58333 | 0.58333 | 0.63636 | 0.50000 | 0.40000 | 0.41667 | 0.66667 | 0.30000 | 0.10000 | 0.00000 | 0.00000 | 0.00000 | 0.11111 | 0.00000 |
| 16-035 | 0.00000 | 0.33333 | 0.08333 | 0.09091 | 0.25000 | 0.10000 | 0.33333 | 0.08333 | 0.00000 | 0.00000 | 0.00000 | 0.00000 | 0.00000 | 0.00000 | 0.16667 |
| 16-036 | 0.50000 | 0.50000 | 0.41667 | 0.45455 | 0.33333 | 0.20000 | 0.58333 | 0.08333 | 0.00000 | 0.00000 | 0.00000 | 0.00000 | 0.20000 | 0.00000 | 0.00000 |
| 16-037 | 0.50000 | 0.50000 | 0.33333 | 0.45455 | 0.25000 | 0.10000 | 0.08333 | 0.08333 | 0.00000 | 0.00000 | 0.00000 | 0.00000 | 0.00000 | 0.00000 | 0.00000 |
| 16-038 | 0.00000 | 0.00000 | 0.00000 | 0.00000 | 0.00000 | 0.20000 | 0.16667 | 0.00000 | 0.00000 | 0.00000 | 0.00000 | 0.00000 | 0.00000 | 0.00000 | 0.00000 |
| 16-039 | 0.00000 | 0.00000 | 0.00000 | 0.27273 | 0.00000 | 0.00000 | 0.00000 | 0.00000 | 0.00000 | 0.00000 | 0.00000 | 0.00000 | 0.00000 | 0.00000 | 0.00000 |
| 16-040 | 0.00000 | 0.00000 | 0.00000 | 0.09091 | 0.00000 | 0.00000 | 0.25000 | 0.00000 | 0.10000 | 0.00000 | 0.00000 | 0.00000 | 0.00000 | 0.00000 | 0.00000 |
| 16-041 | 0.00000 | 0.00000 | 0.00000 | 0.09091 | 0.00000 | 0.00000 | 0.00000 | 0.00000 | 0.00000 | 0.00000 | 0.00000 | 0.00000 | 0.00000 | 0.00000 | 0.00000 |
| 16-042 | 0.00000 | 0.00000 | 0.00000 | 0.09091 | 0.00000 | 0.30000 | 0.50000 | 0.16667 | 0.30000 | 0.10000 | 0.00000 | 0.00000 | 0.00000 | 0.00000 | 0.00000 |
| 16-043 | 0.00000 | 0.00000 | 0.00000 | 0.00000 | 0.00000 | 0.20000 | 0.41667 | 0.16667 | 0.00000 | 0.10000 | 0.00000 | 0.00000 | 0.00000 | 0.00000 | 0.00000 |
| 16-044 | 0.00000 | 0.00000 | 0.00000 | 0.00000 | 0.08333 | 0.00000 | 0.00000 | 0.00000 | 0.00000 | 0.00000 | 0.00000 | 0.00000 | 0.00000 | 0.00000 | 0.00000 |
| 16-045 | 0.00000 | 0.16667 | 0.16667 | 0.27273 | 0.08333 | 0.00000 | 0.33333 | 0.33333 | 0.10000 | 0.10000 | 0.20000 | 0.12500 | 0.20000 | 0.11111 | 0.00000 |
| 16-046 | 0.00000 | 0.00000 | 0.08333 | 0.00000 | 0.00000 | 0.00000 | 0.00000 | 0.25000 | 0.00000 | 0.00000 | 0.00000 | 0.00000 | 0.00000 | 0.00000 | 0.00000 |
| 16-047 | 0.00000 | 0.00000 | 0.08333 | 0.00000 | 0.00000 | 0.00000 | 0.08333 | 0.08333 | 0.30000 | 0.00000 | 0.00000 | 0.00000 | 0.00000 | 0.00000 | 0.00000 |
| 16-048 | 0.00000 | 0.00000 | 0.00000 | 0.00000 | 0.00000 | 0.00000 | 0.08333 | 0.16667 | 0.00000 | 0.00000 | 0.00000 | 0.00000 | 0.00000 | 0.00000 | 0.00000 |
| 16-049 | 0.00000 | 0.00000 | 0.00000 | 0.00000 | 0.00000 | 0.10000 | 0.08333 | 0.00000 | 0.00000 | 0.00000 | 0.00000 | 0.00000 | 0.00000 | 0.00000 | 0.00000 |
| 16-050 | 0.00000 | 0.41667 | 0.16667 | 0.36364 | 0.33333 | 0.00000 | 0.00000 | 0.00000 | 0.00000 | 0.00000 | 0.00000 | 0.00000 | 0.00000 | 0.00000 | 0.00000 |
| 16-051 | 0.00000 | 0.00000 | 0.00000 | 0.00000 | 0.00000 | 0.00000 | 0.08333 | 0.00000 | 0.00000 | 0.00000 | 0.00000 | 0.00000 | 0.00000 | 0.11111 | 0.00000 |
| 16-052 | 0.00000 | 0.00000 | 0.00000 | 0.09091 | 0.00000 | 0.20000 | 0.00000 | 0.25000 | 0.50000 | 0.00000 | 0.00000 | 0.00000 | 0.00000 | 0.00000 | 0.00000 |
| 16-053 | 0.25000 | 0.08333 | 0.08333 | 0.09091 | 0.16667 | 0.30000 | 0.50000 | 0.50000 | 0.30000 | 0.40000 | 0.00000 | 0.62500 | 0.00000 | 0.66667 | 0.50000 |
| 16-054 | 0.00000 | 0.00000 | 0.08333 | 0.00000 | 0.00000 | 0.10000 | 0.16667 | 0.00000 | 0.00000 | 0.00000 | 0.00000 | 0.00000 | 0.20000 | 0.00000 | 0.00000 |
| 16-055 | 0.00000 | 0.00000 | 0.00000 | 0.00000 | 0.00000 | 0.00000 | 0.08333 | 0.00000 | 0.00000 | 0.00000 | 0.00000 | 0.00000 | 0.00000 | 0.00000 | 0.00000 |
| 16-056 | 0.00000 | 0.00000 | 0.00000 | 0.00000 | 0.16667 | 0.10000 | 0.16667 | 0.16667 | 0.10000 | 0.10000 | 0.20000 | 0.12500 | 0.00000 | 0.11111 | 0.00000 |
| 16-057 | 0.00000 | 0.00000 | 0.00000 | 0.18182 | 0.00000 | 0.00000 | 0.08333 | 0.08333 | 0.00000 | 0.00000 | 0.20000 | 0.00000 | 0.00000 | 0.00000 | 0.00000 |
| 16-058 | 0.00000 | 0.00000 | 0.08333 | 0.00000 | 0.00000 | 0.00000 | 0.08333 | 0.16667 | 0.10000 | 0.00000 | 0.00000 | 0.12500 | 0.00000 | 0.00000 | 0.00000 |
| 16-059 | 0.00000 | 0.16667 | 0.08333 | 0.09091 | 0.00000 | 0.00000 | 0.16667 | 0.25000 | 0.40000 | 0.00000 | 0.00000 | 0.00000 | 0.00000 | 0.00000 | 0.00000 |
| 16-060 | 0.00000 | 0.16667 | 0.16667 | 0.09091 | 0.00000 | 0.00000 | 0.00000 | 0.08333 | 0.40000 | 0.10000 | 0.00000 | 0.00000 | 0.00000 | 0.00000 | 0.00000 |
| 16-061 | 0.00000 | 0.00000 | 0.00000 | 0.09091 | 0.00000 | 0.10000 | 0.08333 | 0.08333 | 0.20000 | 0.10000 | 0.00000 | 0.00000 | 0.00000 | 0.00000 | 0.00000 |
| 16-062 | 0.00000 | 0.00000 | 0.00000 | 0.00000 | 0.00000 | 0.10000 | 0.00000 | 0.00000 | 0.00000 | 0.00000 | 0.00000 | 0.00000 | 0.20000 | 0.00000 | 0.00000 |
| 16-063 | 0.00000 | 0.00000 | 0.00000 | 0.00000 | 0.00000 | 0.00000 | 0.00000 | 0.00000 | 0.00000 | 0.00000 | 0.00000 | 0.00000 | 0.20000 | 0.00000 | 0.00000 |
| 16-064 | 0.00000 | 0.00000 | 0.08333 | 0.00000 | 0.00000 | 0.00000 | 0.08333 | 0.00000 | 0.00000 | 0.00000 | 0.00000 | 0.00000 | 0.00000 | 0.00000 | 0.00000 |
| 16-065 | 0.00000 | 0.00000 | 0.00000 | 0.09091 | 0.16667 | 0.10000 | 0.00000 | 0.00000 | 0.00000 | 0.10000 | 0.00000 | 0.00000 | 0.20000 | 0.00000 | 0.00000 |
| 16-066 | 0.00000 | 0.00000 | 0.00000 | 0.00000 | 0.16667 | 0.10000 | 0.08333 | 0.16667 | 0.20000 | 0.00000 | 0.00000 | 0.00000 | 0.00000 | 0.00000 | 0.00000 |
| 16-067 | 0.25000 | 0.00000 | 0.00000 | 0.00000 | 0.00000 | 0.10000 | 0.00000 | 0.16667 | 0.20000 | 0.00000 | 0.00000 | 0.00000 | 0.00000 | 0.00000 | 0.00000 |
| 16-068 | 0.00000 | 0.00000 | 0.00000 | 0.09091 | 0.00000 | 0.00000 | 0.00000 | 0.00000 | 0.00000 | 0.00000 | 0.00000 | 0.00000 | 0.00000 | 0.00000 | 0.00000 |
| 16-069 | 0.25000 | 0.25000 | 0.41667 | 0.18182 | 0.25000 | 0.10000 | 0.00000 | 0.00000 | 0.00000 | 0.00000 | 0.00000 | 0.00000 | 0.00000 | 0.00000 | 0.00000 |
| 16-070 | 0.00000 | 0.00000 | 0.00000 | 0.27273 | 0.33333 | 0.10000 | 0.08333 | 0.00000 | 0.00000 | 0.00000 | 0.00000 | 0.00000 | 0.00000 | 0.00000 | 0.00000 |
| 16-071 | 0.00000 | 0.00000 | 0.00000 | 0.09091 | 0.08333 | 0.00000 | 0.08333 | 0.00000 | 0.00000 | 0.10000 | 0.00000 | 0.00000 | 0.20000 | 0.00000 | 0.50000 |
| 16-072 | 0.00000 | 0.00000 | 0.00000 | 0.18182 | 0.08333 | 0.00000 | 0.00000 | 0.00000 | 0.00000 | 0.00000 | 0.00000 | 0.00000 | 0.00000 | 0.00000 | 0.00000 |
| 16-073 | 0.00000 | 0.00000 | 0.00000 | 0.00000 | 0.00000 | 0.00000 | 0.00000 | 0.00000 | 0.00000 | 0.10000 | 0.00000 | 0.00000 | 0.00000 | 0.00000 | 0.00000 |
| 16-074 | 0.00000 | 0.00000 | 0.00000 | 0.00000 | 0.00000 | 0.00000 | 0.08333 | 0.00000 | 0.00000 | 0.00000 | 0.00000 | 0.00000 | 0.00000 | 0.00000 | 0.00000 |
| 16-075 | 0.00000 | 0.08333 | 0.00000 | 0.00000 | 0.00000 | 0.00000 | 0.16667 | 0.00000 | 0.00000 | 0.00000 | 0.00000 | 0.00000 | 0.00000 | 0.00000 | 0.00000 |
| 16-076 | 0.00000 | 0.16667 | 0.00000 | 0.00000 | 0.00000 | 0.00000 | 0.16667 | 0.00000 | 0.00000 | 0.00000 | 0.00000 | 0.00000 | 0.00000 | 0.00000 | 0.00000 |
| 16-077 | 0.25000 | 0.08333 | 0.00000 | 0.00000 | 0.25000 | 0.00000 | 0.00000 | 0.00000 | 0.00000 | 0.00000 | 0.00000 | 0.00000 | 0.00000 | 0.00000 | 0.00000 |
| 16-078 | 0.00000 | 0.00000 | 0.00000 | 0.00000 | 0.00000 | 0.10000 | 0.00000 | 0.00000 | 0.10000 | 0.10000 | 0.00000 | 0.00000 | 0.20000 | 0.00000 | 0.00000 |
| 16-079 | 0.00000 | 0.08333 | 0.08333 | 0.00000 | 0.00000 | 0.10000 | 0.00000 | 0.00000 | 0.00000 | 0.00000 | 0.00000 | 0.00000 | 0.20000 | 0.00000 | 0.00000 |
| 16-080 | 0.00000 | 0.08333 | 0.00000 | 0.00000 | 0.00000 | 0.00000 | 0.08333 | 0.00000 | 0.00000 | 0.00000 | 0.00000 | 0.00000 | 0.20000 | 0.00000 | 0.00000 |
| 16-081 | 0.00000 | 0.16667 | 0.08333 | 0.00000 | 0.00000 | 0.00000 | 0.33333 | 0.00000 | 0.00000 | 0.00000 | 0.00000 | 0.00000 | 0.00000 | 0.00000 | 0.00000 |
| 16-082 | 0.00000 | 0.00000 | 0.08333 | 0.00000 | 0.00000 | 0.10000 | 0.41667 | 0.33333 | 0.20000 | 0.00000 | 0.00000 | 0.00000 | 0.00000 | 0.00000 | 0.00000 |
| 16-083 | 0.00000 | 0.00000 | 0.00000 | 0.00000 | 0.00000 | 0.10000 | 0.58333 | 0.25000 | 0.20000 | 0.00000 | 0.00000 | 0.00000 | 0.00000 | 0.00000 | 0.00000 |
| 16-084 | 0.00000 | 0.16667 | 0.00000 | 0.00000 | 0.08333 | 0.00000 | 0.25000 | 0.00000 | 0.00000 | 0.00000 | 0.00000 | 0.00000 | 0.00000 | 0.00000 | 0.00000 |
| 16-085 | 0.00000 | 0.08333 | 0.00000 | 0.00000 | 0.08333 | 0.00000 | 0.25000 | 0.00000 | 0.00000 | 0.00000 | 0.00000 | 0.00000 | 0.00000 | 0.00000 | 0.00000 |
| 16-086 | 0.00000 | 0.00000 | 0.00000 | 0.00000 | 0.00000 | 0.00000 | 0.16667 | 0.00000 | 0.00000 | 0.00000 | 0.00000 | 0.00000 | 0.00000 | 0.00000 | 0.00000 |
| 16-087 | 0.00000 | 0.08333 | 0.00000 | 0.00000 | 0.00000 | 0.00000 | 0.00000 | 0.00000 | 0.00000 | 0.00000 | 0.00000 | 0.00000 | 0.00000 | 0.00000 | 0.16667 |
| 16-088 | 0.00000 | 0.00000 | 0.00000 | 0.00000 | 0.00000 | 0.10000 | 0.25000 | 0.00000 | 0.00000 | 0.00000 | 0.00000 | 0.12500 | 0.00000 | 0.00000 | 0.00000 |
| 16-089 | 0.00000 | 0.00000 | 0.00000 | 0.00000 | 0.00000 | 0.30000 | 0.25000 | 0.75000 | 0.40000 | 0.10000 | 0.00000 | 0.12500 | 0.20000 | 0.00000 | 0.00000 |
| 16-090 | 0.00000 | 0.08333 | 0.00000 | 0.00000 | 0.00000 | 0.40000 | 0.08333 | 0.75000 | 0.30000 | 0.10000 | 0.00000 | 0.00000 | 0.20000 | 0.00000 | 0.00000 |
| 16-091 | 0.00000 | 0.00000 | 0.00000 | 0.00000 | 0.00000 | 0.10000 | 0.00000 | 0.00000 | 0.00000 | 0.10000 | 0.00000 | 0.00000 | 0.00000 | 0.00000 | 0.00000 |
| 16-092 | 0.25000 | 0.08333 | 0.25000 | 0.18182 | 0.00000 | 0.30000 | 0.25000 | 0.00000 | 0.30000 | 0.10000 | 0.00000 | 0.12500 | 0.00000 | 0.00000 | 0.00000 |
| 16-093 | 0.00000 | 0.00000 | 0.16667 | 0.18182 | 0.00000 | 0.20000 | 0.25000 | 0.00000 | 0.30000 | 0.00000 | 0.00000 | 0.00000 | 0.00000 | 0.00000 | 0.00000 |
| 16-094 | 0.00000 | 0.08333 | 0.00000 | 0.00000 | 0.00000 | 0.00000 | 0.00000 | 0.00000 | 0.00000 | 0.00000 | 0.00000 | 0.00000 | 0.00000 | 0.00000 | 0.00000 |
| 16-095 | 0.00000 | 0.08333 | 0.00000 | 0.00000 | 0.00000 | 0.00000 | 0.00000 | 0.00000 | 0.00000 | 0.00000 | 0.00000 | 0.00000 | 0.00000 | 0.00000 | 0.00000 |
| 16-096 | 0.00000 | 0.00000 | 0.00000 | 0.18182 | 0.00000 | 0.10000 | 0.16667 | 0.00000 | 0.00000 | 0.00000 | 0.00000 | 0.00000 | 0.00000 | 0.00000 | 0.00000 |
| 16-097 | 0.00000 | 0.08333 | 0.00000 | 0.00000 | 0.00000 | 0.10000 | 0.16667 | 0.00000 | 0.00000 | 0.00000 | 0.00000 | 0.00000 | 0.00000 | 0.00000 | 0.00000 |
| 16-098 | 0.00000 | 0.00000 | 0.00000 | 0.09091 | 0.00000 | 0.00000 | 0.00000 | 0.00000 | 0.00000 | 0.10000 | 0.00000 | 0.00000 | 0.00000 | 0.00000 | 0.00000 |
| 16-099 | 0.00000 | 0.00000 | 0.00000 | 0.00000 | 0.00000 | 0.20000 | 0.00000 | 0.00000 | 0.00000 | 0.20000 | 0.00000 | 0.00000 | 0.20000 | 0.00000 | 0.00000 |
| 16-100 | 0.00000 | 0.00000 | 0.00000 | 0.09091 | 0.00000 | 0.20000 | 0.25000 | 0.16667 | 0.10000 | 0.30000 | 0.00000 | 0.25000 | 0.20000 | 0.00000 | 0.00000 |
| 16-101 | 0.00000 | 0.08333 | 0.00000 | 0.09091 | 0.00000 | 0.00000 | 0.00000 | 0.00000 | 0.00000 | 0.00000 | 0.00000 | 0.00000 | 0.00000 | 0.00000 | 0.00000 |
| 16-102 | 0.00000 | 0.00000 | 0.00000 | 0.18182 | 0.00000 | 0.00000 | 0.08333 | 0.00000 | 0.20000 | 0.00000 | 0.00000 | 0.00000 | 0.00000 | 0.11111 | 0.00000 |
| 16-103 | 0.00000 | 0.00000 | 0.00000 | 0.09091 | 0.00000 | 0.00000 | 0.00000 | 0.00000 | 0.00000 | 0.00000 | 0.00000 | 0.00000 | 0.00000 | 0.00000 | 0.00000 |
| 16-104 | 0.00000 | 0.08333 | 0.00000 | 0.09091 | 0.00000 | 0.00000 | 0.00000 | 0.00000 | 0.00000 | 0.00000 | 0.00000 | 0.00000 | 0.00000 | 0.00000 | 0.00000 |
| 16-105 | 0.00000 | 0.08333 | 0.00000 | 0.00000 | 0.00000 | 0.00000 | 0.00000 | 0.00000 | 0.20000 | 0.10000 | 0.00000 | 0.00000 | 0.00000 | 0.00000 | 0.00000 |
| 16-106 | 0.00000 | 0.00000 | 0.00000 | 0.09091 | 0.00000 | 0.00000 | 0.00000 | 0.00000 | 0.10000 | 0.10000 | 0.00000 | 0.00000 | 0.00000 | 0.00000 | 0.00000 |
| 16-107 | 0.00000 | 0.00000 | 0.00000 | 0.00000 | 0.00000 | 0.00000 | 0.25000 | 0.00000 | 0.00000 | 0.00000 | 0.00000 | 0.00000 | 0.00000 | 0.00000 | 0.00000 |
| 16-108 | 0.00000 | 0.00000 | 0.00000 | 0.09091 | 0.00000 | 0.00000 | 0.00000 | 0.00000 | 0.00000 | 0.00000 | 0.00000 | 0.00000 | 0.00000 | 0.00000 | 0.00000 |
| 16-109 | 0.00000 | 0.00000 | 0.00000 | 0.09091 | 0.00000 | 0.00000 | 0.00000 | 0.00000 | 0.00000 | 0.10000 | 0.00000 | 0.00000 | 0.00000 | 0.00000 | 0.00000 |
| 16-110 | 0.00000 | 0.00000 | 0.16667 | 0.00000 | 0.00000 | 0.00000 | 0.00000 | 0.00000 | 0.10000 | 0.00000 | 0.00000 | 0.00000 | 0.00000 | 0.00000 | 0.00000 |
| 16-111 | 0.00000 | 0.00000 | 0.08333 | 0.00000 | 0.00000 | 0.00000 | 0.00000 | 0.00000 | 0.00000 | 0.10000 | 0.00000 | 0.00000 | 0.00000 | 0.00000 | 0.00000 |
| 16-112 | 0.00000 | 0.00000 | 0.08333 | 0.00000 | 0.08333 | 0.00000 | 0.08333 | 0.00000 | 0.00000 | 0.00000 | 0.00000 | 0.00000 | 0.00000 | 0.00000 | 0.00000 |
| 16-113 | 0.00000 | 0.08333 | 0.08333 | 0.09091 | 0.00000 | 0.10000 | 0.16667 | 0.00000 | 0.00000 | 0.00000 | 0.20000 | 0.12500 | 0.00000 | 0.00000 | 0.16667 |
| 16-114 | 0.00000 | 0.00000 | 0.00000 | 0.09091 | 0.08333 | 0.00000 | 0.00000 | 0.00000 | 0.00000 | 0.00000 | 0.00000 | 0.00000 | 0.00000 | 0.00000 | 0.00000 |
| 16-115 | 0.00000 | 0.00000 | 0.00000 | 0.09091 | 0.16667 | 0.00000 | 0.00000 | 0.00000 | 0.00000 | 0.00000 | 0.00000 | 0.00000 | 0.00000 | 0.00000 | 0.00000 |
| 16-116 | 0.00000 | 0.00000 | 0.00000 | 0.00000 | 0.00000 | 0.00000 | 0.16667 | 0.00000 | 0.00000 | 0.00000 | 0.00000 | 0.37500 | 0.00000 | 0.00000 | 0.00000 |
| 16-117 | 0.00000 | 0.00000 | 0.00000 | 0.00000 | 0.00000 | 0.00000 | 0.00000 | 0.00000 | 0.00000 | 0.00000 | 0.00000 | 0.00000 | 0.20000 | 0.00000 | 0.00000 |
| 16-118 | 0.00000 | 0.00000 | 0.00000 | 0.00000 | 0.25000 | 0.00000 | 0.00000 | 0.00000 | 0.00000 | 0.00000 | 0.00000 | 0.00000 | 0.00000 | 0.00000 | 0.00000 |
| 16-119 | 0.00000 | 0.00000 | 0.00000 | 0.00000 | 0.00000 | 0.10000 | 0.00000 | 0.00000 | 0.00000 | 0.00000 | 0.00000 | 0.00000 | 0.20000 | 0.00000 | 0.00000 |
| 16-120 | 0.00000 | 0.00000 | 0.00000 | 0.00000 | 0.08333 | 0.10000 | 0.00000 | 0.00000 | 0.10000 | 0.10000 | 0.00000 | 0.00000 | 0.00000 | 0.00000 | 0.00000 |
| 16-121 | 0.00000 | 0.08333 | 0.00000 | 0.09091 | 0.00000 | 0.00000 | 0.08333 | 0.00000 | 0.00000 | 0.00000 | 0.00000 | 0.00000 | 0.00000 | 0.00000 | 0.00000 |
| 16-122 | 0.00000 | 0.08333 | 0.00000 | 0.00000 | 0.00000 | 0.00000 | 0.00000 | 0.00000 | 0.00000 | 0.00000 | 0.00000 | 0.00000 | 0.00000 | 0.00000 | 0.00000 |
| 16-123 | 0.00000 | 0.00000 | 0.00000 | 0.09091 | 0.00000 | 0.00000 | 0.00000 | 0.00000 | 0.00000 | 0.00000 | 0.00000 | 0.00000 | 0.00000 | 0.00000 | 0.00000 |
| 16-124 | 0.00000 | 0.00000 | 0.00000 | 0.09091 | 0.00000 | 0.00000 | 0.00000 | 0.00000 | 0.00000 | 0.00000 | 0.00000 | 0.00000 | 0.00000 | 0.00000 | 0.00000 |
| 16-125 | 0.00000 | 0.00000 | 0.00000 | 0.00000 | 0.08333 | 0.00000 | 0.00000 | 0.00000 | 0.00000 | 0.00000 | 0.00000 | 0.00000 | 0.20000 | 0.00000 | 0.00000 |
| 16-126 | 0.00000 | 0.00000 | 0.00000 | 0.00000 | 0.00000 | 0.00000 | 0.00000 | 0.00000 | 0.00000 | 0.00000 | 0.00000 | 0.00000 | 0.20000 | 0.00000 | 0.00000 |
| 16-127 | 0.00000 | 0.00000 | 0.00000 | 0.00000 | 0.00000 | 0.00000 | 0.00000 | 0.00000 | 0.10000 | 0.00000 | 0.00000 | 0.00000 | 0.00000 | 0.00000 | 0.00000 |
| 16-128 | 0.00000 | 0.00000 | 0.00000 | 0.00000 | 0.00000 | 0.00000 | 0.00000 | 0.00000 | 0.10000 | 0.00000 | 0.00000 | 0.00000 | 0.00000 | 0.00000 | 0.00000 |
| 16-129 | 0.00000 | 0.00000 | 0.00000 | 0.00000 | 0.00000 | 0.00000 | 0.00000 | 0.00000 | 0.00000 | 0.10000 | 0.00000 | 0.00000 | 0.00000 | 0.00000 | 0.00000 |
| 16-130 | 0.00000 | 0.00000 | 0.00000 | 0.00000 | 0.00000 | 0.00000 | 0.00000 | 0.08333 | 0.00000 | 0.00000 | 0.00000 | 0.00000 | 0.00000 | 0.00000 | 0.00000 |
| 16-131 | 0.00000 | 0.00000 | 0.00000 | 0.00000 | 0.00000 | 0.00000 | 0.00000 | 0.00000 | 0.00000 | 0.00000 | 0.00000 | 0.12500 | 0.00000 | 0.00000 | 0.00000 |
| 16-132 | 0.00000 | 0.00000 | 0.08333 | 0.00000 | 0.00000 | 0.00000 | 0.00000 | 0.00000 | 0.00000 | 0.00000 | 0.00000 | 0.00000 | 0.20000 | 0.00000 | 0.00000 |
| 22-001 | 0.75000 | 0.41667 | 0.08333 | 0.25000 | 0.25000 | 0.11111 | 0.08333 | 0.00000 | 0.00000 | 0.00000 | 0.00000 | 0.00000 | 0.00000 | 0.10000 | 0.00000 |
| 22-002 | 0.75000 | 0.75000 | 0.75000 | 0.75000 | 0.66667 | 0.11111 | 0.08333 | 0.10000 | 0.00000 | 0.08333 | 0.11111 | 0.00000 | 0.00000 | 0.20000 | 0.00000 |
| 22-003 | 0.75000 | 0.75000 | 0.75000 | 0.75000 | 0.58333 | 0.11111 | 0.16667 | 0.10000 | 0.00000 | 0.00000 | 0.00000 | 0.00000 | 0.00000 | 0.10000 | 0.00000 |
| 22-004 | 0.00000 | 0.08333 | 0.08333 | 0.08333 | 0.08333 | 0.00000 | 0.00000 | 0.00000 | 0.00000 | 0.00000 | 0.00000 | 0.00000 | 0.00000 | 0.00000 | 0.00000 |
| 22-005 | 0.00000 | 0.08333 | 0.16667 | 0.08333 | 0.00000 | 0.00000 | 0.00000 | 0.00000 | 0.00000 | 0.00000 | 0.00000 | 0.00000 | 0.00000 | 0.00000 | 0.00000 |
| 22-006 | 0.75000 | 0.50000 | 0.08333 | 0.41667 | 0.16667 | 0.00000 | 0.08333 | 0.00000 | 0.00000 | 0.00000 | 0.00000 | 0.00000 | 0.00000 | 0.00000 | 0.00000 |
| 22-007 | 0.00000 | 0.16667 | 0.08333 | 0.33333 | 0.00000 | 0.11111 | 0.00000 | 0.00000 | 0.00000 | 0.00000 | 0.00000 | 0.00000 | 0.00000 | 0.10000 | 0.00000 |
| 22-008 | 0.25000 | 0.08333 | 0.08333 | 0.08333 | 0.16667 | 0.00000 | 0.00000 | 0.00000 | 0.00000 | 0.00000 | 0.00000 | 0.00000 | 0.00000 | 0.10000 | 0.00000 |
| 22-009 | 0.50000 | 0.75000 | 0.41667 | 0.75000 | 0.16667 | 0.11111 | 0.08333 | 0.10000 | 0.00000 | 0.00000 | 0.00000 | 0.00000 | 0.00000 | 0.10000 | 0.00000 |
| 22-010 | 0.25000 | 0.25000 | 0.08333 | 0.16667 | 0.00000 | 0.00000 | 0.08333 | 0.00000 | 0.00000 | 0.00000 | 0.00000 | 0.00000 | 0.00000 | 0.10000 | 0.00000 |
| 22-011 | 0.50000 | 0.58333 | 0.33333 | 0.58333 | 0.25000 | 0.00000 | 0.08333 | 0.00000 | 0.00000 | 0.00000 | 0.00000 | 0.00000 | 0.00000 | 0.10000 | 0.00000 |
| 22-012 | 0.00000 | 0.00000 | 0.16667 | 0.00000 | 0.00000 | 0.00000 | 0.08333 | 0.00000 | 0.00000 | 0.00000 | 0.00000 | 0.08333 | 0.00000 | 0.00000 | 0.00000 |
| 22-013 | 0.00000 | 0.00000 | 0.00000 | 0.25000 | 0.00000 | 0.00000 | 0.16667 | 0.00000 | 0.00000 | 0.00000 | 0.00000 | 0.08333 | 0.00000 | 0.10000 | 0.00000 |
| 22-014 | 0.00000 | 0.25000 | 0.25000 | 0.50000 | 0.08333 | 0.00000 | 0.16667 | 0.00000 | 0.00000 | 0.00000 | 0.00000 | 0.00000 | 0.00000 | 0.10000 | 0.00000 |
| 22-015 | 0.25000 | 0.41667 | 0.41667 | 0.66667 | 0.16667 | 0.11111 | 0.00000 | 0.00000 | 0.00000 | 0.08333 | 0.00000 | 0.00000 | 0.00000 | 0.10000 | 0.00000 |
| 22-016 | 0.75000 | 0.66667 | 0.66667 | 0.66667 | 0.66667 | 0.22222 | 0.00000 | 0.00000 | 0.00000 | 0.00000 | 0.11111 | 0.00000 | 0.00000 | 0.10000 | 0.00000 |
| 22-017 | 0.50000 | 0.33333 | 0.83333 | 0.75000 | 0.41667 | 0.11111 | 0.16667 | 0.10000 | 0.22222 | 0.58333 | 0.22222 | 0.33333 | 0.62500 | 0.40000 | 0.40000 |
| 22-018 | 0.00000 | 0.41667 | 0.00000 | 0.58333 | 0.16667 | 0.00000 | 0.00000 | 0.00000 | 0.00000 | 0.00000 | 0.00000 | 0.00000 | 0.00000 | 0.10000 | 0.00000 |
| 22-019 | 0.25000 | 0.33333 | 0.08333 | 0.83333 | 0.33333 | 0.00000 | 0.16667 | 0.10000 | 0.00000 | 0.25000 | 0.22222 | 0.08333 | 0.12500 | 0.50000 | 0.40000 |
| 22-020 | 0.00000 | 0.16667 | 0.25000 | 0.41667 | 0.08333 | 0.00000 | 0.25000 | 0.00000 | 0.22222 | 0.50000 | 0.33333 | 0.41667 | 0.25000 | 0.70000 | 0.60000 |
| 22-021 | 0.25000 | 0.25000 | 0.41667 | 0.58333 | 0.33333 | 0.00000 | 0.16667 | 0.00000 | 0.00000 | 0.25000 | 0.00000 | 0.08333 | 0.25000 | 0.30000 | 0.20000 |
| 22-022 | 0.75000 | 0.66667 | 0.58333 | 0.58333 | 0.50000 | 0.00000 | 0.16667 | 0.10000 | 0.00000 | 0.00000 | 0.11111 | 0.16667 | 0.12500 | 0.10000 | 0.20000 |
| 22-023 | 0.50000 | 0.66667 | 0.41667 | 0.75000 | 0.50000 | 0.00000 | 0.08333 | 0.00000 | 0.00000 | 0.08333 | 0.00000 | 0.00000 | 0.00000 | 0.20000 | 0.40000 |
| 22-024 | 0.25000 | 0.33333 | 0.16667 | 0.25000 | 0.00000 | 0.00000 | 0.00000 | 0.00000 | 0.11111 | 0.25000 | 0.00000 | 0.00000 | 0.25000 | 0.00000 | 0.20000 |
| 22-025 | 0.50000 | 0.58333 | 0.66667 | 0.58333 | 0.41667 | 0.11111 | 0.25000 | 0.00000 | 0.00000 | 0.25000 | 0.11111 | 0.16667 | 0.12500 | 0.20000 | 0.60000 |
| 22-026 | 0.25000 | 0.58333 | 0.33333 | 0.50000 | 0.41667 | 0.00000 | 0.00000 | 0.10000 | 0.00000 | 0.00000 | 0.00000 | 0.08333 | 0.00000 | 0.30000 | 0.00000 |
| 22-027 | 0.25000 | 0.41667 | 0.25000 | 0.58333 | 0.00000 | 0.00000 | 0.16667 | 0.00000 | 0.00000 | 0.25000 | 0.11111 | 0.00000 | 0.12500 | 0.60000 | 0.00000 |
| 22-028 | 0.25000 | 0.00000 | 0.08333 | 0.08333 | 0.00000 | 0.00000 | 0.08333 | 0.00000 | 0.00000 | 0.16667 | 0.11111 | 0.00000 | 0.00000 | 0.20000 | 0.00000 |
| 22-029 | 0.00000 | 0.00000 | 0.08333 | 0.00000 | 0.00000 | 0.00000 | 0.08333 | 0.00000 | 0.00000 | 0.00000 | 0.00000 | 0.00000 | 0.00000 | 0.00000 | 0.00000 |
| 22-030 | 0.00000 | 0.08333 | 0.00000 | 0.08333 | 0.00000 | 0.00000 | 0.00000 | 0.00000 | 0.00000 | 0.00000 | 0.00000 | 0.00000 | 0.00000 | 0.10000 | 0.00000 |
| 22-031 | 0.00000 | 0.00000 | 0.16667 | 0.16667 | 0.00000 | 0.11111 | 0.41667 | 0.30000 | 0.00000 | 0.25000 | 0.22222 | 0.41667 | 0.50000 | 0.10000 | 0.00000 |
| 22-032 | 0.75000 | 0.50000 | 0.41667 | 0.41667 | 0.41667 | 0.00000 | 0.16667 | 0.10000 | 0.00000 | 0.00000 | 0.00000 | 0.08333 | 0.25000 | 0.10000 | 0.00000 |
| 22-033 | 0.25000 | 0.50000 | 0.25000 | 0.25000 | 0.25000 | 0.00000 | 0.16667 | 0.00000 | 0.00000 | 0.00000 | 0.00000 | 0.08333 | 0.12500 | 0.10000 | 0.20000 |
| 22-034 | 0.00000 | 0.25000 | 0.00000 | 0.25000 | 0.08333 | 0.00000 | 0.00000 | 0.00000 | 0.00000 | 0.00000 | 0.00000 | 0.00000 | 0.00000 | 0.10000 | 0.00000 |
| 22-035 | 0.00000 | 0.16667 | 0.08333 | 0.16667 | 0.00000 | 0.00000 | 0.08333 | 0.00000 | 0.00000 | 0.08333 | 0.00000 | 0.16667 | 0.12500 | 0.10000 | 0.00000 |
| 22-036 | 0.00000 | 0.00000 | 0.00000 | 0.00000 | 0.00000 | 0.00000 | 0.00000 | 0.00000 | 0.00000 | 0.00000 | 0.00000 | 0.00000 | 0.00000 | 0.10000 | 0.00000 |
| 22-037 | 0.00000 | 0.08333 | 0.00000 | 0.00000 | 0.00000 | 0.00000 | 0.00000 | 0.00000 | 0.00000 | 0.33333 | 0.00000 | 0.00000 | 0.25000 | 0.00000 | 0.40000 |
| 22-038 | 0.75000 | 0.50000 | 0.33333 | 0.58333 | 0.33333 | 0.00000 | 0.08333 | 0.00000 | 0.00000 | 0.33333 | 0.11111 | 0.00000 | 0.25000 | 0.10000 | 0.40000 |
| 22-039 | 0.25000 | 0.50000 | 0.50000 | 0.41667 | 0.00000 | 0.00000 | 0.16667 | 0.10000 | 0.11111 | 0.16667 | 0.33333 | 0.00000 | 0.00000 | 0.20000 | 0.00000 |
| 22-040 | 0.00000 | 0.25000 | 0.08333 | 0.08333 | 0.00000 | 0.11111 | 0.16667 | 0.60000 | 0.55556 | 0.91667 | 0.66667 | 0.25000 | 0.50000 | 0.50000 | 0.40000 |
| 22-041 | 0.75000 | 0.83333 | 0.58333 | 0.75000 | 0.58333 | 0.11111 | 0.25000 | 0.70000 | 0.22222 | 0.50000 | 0.33333 | 0.16667 | 0.12500 | 0.10000 | 0.40000 |
| 22-042 | 0.75000 | 0.75000 | 0.75000 | 0.75000 | 0.75000 | 0.11111 | 0.08333 | 0.10000 | 0.00000 | 0.16667 | 0.00000 | 0.00000 | 0.00000 | 0.10000 | 0.40000 |
| 22-043 | 0.75000 | 0.75000 | 0.83333 | 0.75000 | 0.41667 | 0.22222 | 0.25000 | 0.20000 | 0.11111 | 0.25000 | 0.00000 | 0.16667 | 0.00000 | 0.20000 | 0.00000 |
| 22-044 | 0.75000 | 0.83333 | 0.83333 | 0.75000 | 0.58333 | 0.22222 | 0.16667 | 0.20000 | 0.11111 | 0.58333 | 0.11111 | 0.16667 | 0.00000 | 0.20000 | 0.60000 |
| 22-045 | 0.50000 | 0.58333 | 0.16667 | 0.58333 | 0.25000 | 0.00000 | 0.25000 | 0.00000 | 0.11111 | 0.66667 | 0.33333 | 0.08333 | 0.12500 | 0.10000 | 0.00000 |
| 22-046 | 0.00000 | 0.08333 | 0.08333 | 0.25000 | 0.08333 | 0.22222 | 0.00000 | 0.00000 | 0.00000 | 0.33333 | 0.11111 | 0.00000 | 0.12500 | 0.00000 | 0.60000 |
| 22-047 | 0.00000 | 0.41667 | 0.50000 | 0.41667 | 0.25000 | 0.11111 | 0.16667 | 0.10000 | 0.00000 | 0.08333 | 0.11111 | 0.00000 | 0.12500 | 0.10000 | 0.60000 |
| 22-048 | 0.50000 | 0.33333 | 0.25000 | 0.08333 | 0.08333 | 0.11111 | 0.00000 | 0.00000 | 0.00000 | 0.25000 | 0.00000 | 0.00000 | 0.12500 | 0.00000 | 0.20000 |
| 22-049 | 0.00000 | 0.08333 | 0.16667 | 0.00000 | 0.00000 | 0.00000 | 0.00000 | 0.00000 | 0.00000 | 0.16667 | 0.00000 | 0.00000 | 0.00000 | 0.00000 | 0.00000 |
| 22-050 | 0.25000 | 0.25000 | 0.25000 | 0.25000 | 0.08333 | 0.00000 | 0.08333 | 0.00000 | 0.00000 | 0.00000 | 0.00000 | 0.00000 | 0.00000 | 0.20000 | 0.00000 |
| 22-051 | 0.25000 | 0.16667 | 0.16667 | 0.25000 | 0.00000 | 0.22222 | 0.58333 | 0.20000 | 0.33333 | 0.58333 | 0.55556 | 1.00000 | 0.75000 | 1.00000 | 0.40000 |
| 22-052 | 0.00000 | 0.00000 | 0.00000 | 0.16667 | 0.00000 | 0.33333 | 0.25000 | 0.00000 | 0.00000 | 0.41667 | 0.11111 | 0.08333 | 0.25000 | 0.30000 | 0.00000 |
| 22-053 | 0.25000 | 0.16667 | 0.16667 | 0.25000 | 0.08333 | 0.22222 | 0.58333 | 0.50000 | 0.22222 | 0.58333 | 0.66667 | 1.00000 | 0.50000 | 0.90000 | 0.40000 |
| 22-054 | 0.00000 | 0.16667 | 0.16667 | 0.25000 | 0.08333 | 0.22222 | 0.66667 | 0.40000 | 0.22222 | 0.58333 | 0.66667 | 1.00000 | 0.75000 | 0.90000 | 0.60000 |
| 22-055 | 0.00000 | 0.08333 | 0.08333 | 0.08333 | 0.16667 | 0.11111 | 0.25000 | 0.00000 | 0.00000 | 0.00000 | 0.00000 | 0.16667 | 0.12500 | 0.10000 | 0.40000 |
| 22-056 | 0.00000 | 0.00000 | 0.00000 | 0.00000 | 0.00000 | 0.00000 | 0.08333 | 0.00000 | 0.00000 | 0.25000 | 0.00000 | 0.00000 | 0.00000 | 0.00000 | 0.00000 |
| 22-057 | 0.00000 | 0.00000 | 0.00000 | 0.00000 | 0.00000 | 0.00000 | 0.00000 | 0.00000 | 0.00000 | 0.25000 | 0.00000 | 0.00000 | 0.00000 | 0.00000 | 0.00000 |
| 22-058 | 0.00000 | 0.08333 | 0.08333 | 0.08333 | 0.00000 | 0.11111 | 0.08333 | 0.00000 | 0.00000 | 0.25000 | 0.00000 | 0.16667 | 0.25000 | 0.20000 | 0.00000 |
| 22-059 | 0.00000 | 0.00000 | 0.00000 | 0.00000 | 0.00000 | 0.00000 | 0.16667 | 0.00000 | 0.00000 | 0.25000 | 0.00000 | 0.00000 | 0.00000 | 0.00000 | 0.00000 |
| 22-060 | 0.00000 | 0.08333 | 0.16667 | 0.08333 | 0.08333 | 0.11111 | 0.00000 | 0.00000 | 0.00000 | 0.16667 | 0.00000 | 0.50000 | 0.00000 | 0.60000 | 0.40000 |
| 22-061 | 0.00000 | 0.00000 | 0.00000 | 0.00000 | 0.00000 | 0.11111 | 0.16667 | 0.00000 | 0.00000 | 0.25000 | 0.00000 | 0.25000 | 0.00000 | 0.40000 | 0.00000 |
| 22-062 | 0.25000 | 0.08333 | 0.16667 | 0.25000 | 0.00000 | 0.00000 | 0.16667 | 0.40000 | 0.33333 | 0.75000 | 0.77778 | 1.00000 | 0.75000 | 0.60000 | 0.40000 |
| 22-063 | 0.00000 | 0.08333 | 0.00000 | 0.16667 | 0.00000 | 0.11111 | 0.00000 | 0.00000 | 0.11111 | 0.41667 | 0.22222 | 0.41667 | 0.12500 | 0.30000 | 0.20000 |
| 22-064 | 0.00000 | 0.25000 | 0.08333 | 0.33333 | 0.08333 | 0.22222 | 0.16667 | 0.00000 | 0.00000 | 0.41667 | 0.22222 | 0.16667 | 0.12500 | 0.30000 | 0.20000 |
| 22-065 | 0.00000 | 0.00000 | 0.08333 | 0.00000 | 0.08333 | 0.00000 | 0.16667 | 0.00000 | 0.00000 | 0.25000 | 0.22222 | 0.08333 | 0.25000 | 0.30000 | 0.00000 |
| 22-066 | 0.00000 | 0.08333 | 0.08333 | 0.08333 | 0.08333 | 0.11111 | 0.00000 | 0.00000 | 0.11111 | 0.33333 | 0.11111 | 0.00000 | 0.12500 | 0.00000 | 0.20000 |
| 22-067 | 0.00000 | 0.16667 | 0.16667 | 0.33333 | 0.08333 | 0.00000 | 0.00000 | 0.00000 | 0.00000 | 0.33333 | 0.11111 | 0.00000 | 0.12500 | 0.20000 | 0.00000 |
| 22-068 | 0.25000 | 0.08333 | 0.25000 | 0.25000 | 0.00000 | 0.22222 | 0.33333 | 0.20000 | 0.00000 | 0.50000 | 0.00000 | 0.58333 | 0.37500 | 0.20000 | 0.20000 |
| 22-069 | 0.25000 | 0.25000 | 0.33333 | 0.50000 | 0.08333 | 0.00000 | 0.25000 | 0.40000 | 0.00000 | 0.50000 | 0.00000 | 0.83333 | 0.12500 | 0.90000 | 0.00000 |
| 22-070 | 0.25000 | 0.25000 | 0.16667 | 0.25000 | 0.16667 | 0.22222 | 0.25000 | 0.40000 | 0.11111 | 0.50000 | 0.44444 | 1.00000 | 0.37500 | 0.90000 | 0.40000 |
| 22-071 | 0.00000 | 0.25000 | 0.00000 | 0.16667 | 0.00000 | 0.11111 | 0.08333 | 0.00000 | 0.11111 | 0.08333 | 0.00000 | 0.25000 | 0.00000 | 0.60000 | 0.20000 |
| 22-072 | 0.00000 | 0.00000 | 0.08333 | 0.16667 | 0.00000 | 0.00000 | 0.08333 | 0.00000 | 0.00000 | 0.00000 | 0.22222 | 0.16667 | 0.00000 | 0.00000 | 0.00000 |
| 22-073 | 0.50000 | 0.08333 | 0.00000 | 0.08333 | 0.08333 | 0.22222 | 0.16667 | 0.00000 | 0.00000 | 0.50000 | 0.11111 | 0.00000 | 0.37500 | 0.00000 | 0.40000 |
| 22-074 | 0.00000 | 0.00000 | 0.00000 | 0.08333 | 0.00000 | 0.11111 | 0.00000 | 0.00000 | 0.00000 | 0.41667 | 0.11111 | 0.08333 | 0.37500 | 0.10000 | 0.60000 |
| 22-075 | 0.00000 | 0.08333 | 0.00000 | 0.08333 | 0.00000 | 0.00000 | 0.16667 | 0.00000 | 0.00000 | 0.41667 | 0.00000 | 0.00000 | 0.12500 | 0.00000 | 0.20000 |
| 22-076 | 0.00000 | 0.08333 | 0.00000 | 0.16667 | 0.00000 | 0.33333 | 0.41667 | 0.10000 | 0.11111 | 0.41667 | 0.00000 | 0.91667 | 0.50000 | 0.50000 | 0.40000 |
| 22-077 | 0.75000 | 0.08333 | 0.00000 | 0.16667 | 0.00000 | 0.22222 | 0.16667 | 0.00000 | 0.11111 | 0.66667 | 0.00000 | 0.91667 | 0.37500 | 0.20000 | 0.40000 |
| 22-078 | 0.25000 | 0.08333 | 0.16667 | 0.16667 | 0.00000 | 0.11111 | 0.41667 | 0.10000 | 0.44444 | 0.50000 | 0.44444 | 0.83333 | 0.50000 | 0.70000 | 0.00000 |
| 22-079 | 0.25000 | 0.08333 | 0.16667 | 0.16667 | 0.00000 | 0.11111 | 0.41667 | 0.10000 | 0.11111 | 0.83333 | 0.44444 | 0.83333 | 0.12500 | 0.60000 | 0.00000 |
| 22-080 | 0.00000 | 0.25000 | 0.00000 | 0.00000 | 0.08333 | 0.22222 | 0.33333 | 0.00000 | 0.00000 | 0.08333 | 0.00000 | 0.00000 | 0.00000 | 0.00000 | 0.00000 |
| 22-081 | 0.00000 | 0.00000 | 0.08333 | 0.00000 | 0.00000 | 0.11111 | 0.16667 | 0.00000 | 0.00000 | 0.25000 | 0.11111 | 0.16667 | 0.00000 | 0.00000 | 0.20000 |
| 22-082 | 0.00000 | 0.08333 | 0.16667 | 0.08333 | 0.00000 | 0.11111 | 0.50000 | 0.20000 | 0.11111 | 0.75000 | 0.55556 | 0.25000 | 0.25000 | 0.50000 | 0.00000 |
| 22-083 | 0.00000 | 0.08333 | 0.08333 | 0.08333 | 0.08333 | 0.22222 | 0.08333 | 0.60000 | 0.22222 | 0.91667 | 0.55556 | 0.08333 | 0.87500 | 0.20000 | 0.20000 |
| 22-084 | 0.00000 | 0.08333 | 0.00000 | 0.00000 | 0.00000 | 0.00000 | 0.08333 | 0.00000 | 0.00000 | 0.08333 | 0.00000 | 0.08333 | 0.12500 | 0.10000 | 0.00000 |
| 22-085 | 0.00000 | 0.08333 | 0.00000 | 0.00000 | 0.00000 | 0.00000 | 0.16667 | 0.00000 | 0.00000 | 0.41667 | 0.00000 | 0.00000 | 0.25000 | 0.00000 | 0.20000 |
| 22-086 | 0.00000 | 0.08333 | 0.00000 | 0.00000 | 0.00000 | 0.00000 | 0.00000 | 0.00000 | 0.00000 | 0.08333 | 0.00000 | 0.00000 | 0.00000 | 0.00000 | 0.00000 |
| 22-087 | 0.00000 | 0.08333 | 0.00000 | 0.00000 | 0.00000 | 0.00000 | 0.00000 | 0.00000 | 0.00000 | 0.16667 | 0.00000 | 0.00000 | 0.00000 | 0.00000 | 0.00000 |
| 22-088 | 0.00000 | 0.08333 | 0.00000 | 0.00000 | 0.00000 | 0.11111 | 0.08333 | 0.00000 | 0.00000 | 0.16667 | 0.00000 | 0.00000 | 0.00000 | 0.00000 | 0.00000 |
| 22-089 | 0.00000 | 0.00000 | 0.00000 | 0.00000 | 0.00000 | 0.00000 | 0.00000 | 0.00000 | 0.00000 | 0.16667 | 0.00000 | 0.00000 | 0.00000 | 0.00000 | 0.20000 |
| 22-090 | 0.00000 | 0.08333 | 0.00000 | 0.00000 | 0.00000 | 0.22222 | 0.00000 | 0.00000 | 0.00000 | 0.33333 | 0.00000 | 0.00000 | 0.25000 | 0.00000 | 0.40000 |
| 22-091 | 0.00000 | 0.00000 | 0.00000 | 0.00000 | 0.00000 | 0.00000 | 0.00000 | 0.00000 | 0.00000 | 0.33333 | 0.00000 | 0.00000 | 0.00000 | 0.00000 | 0.00000 |
| 22-092 | 0.00000 | 0.08333 | 0.00000 | 0.00000 | 0.00000 | 0.00000 | 0.08333 | 0.00000 | 0.00000 | 0.25000 | 0.00000 | 0.00000 | 0.00000 | 0.00000 | 0.20000 |
| 22-093 | 0.00000 | 0.00000 | 0.00000 | 0.00000 | 0.00000 | 0.00000 | 0.00000 | 0.00000 | 0.00000 | 0.41667 | 0.00000 | 0.00000 | 0.12500 | 0.00000 | 0.00000 |
| 22-094 | 0.00000 | 0.08333 | 0.00000 | 0.08333 | 0.00000 | 0.11111 | 0.00000 | 0.00000 | 0.22222 | 0.16667 | 0.44444 | 0.41667 | 0.12500 | 0.00000 | 0.00000 |
| 22-095 | 0.00000 | 0.16667 | 0.00000 | 0.00000 | 0.00000 | 0.00000 | 0.25000 | 0.20000 | 0.11111 | 0.41667 | 0.22222 | 0.16667 | 0.37500 | 0.00000 | 0.00000 |
| 22-096 | 0.00000 | 0.16667 | 0.00000 | 0.00000 | 0.00000 | 0.22222 | 0.16667 | 0.00000 | 0.00000 | 0.33333 | 0.00000 | 0.00000 | 0.25000 | 0.00000 | 0.00000 |
| 22-097 | 0.00000 | 0.25000 | 0.00000 | 0.08333 | 0.08333 | 0.11111 | 0.16667 | 0.00000 | 0.22222 | 0.41667 | 0.11111 | 0.00000 | 0.25000 | 0.00000 | 0.20000 |
| 22-098 | 0.00000 | 0.16667 | 0.00000 | 0.08333 | 0.00000 | 0.22222 | 0.16667 | 0.00000 | 0.00000 | 0.25000 | 0.00000 | 0.00000 | 0.00000 | 0.00000 | 0.20000 |
| 22-099 | 0.00000 | 0.16667 | 0.00000 | 0.00000 | 0.00000 | 0.11111 | 0.08333 | 0.00000 | 0.00000 | 0.25000 | 0.00000 | 0.00000 | 0.00000 | 0.00000 | 0.00000 |
| 22-100 | 0.00000 | 0.00000 | 0.00000 | 0.00000 | 0.00000 | 0.11111 | 0.00000 | 0.00000 | 0.00000 | 0.41667 | 0.00000 | 0.00000 | 0.25000 | 0.00000 | 0.00000 |
| 22-101 | 0.00000 | 0.08333 | 0.00000 | 0.00000 | 0.00000 | 0.00000 | 0.08333 | 0.00000 | 0.00000 | 0.41667 | 0.00000 | 0.00000 | 0.00000 | 0.00000 | 0.00000 |
| 22-102 | 0.00000 | 0.08333 | 0.00000 | 0.08333 | 0.00000 | 0.11111 | 0.00000 | 0.00000 | 0.00000 | 0.33333 | 0.00000 | 0.00000 | 0.12500 | 0.10000 | 0.00000 |
| 22-103 | 0.00000 | 0.00000 | 0.00000 | 0.00000 | 0.00000 | 0.11111 | 0.00000 | 0.00000 | 0.00000 | 0.16667 | 0.00000 | 0.08333 | 0.12500 | 0.00000 | 0.00000 |
| 22-104 | 0.00000 | 0.00000 | 0.00000 | 0.00000 | 0.00000 | 0.00000 | 0.00000 | 0.00000 | 0.00000 | 0.16667 | 0.00000 | 0.16667 | 0.00000 | 0.00000 | 0.00000 |
| 22-105 | 0.00000 | 0.00000 | 0.16667 | 0.16667 | 0.16667 | 0.11111 | 0.50000 | 0.20000 | 0.22222 | 0.66667 | 0.66667 | 0.83333 | 0.50000 | 0.90000 | 0.00000 |
| 22-106 | 0.25000 | 0.33333 | 0.25000 | 0.25000 | 0.50000 | 0.33333 | 0.58333 | 0.90000 | 0.88889 | 0.58333 | 0.88889 | 1.00000 | 0.75000 | 0.80000 | 0.20000 |
| 22-107 | 0.00000 | 0.08333 | 0.16667 | 0.16667 | 0.16667 | 0.44444 | 0.41667 | 0.80000 | 0.66667 | 0.75000 | 0.88889 | 0.91667 | 0.87500 | 0.80000 | 0.20000 |
| 22-108 | 0.00000 | 0.00000 | 0.25000 | 0.16667 | 0.25000 | 0.33333 | 0.58333 | 0.90000 | 0.44444 | 0.75000 | 0.88889 | 1.00000 | 0.87500 | 0.90000 | 0.20000 |
| 22-109 | 0.00000 | 0.00000 | 0.00000 | 0.00000 | 0.00000 | 0.11111 | 0.25000 | 0.20000 | 0.00000 | 0.16667 | 0.22222 | 0.66667 | 0.12500 | 0.50000 | 0.00000 |
| 22-110 | 0.00000 | 0.00000 | 0.00000 | 0.00000 | 0.00000 | 0.00000 | 0.00000 | 0.00000 | 0.00000 | 0.00000 | 0.00000 | 0.08333 | 0.00000 | 0.20000 | 0.00000 |
| 22-111 | 0.00000 | 0.00000 | 0.00000 | 0.08333 | 0.00000 | 0.11111 | 0.16667 | 0.10000 | 0.11111 | 0.25000 | 0.22222 | 0.00000 | 0.12500 | 0.00000 | 0.00000 |
| 22-112 | 0.00000 | 0.08333 | 0.16667 | 0.08333 | 0.00000 | 0.22222 | 0.00000 | 0.00000 | 0.11111 | 0.33333 | 0.00000 | 0.00000 | 0.12500 | 0.00000 | 0.00000 |
| 22-113 | 0.00000 | 0.00000 | 0.00000 | 0.00000 | 0.00000 | 0.22222 | 0.00000 | 0.00000 | 0.11111 | 0.00000 | 0.00000 | 0.00000 | 0.00000 | 0.00000 | 0.00000 |
| 22-114 | 0.00000 | 0.00000 | 0.00000 | 0.00000 | 0.00000 | 0.11111 | 0.00000 | 0.10000 | 0.11111 | 0.08333 | 0.00000 | 0.00000 | 0.12500 | 0.00000 | 0.00000 |
| 22-115 | 0.00000 | 0.00000 | 0.00000 | 0.00000 | 0.00000 | 0.00000 | 0.00000 | 0.00000 | 0.00000 | 0.00000 | 0.00000 | 0.00000 | 0.00000 | 0.00000 | 0.00000 |
| 22-116 | 0.00000 | 0.00000 | 0.00000 | 0.00000 | 0.00000 | 0.00000 | 0.08333 | 0.30000 | 0.11111 | 0.08333 | 0.00000 | 0.08333 | 0.25000 | 0.00000 | 0.00000 |
| 22-117 | 0.00000 | 0.08333 | 0.00000 | 0.00000 | 0.00000 | 0.22222 | 0.25000 | 0.00000 | 0.00000 | 0.00000 | 0.00000 | 0.00000 | 0.25000 | 0.00000 | 0.20000 |
| 22-118 | 0.00000 | 0.16667 | 0.00000 | 0.00000 | 0.00000 | 0.33333 | 0.00000 | 0.00000 | 0.00000 | 0.25000 | 0.00000 | 0.00000 | 0.25000 | 0.00000 | 0.20000 |
| 22-119 | 0.00000 | 0.00000 | 0.00000 | 0.00000 | 0.00000 | 0.22222 | 0.00000 | 0.00000 | 0.00000 | 0.25000 | 0.00000 | 0.00000 | 0.12500 | 0.00000 | 0.20000 |
| 22-120 | 0.00000 | 0.00000 | 0.00000 | 0.00000 | 0.00000 | 0.11111 | 0.00000 | 0.00000 | 0.00000 | 0.00000 | 0.00000 | 0.00000 | 0.00000 | 0.00000 | 0.00000 |
| 22-121 | 0.00000 | 0.00000 | 0.00000 | 0.00000 | 0.00000 | 0.00000 | 0.00000 | 0.00000 | 0.00000 | 0.08333 | 0.00000 | 0.00000 | 0.00000 | 0.00000 | 0.00000 |
| 22-122 | 0.00000 | 0.08333 | 0.00000 | 0.00000 | 0.00000 | 0.11111 | 0.00000 | 0.00000 | 0.00000 | 0.00000 | 0.00000 | 0.00000 | 0.00000 | 0.00000 | 0.00000 |
| 22-123 | 0.00000 | 0.08333 | 0.08333 | 0.00000 | 0.00000 | 0.00000 | 0.00000 | 0.30000 | 0.00000 | 0.00000 | 0.22222 | 0.00000 | 0.00000 | 0.00000 | 0.00000 |
| 22-124 | 0.00000 | 0.00000 | 0.00000 | 0.00000 | 0.00000 | 0.00000 | 0.00000 | 0.00000 | 0.11111 | 0.00000 | 0.11111 | 0.00000 | 0.12500 | 0.00000 | 0.00000 |
| 22-125 | 0.00000 | 0.00000 | 0.00000 | 0.00000 | 0.00000 | 0.00000 | 0.00000 | 0.00000 | 0.00000 | 0.00000 | 0.00000 | 0.00000 | 0.00000 | 0.00000 | 0.00000 |
| 22-126 | 0.00000 | 0.00000 | 0.00000 | 0.00000 | 0.00000 | 0.00000 | 0.00000 | 0.10000 | 0.00000 | 0.00000 | 0.00000 | 0.00000 | 0.00000 | 0.00000 | 0.00000 |
| 22-127 | 0.00000 | 0.00000 | 0.00000 | 0.00000 | 0.00000 | 0.00000 | 0.00000 | 0.00000 | 0.11111 | 0.00000 | 0.33333 | 0.08333 | 0.12500 | 0.00000 | 0.00000 |
| 22-128 | 0.00000 | 0.00000 | 0.00000 | 0.00000 | 0.00000 | 0.00000 | 0.00000 | 0.00000 | 0.00000 | 0.00000 | 0.11111 | 0.08333 | 0.12500 | 0.00000 | 0.00000 |
| 22-129 | 0.00000 | 0.08333 | 0.00000 | 0.00000 | 0.08333 | 0.00000 | 0.08333 | 0.00000 | 0.11111 | 0.00000 | 0.00000 | 0.00000 | 0.00000 | 0.00000 | 0.00000 |
| 22-130 | 0.00000 | 0.00000 | 0.00000 | 0.00000 | 0.00000 | 0.00000 | 0.00000 | 0.00000 | 0.00000 | 0.00000 | 0.00000 | 0.08333 | 0.00000 | 0.00000 | 0.00000 |
| 22-131 | 0.00000 | 0.00000 | 0.08333 | 0.00000 | 0.00000 | 0.22222 | 0.25000 | 0.00000 | 0.00000 | 0.00000 | 0.00000 | 0.25000 | 0.00000 | 0.00000 | 0.00000 |
| 22-132 | 0.00000 | 0.00000 | 0.00000 | 0.00000 | 0.16667 | 0.00000 | 0.00000 | 0.40000 | 0.00000 | 0.00000 | 0.00000 | 0.00000 | 0.00000 | 0.00000 | 0.00000 |
| 22-133 | 0.00000 | 0.08333 | 0.00000 | 0.00000 | 0.00000 | 0.00000 | 0.25000 | 0.10000 | 0.11111 | 0.00000 | 0.00000 | 0.00000 | 0.00000 | 0.00000 | 0.00000 |
| 22-134 | 0.00000 | 0.00000 | 0.00000 | 0.00000 | 0.00000 | 0.00000 | 0.00000 | 0.00000 | 0.00000 | 0.00000 | 0.00000 | 0.00000 | 0.00000 | 0.00000 | 0.00000 |
| 22-135 | 0.00000 | 0.00000 | 0.00000 | 0.00000 | 0.00000 | 0.00000 | 0.00000 | 0.00000 | 0.00000 | 0.00000 | 0.11111 | 0.00000 | 0.00000 | 0.00000 | 0.00000 |
| 22-136 | 0.00000 | 0.00000 | 0.00000 | 0.00000 | 0.00000 | 0.00000 | 0.00000 | 0.00000 | 0.00000 | 0.08333 | 0.00000 | 0.00000 | 0.00000 | 0.00000 | 0.00000 |
| 22-137 | 0.00000 | 0.08333 | 0.00000 | 0.00000 | 0.00000 | 0.11111 | 0.16667 | 0.00000 | 0.00000 | 0.00000 | 0.00000 | 0.00000 | 0.00000 | 0.00000 | 0.00000 |
| 22-138 | 0.00000 | 0.08333 | 0.00000 | 0.08333 | 0.00000 | 0.00000 | 0.08333 | 0.00000 | 0.00000 | 0.00000 | 0.00000 | 0.00000 | 0.00000 | 0.00000 | 0.00000 |
| 22-139 | 0.00000 | 0.00000 | 0.00000 | 0.00000 | 0.00000 | 0.00000 | 0.08333 | 0.00000 | 0.00000 | 0.00000 | 0.00000 | 0.00000 | 0.00000 | 0.00000 | 0.00000 |
| 22-140 | 0.25000 | 0.00000 | 0.00000 | 0.00000 | 0.00000 | 0.00000 | 0.08333 | 0.00000 | 0.11111 | 0.00000 | 0.00000 | 0.00000 | 0.00000 | 0.00000 | 0.00000 |
| 22-141 | 0.00000 | 0.00000 | 0.00000 | 0.00000 | 0.00000 | 0.00000 | 0.00000 | 0.00000 | 0.11111 | 0.00000 | 0.00000 | 0.00000 | 0.00000 | 0.00000 | 0.00000 |
| 22-142 | 0.25000 | 0.00000 | 0.00000 | 0.00000 | 0.00000 | 0.00000 | 0.00000 | 0.00000 | 0.11111 | 0.00000 | 0.00000 | 0.00000 | 0.00000 | 0.00000 | 0.00000 |
| 22-143 | 0.25000 | 0.00000 | 0.00000 | 0.00000 | 0.08333 | 0.00000 | 0.00000 | 0.00000 | 0.00000 | 0.00000 | 0.00000 | 0.00000 | 0.00000 | 0.00000 | 0.00000 |
| 22-144 | 0.25000 | 0.16667 | 0.00000 | 0.00000 | 0.08333 | 0.00000 | 0.00000 | 0.00000 | 0.00000 | 0.00000 | 0.00000 | 0.00000 | 0.00000 | 0.00000 | 0.00000 |
| 22-145 | 0.00000 | 0.00000 | 0.00000 | 0.00000 | 0.00000 | 0.00000 | 0.00000 | 0.00000 | 0.00000 | 0.00000 | 0.00000 | 0.00000 | 0.00000 | 0.00000 | 0.00000 |
| 22-146 | 0.00000 | 0.00000 | 0.00000 | 0.00000 | 0.00000 | 0.00000 | 0.00000 | 0.00000 | 0.00000 | 0.00000 | 0.00000 | 0.08333 | 0.00000 | 0.00000 | 0.00000 |
| 22-147 | 0.00000 | 0.00000 | 0.00000 | 0.00000 | 0.00000 | 0.00000 | 0.00000 | 0.00000 | 0.00000 | 0.00000 | 0.00000 | 0.00000 | 0.00000 | 0.00000 | 0.00000 |
| 22-148 | 0.00000 | 0.00000 | 0.08333 | 0.00000 | 0.00000 | 0.00000 | 0.16667 | 0.20000 | 0.11111 | 0.00000 | 0.11111 | 0.16667 | 0.00000 | 0.00000 | 0.00000 |
| 22-149 | 0.00000 | 0.00000 | 0.00000 | 0.00000 | 0.00000 | 0.00000 | 0.00000 | 0.00000 | 0.11111 | 0.00000 | 0.00000 | 0.00000 | 0.00000 | 0.00000 | 0.00000 |
| 22-150 | 0.00000 | 0.00000 | 0.00000 | 0.00000 | 0.00000 | 0.11111 | 0.00000 | 0.00000 | 0.00000 | 0.00000 | 0.00000 | 0.00000 | 0.00000 | 0.00000 | 0.00000 |
| 22-151 | 0.00000 | 0.00000 | 0.00000 | 0.00000 | 0.00000 | 0.22222 | 0.00000 | 0.00000 | 0.00000 | 0.00000 | 0.00000 | 0.00000 | 0.00000 | 0.00000 | 0.00000 |
| 22-152 | 0.00000 | 0.00000 | 0.00000 | 0.00000 | 0.00000 | 0.00000 | 0.08333 | 0.00000 | 0.00000 | 0.00000 | 0.00000 | 0.00000 | 0.00000 | 0.00000 | 0.00000 |
| 22-153 | 0.25000 | 0.33333 | 0.08333 | 0.08333 | 0.33333 | 0.11111 | 0.08333 | 0.10000 | 0.00000 | 0.00000 | 0.00000 | 0.00000 | 0.00000 | 0.00000 | 0.00000 |
| 22-154 | 0.00000 | 0.00000 | 0.00000 | 0.00000 | 0.00000 | 0.00000 | 0.08333 | 0.00000 | 0.00000 | 0.00000 | 0.00000 | 0.00000 | 0.00000 | 0.00000 | 0.00000 |
| 22-155 | 0.00000 | 0.00000 | 0.00000 | 0.00000 | 0.00000 | 0.00000 | 0.08333 | 0.00000 | 0.00000 | 0.00000 | 0.00000 | 0.00000 | 0.00000 | 0.00000 | 0.00000 |
| 22-156 | 0.00000 | 0.00000 | 0.00000 | 0.00000 | 0.00000 | 0.00000 | 0.08333 | 0.00000 | 0.00000 | 0.00000 | 0.00000 | 0.00000 | 0.00000 | 0.00000 | 0.00000 |
| 22-157 | 0.00000 | 0.00000 | 0.00000 | 0.00000 | 0.00000 | 0.00000 | 0.08333 | 0.00000 | 0.00000 | 0.00000 | 0.00000 | 0.00000 | 0.00000 | 0.00000 | 0.00000 |
| 30-001 | 0.00000 | 0.08333 | 0.00000 | 0.00000 | 0.00000 | 0.00000 | 0.00000 | 0.00000 | 0.00000 | 0.00000 | 0.00000 | 0.00000 | 0.00000 | 0.00000 | 0.16667 |
| 30-002 | 0.00000 | 0.50000 | 0.08333 | 0.00000 | 0.08333 | 0.00000 | 0.00000 | 0.00000 | 0.00000 | 0.08333 | 0.00000 | 0.00000 | 0.12500 | 0.30000 | 0.33333 |
| 30-003 | 0.00000 | 0.08333 | 0.00000 | 0.00000 | 0.00000 | 0.00000 | 0.00000 | 0.00000 | 0.00000 | 0.00000 | 0.00000 | 0.00000 | 0.00000 | 0.00000 | 0.00000 |
| 30-004 | 0.00000 | 0.00000 | 0.00000 | 0.00000 | 0.00000 | 0.00000 | 0.00000 | 0.00000 | 0.00000 | 0.00000 | 0.00000 | 0.00000 | 0.00000 | 0.00000 | 0.16667 |
| 30-005 | 0.00000 | 0.00000 | 0.08333 | 0.00000 | 0.00000 | 0.00000 | 0.00000 | 0.00000 | 0.00000 | 0.00000 | 0.00000 | 0.00000 | 0.00000 | 0.00000 | 0.00000 |
| 30-006 | 0.00000 | 0.00000 | 0.00000 | 0.00000 | 0.08333 | 0.00000 | 0.00000 | 0.00000 | 0.00000 | 0.00000 | 0.00000 | 0.00000 | 0.00000 | 0.00000 | 0.16667 |
| 30-007 | 0.00000 | 0.00000 | 0.00000 | 0.08333 | 0.00000 | 0.00000 | 0.00000 | 0.00000 | 0.00000 | 0.00000 | 0.00000 | 0.00000 | 0.00000 | 0.00000 | 0.50000 |
| 30-008 | 0.00000 | 0.00000 | 0.00000 | 0.00000 | 0.00000 | 0.00000 | 0.08333 | 0.00000 | 0.00000 | 0.00000 | 0.00000 | 0.00000 | 0.00000 | 0.00000 | 0.16667 |
| 30-009 | 0.60000 | 0.08333 | 0.08333 | 0.16667 | 0.33333 | 0.00000 | 0.16667 | 0.16667 | 0.27273 | 0.16667 | 0.11111 | 0.00000 | 0.00000 | 0.00000 | 0.33333 |
| 30-010 | 0.80000 | 0.33333 | 0.16667 | 0.50000 | 0.41667 | 0.00000 | 0.50000 | 0.00000 | 0.45455 | 0.25000 | 0.22222 | 0.40000 | 0.75000 | 0.90000 | 0.66667 |
| 30-011 | 0.20000 | 0.08333 | 0.00000 | 0.08333 | 0.00000 | 0.09091 | 0.25000 | 0.08333 | 0.00000 | 0.08333 | 0.00000 | 0.00000 | 0.00000 | 0.00000 | 0.00000 |
| 30-012 | 0.00000 | 0.00000 | 0.00000 | 0.08333 | 0.00000 | 0.00000 | 0.00000 | 0.08333 | 0.00000 | 0.00000 | 0.00000 | 0.00000 | 0.00000 | 0.00000 | 0.00000 |
| 30-013 | 0.60000 | 0.58333 | 0.00000 | 0.25000 | 0.25000 | 0.18182 | 0.33333 | 0.33333 | 0.00000 | 0.33333 | 0.00000 | 0.00000 | 0.00000 | 0.10000 | 0.00000 |
| 30-014 | 0.00000 | 0.50000 | 0.75000 | 0.16667 | 0.33333 | 0.18182 | 0.25000 | 0.16667 | 0.00000 | 0.08333 | 0.00000 | 0.00000 | 0.00000 | 0.00000 | 0.00000 |
| 30-015 | 0.00000 | 0.08333 | 0.00000 | 0.00000 | 0.08333 | 0.00000 | 0.08333 | 0.00000 | 0.00000 | 0.00000 | 0.00000 | 0.00000 | 0.00000 | 0.00000 | 0.00000 |
| 30-016 | 0.00000 | 0.00000 | 0.00000 | 0.08333 | 0.00000 | 0.00000 | 0.00000 | 0.00000 | 0.00000 | 0.00000 | 0.00000 | 0.00000 | 0.00000 | 0.00000 | 0.00000 |
| 30-017 | 0.00000 | 0.08333 | 0.08333 | 0.16667 | 0.33333 | 0.00000 | 0.16667 | 0.00000 | 0.00000 | 0.25000 | 0.00000 | 0.10000 | 0.12500 | 0.70000 | 0.66667 |
| 30-018 | 0.00000 | 0.25000 | 0.00000 | 0.00000 | 0.00000 | 0.00000 | 0.00000 | 0.00000 | 0.00000 | 0.00000 | 0.00000 | 0.00000 | 0.00000 | 0.00000 | 0.00000 |
| 30-019 | 0.00000 | 0.00000 | 0.00000 | 0.00000 | 0.00000 | 0.00000 | 0.00000 | 0.00000 | 0.00000 | 0.00000 | 0.00000 | 0.00000 | 0.00000 | 0.10000 | 0.00000 |
| 30-020 | 0.00000 | 0.00000 | 0.00000 | 0.00000 | 0.33333 | 0.18182 | 0.00000 | 0.00000 | 0.00000 | 0.00000 | 0.00000 | 0.00000 | 0.00000 | 0.00000 | 0.00000 |
| 30-021 | 0.00000 | 0.00000 | 0.08333 | 0.00000 | 0.00000 | 0.00000 | 0.00000 | 0.00000 | 0.00000 | 0.00000 | 0.00000 | 0.00000 | 0.00000 | 0.00000 | 0.00000 |
| 30-022 | 0.00000 | 0.00000 | 0.08333 | 0.00000 | 0.00000 | 0.00000 | 0.08333 | 0.00000 | 0.00000 | 0.00000 | 0.00000 | 0.00000 | 0.00000 | 0.00000 | 0.00000 |
| 30-023 | 0.00000 | 0.41667 | 0.25000 | 0.16667 | 0.25000 | 0.00000 | 0.00000 | 0.00000 | 0.00000 | 0.00000 | 0.00000 | 0.00000 | 0.00000 | 0.00000 | 0.00000 |
| 30-024 | 0.00000 | 0.08333 | 0.00000 | 0.00000 | 0.00000 | 0.00000 | 0.00000 | 0.00000 | 0.00000 | 0.00000 | 0.00000 | 0.00000 | 0.00000 | 0.00000 | 0.00000 |
| 30-025 | 0.00000 | 0.00000 | 0.00000 | 0.00000 | 0.00000 | 0.00000 | 0.00000 | 0.00000 | 0.00000 | 0.25000 | 0.00000 | 0.00000 | 0.00000 | 0.10000 | 0.00000 |
| 30-026 | 0.00000 | 0.08333 | 0.08333 | 0.16667 | 0.58333 | 0.18182 | 0.00000 | 0.00000 | 0.00000 | 0.00000 | 0.00000 | 0.10000 | 0.25000 | 0.60000 | 0.16667 |
| 30-027 | 0.00000 | 0.00000 | 0.08333 | 0.00000 | 0.50000 | 0.36364 | 0.00000 | 0.16667 | 0.09091 | 0.08333 | 0.00000 | 0.00000 | 0.12500 | 0.60000 | 0.00000 |
| 30-028 | 0.00000 | 0.00000 | 0.00000 | 0.00000 | 0.08333 | 0.00000 | 0.00000 | 0.00000 | 0.00000 | 0.00000 | 0.00000 | 0.00000 | 0.00000 | 0.00000 | 0.00000 |
| 30-029 | 0.00000 | 0.00000 | 0.00000 | 0.00000 | 0.08333 | 0.00000 | 0.00000 | 0.00000 | 0.00000 | 0.00000 | 0.00000 | 0.00000 | 0.00000 | 0.00000 | 0.00000 |
| 30-030 | 0.00000 | 0.00000 | 0.08333 | 0.08333 | 0.00000 | 0.00000 | 0.00000 | 0.00000 | 0.00000 | 0.00000 | 0.00000 | 0.00000 | 0.00000 | 0.00000 | 0.00000 |
| 30-031 | 0.00000 | 0.00000 | 0.00000 | 0.00000 | 0.00000 | 0.00000 | 0.00000 | 0.00000 | 0.00000 | 0.00000 | 0.00000 | 0.10000 | 0.00000 | 0.00000 | 0.00000 |
| 30-032 | 0.00000 | 0.08333 | 0.08333 | 0.00000 | 0.08333 | 0.00000 | 0.00000 | 0.00000 | 0.00000 | 0.25000 | 0.22222 | 0.00000 | 0.25000 | 0.10000 | 0.00000 |
| 30-033 | 0.00000 | 0.00000 | 0.00000 | 0.00000 | 0.00000 | 0.09091 | 0.00000 | 0.00000 | 0.00000 | 0.00000 | 0.00000 | 0.00000 | 0.00000 | 0.00000 | 0.00000 |
| 30-034 | 0.00000 | 0.00000 | 0.00000 | 0.00000 | 0.00000 | 0.00000 | 0.00000 | 0.00000 | 0.00000 | 0.00000 | 0.00000 | 0.00000 | 0.00000 | 0.10000 | 0.00000 |
| 30-035 | 0.00000 | 0.00000 | 0.00000 | 0.00000 | 0.00000 | 0.00000 | 0.00000 | 0.08333 | 0.00000 | 0.00000 | 0.00000 | 0.00000 | 0.00000 | 0.00000 | 0.00000 |
| 30-036 | 0.00000 | 0.00000 | 0.00000 | 0.08333 | 0.00000 | 0.09091 | 0.00000 | 0.08333 | 0.00000 | 0.00000 | 0.00000 | 0.00000 | 0.00000 | 0.00000 | 0.16667 |
| 30-037 | 0.00000 | 0.00000 | 0.00000 | 0.16667 | 0.00000 | 0.09091 | 0.00000 | 0.16667 | 0.00000 | 0.16667 | 0.00000 | 0.00000 | 0.00000 | 0.00000 | 0.16667 |
| 30-038 | 0.00000 | 0.00000 | 0.08333 | 0.00000 | 0.00000 | 0.00000 | 0.00000 | 0.00000 | 0.00000 | 0.16667 | 0.11111 | 0.10000 | 0.00000 | 0.00000 | 0.00000 |
| 30-039 | 0.00000 | 0.00000 | 0.00000 | 0.00000 | 0.00000 | 0.00000 | 0.00000 | 0.00000 | 0.00000 | 0.00000 | 0.11111 | 0.00000 | 0.37500 | 0.00000 | 0.00000 |
| 30-040 | 0.20000 | 0.25000 | 0.08333 | 0.08333 | 0.08333 | 0.09091 | 0.33333 | 0.08333 | 0.00000 | 0.00000 | 0.00000 | 0.00000 | 0.00000 | 0.00000 | 0.00000 |
| 30-041 | 0.00000 | 0.16667 | 0.00000 | 0.25000 | 0.08333 | 0.18182 | 0.58333 | 0.41667 | 0.00000 | 0.33333 | 0.00000 | 0.00000 | 0.12500 | 0.10000 | 0.00000 |
| 30-042 | 0.00000 | 0.08333 | 0.00000 | 0.00000 | 0.00000 | 0.00000 | 0.00000 | 0.00000 | 0.00000 | 0.00000 | 0.00000 | 0.00000 | 0.00000 | 0.00000 | 0.00000 |
| 30-043 | 0.00000 | 0.00000 | 0.00000 | 0.00000 | 0.08333 | 0.00000 | 0.00000 | 0.00000 | 0.00000 | 0.00000 | 0.00000 | 0.00000 | 0.00000 | 0.00000 | 0.00000 |
| 30-044 | 0.00000 | 0.00000 | 0.08333 | 0.00000 | 0.16667 | 0.00000 | 0.00000 | 0.00000 | 0.00000 | 0.00000 | 0.00000 | 0.10000 | 0.00000 | 0.30000 | 0.00000 |
| 30-045 | 0.60000 | 0.66667 | 0.25000 | 0.41667 | 0.66667 | 0.72727 | 0.25000 | 0.83333 | 0.90909 | 0.16667 | 0.22222 | 0.40000 | 0.37500 | 0.40000 | 0.50000 |
| 30-046 | 0.00000 | 0.00000 | 0.41667 | 0.08333 | 0.33333 | 0.18182 | 0.08333 | 0.00000 | 0.27273 | 0.41667 | 0.88889 | 0.60000 | 1.00000 | 0.20000 | 0.50000 |
| 30-047 | 0.80000 | 0.91667 | 0.83333 | 0.66667 | 0.91667 | 0.81818 | 0.91667 | 0.91667 | 1.00000 | 1.00000 | 0.88889 | 1.00000 | 1.00000 | 1.00000 | 0.66667 |
| 30-048 | 0.00000 | 0.00000 | 0.00000 | 0.08333 | 0.00000 | 0.00000 | 0.00000 | 0.00000 | 0.00000 | 0.00000 | 0.00000 | 0.00000 | 0.00000 | 0.00000 | 0.00000 |
| 30-049 | 0.20000 | 0.08333 | 0.00000 | 0.16667 | 0.08333 | 0.00000 | 0.16667 | 0.00000 | 0.00000 | 0.08333 | 0.00000 | 0.00000 | 0.00000 | 0.00000 | 0.00000 |
| 30-050 | 0.00000 | 0.08333 | 0.00000 | 0.00000 | 0.08333 | 0.00000 | 0.08333 | 0.00000 | 0.00000 | 0.00000 | 0.00000 | 0.00000 | 0.00000 | 0.00000 | 0.00000 |
| 30-051 | 0.00000 | 0.00000 | 0.00000 | 0.25000 | 0.00000 | 0.09091 | 0.08333 | 0.00000 | 0.00000 | 0.00000 | 0.00000 | 0.00000 | 0.00000 | 0.00000 | 0.00000 |
| 30-052 | 0.00000 | 0.00000 | 0.08333 | 0.16667 | 0.08333 | 0.09091 | 0.16667 | 0.00000 | 0.00000 | 0.00000 | 0.00000 | 0.00000 | 0.00000 | 0.00000 | 0.00000 |
| 30-053 | 0.00000 | 0.16667 | 0.08333 | 0.25000 | 0.08333 | 0.09091 | 0.08333 | 0.25000 | 0.00000 | 0.50000 | 0.11111 | 0.00000 | 0.12500 | 0.00000 | 0.16667 |
| 30-054 | 0.00000 | 0.00000 | 0.08333 | 0.00000 | 0.41667 | 0.00000 | 0.00000 | 0.00000 | 0.00000 | 0.00000 | 0.11111 | 0.20000 | 0.12500 | 0.00000 | 0.16667 |
| 30-055 | 0.00000 | 0.00000 | 0.00000 | 0.08333 | 0.00000 | 0.00000 | 0.00000 | 0.16667 | 0.00000 | 0.08333 | 0.00000 | 0.00000 | 0.00000 | 0.00000 | 0.00000 |
| 30-056 | 0.00000 | 0.00000 | 0.00000 | 0.00000 | 0.00000 | 0.00000 | 0.00000 | 0.00000 | 0.09091 | 0.00000 | 0.00000 | 0.00000 | 0.00000 | 0.20000 | 0.00000 |
| 30-057 | 0.00000 | 0.00000 | 0.00000 | 0.00000 | 0.00000 | 0.00000 | 0.00000 | 0.00000 | 0.09091 | 0.00000 | 0.00000 | 0.00000 | 0.00000 | 0.30000 | 0.00000 |
| 30-058 | 0.00000 | 0.00000 | 0.00000 | 0.00000 | 0.08333 | 0.00000 | 0.00000 | 0.00000 | 0.00000 | 0.08333 | 0.00000 | 0.00000 | 0.00000 | 0.00000 | 0.00000 |
| 30-059 | 0.00000 | 0.00000 | 0.00000 | 0.08333 | 0.00000 | 0.00000 | 0.00000 | 0.00000 | 0.00000 | 0.00000 | 0.00000 | 0.00000 | 0.00000 | 0.00000 | 0.00000 |
| 30-060 | 0.00000 | 0.00000 | 0.00000 | 0.08333 | 0.00000 | 0.00000 | 0.00000 | 0.00000 | 0.00000 | 0.00000 | 0.00000 | 0.00000 | 0.00000 | 0.00000 | 0.00000 |
| 30-061 | 0.00000 | 0.00000 | 0.08333 | 0.00000 | 0.00000 | 0.00000 | 0.00000 | 0.00000 | 0.00000 | 0.00000 | 0.00000 | 0.00000 | 0.00000 | 0.00000 | 0.00000 |
| 30-062 | 0.00000 | 0.00000 | 0.08333 | 0.00000 | 0.00000 | 0.00000 | 0.00000 | 0.00000 | 0.00000 | 0.00000 | 0.00000 | 0.00000 | 0.00000 | 0.00000 | 0.00000 |
| 30-063 | 0.00000 | 0.00000 | 0.00000 | 0.00000 | 0.00000 | 0.00000 | 0.00000 | 0.00000 | 0.00000 | 0.00000 | 0.00000 | 0.00000 | 0.00000 | 0.10000 | 0.00000 |
| 30-064 | 0.00000 | 0.00000 | 0.00000 | 0.00000 | 0.00000 | 0.00000 | 0.00000 | 0.00000 | 0.00000 | 0.00000 | 0.00000 | 0.00000 | 0.00000 | 0.10000 | 0.00000 |
| 30-065 | 0.00000 | 0.00000 | 0.00000 | 0.00000 | 0.00000 | 0.00000 | 0.00000 | 0.00000 | 0.00000 | 0.08333 | 0.00000 | 0.00000 | 0.00000 | 0.00000 | 0.00000 |
| 30-066 | 0.00000 | 0.08333 | 0.00000 | 0.08333 | 0.00000 | 0.00000 | 0.00000 | 0.00000 | 0.09091 | 0.08333 | 0.00000 | 0.00000 | 0.00000 | 0.00000 | 0.00000 |
| 30-067 | 0.00000 | 0.08333 | 0.00000 | 0.08333 | 0.00000 | 0.00000 | 0.00000 | 0.00000 | 0.00000 | 0.08333 | 0.22222 | 0.00000 | 0.00000 | 0.10000 | 0.00000 |
| 30-068 | 0.00000 | 0.16667 | 0.00000 | 0.41667 | 0.08333 | 0.09091 | 0.00000 | 0.00000 | 0.00000 | 0.25000 | 0.00000 | 0.10000 | 0.00000 | 0.20000 | 0.00000 |
| 30-069 | 0.00000 | 0.08333 | 0.00000 | 0.16667 | 0.00000 | 0.09091 | 0.08333 | 0.16667 | 0.00000 | 0.16667 | 0.00000 | 0.00000 | 0.00000 | 0.00000 | 0.00000 |
| 30-070 | 0.20000 | 0.25000 | 0.08333 | 0.16667 | 0.00000 | 0.00000 | 0.00000 | 0.08333 | 0.00000 | 0.16667 | 0.00000 | 0.10000 | 0.00000 | 0.10000 | 0.00000 |
| 30-071 | 0.20000 | 0.16667 | 0.08333 | 0.41667 | 0.08333 | 0.18182 | 0.00000 | 0.00000 | 0.00000 | 0.25000 | 0.00000 | 0.00000 | 0.00000 | 0.00000 | 0.00000 |
| 30-072 | 0.00000 | 0.16667 | 0.00000 | 0.08333 | 0.00000 | 0.09091 | 0.00000 | 0.00000 | 0.00000 | 0.00000 | 0.00000 | 0.10000 | 0.12500 | 0.00000 | 0.00000 |
| 30-073 | 0.00000 | 0.25000 | 0.16667 | 0.16667 | 0.16667 | 0.36364 | 0.08333 | 0.16667 | 0.09091 | 0.25000 | 0.22222 | 0.00000 | 0.12500 | 0.20000 | 0.00000 |
| 30-074 | 0.00000 | 0.08333 | 0.08333 | 0.08333 | 0.16667 | 0.00000 | 0.58333 | 0.00000 | 0.00000 | 0.08333 | 0.00000 | 0.00000 | 0.00000 | 0.10000 | 0.00000 |
| 30-075 | 0.00000 | 0.16667 | 0.00000 | 0.00000 | 0.25000 | 0.09091 | 0.08333 | 0.00000 | 0.00000 | 0.00000 | 0.00000 | 0.10000 | 0.00000 | 0.20000 | 0.00000 |
| 30-076 | 0.00000 | 0.00000 | 0.00000 | 0.08333 | 0.08333 | 0.00000 | 0.08333 | 0.00000 | 0.00000 | 0.00000 | 0.00000 | 0.00000 | 0.00000 | 0.00000 | 0.00000 |
| 30-077 | 0.20000 | 0.08333 | 0.16667 | 0.16667 | 0.16667 | 0.09091 | 0.08333 | 0.16667 | 0.09091 | 0.00000 | 0.22222 | 0.10000 | 0.12500 | 0.10000 | 0.00000 |
| 30-078 | 0.20000 | 0.08333 | 0.08333 | 0.00000 | 0.41667 | 0.00000 | 0.08333 | 0.08333 | 0.00000 | 0.00000 | 0.11111 | 0.00000 | 0.00000 | 0.10000 | 0.00000 |
| 30-079 | 0.00000 | 0.16667 | 0.16667 | 0.08333 | 0.41667 | 0.18182 | 0.33333 | 0.00000 | 0.00000 | 0.08333 | 0.11111 | 0.00000 | 0.00000 | 0.10000 | 0.00000 |
| 30-080 | 0.20000 | 0.41667 | 0.25000 | 0.16667 | 0.16667 | 0.09091 | 0.16667 | 0.41667 | 0.36364 | 0.16667 | 0.22222 | 0.10000 | 0.12500 | 0.20000 | 0.00000 |
| 30-081 | 0.00000 | 0.00000 | 0.00000 | 0.16667 | 0.00000 | 0.09091 | 0.00000 | 0.00000 | 0.09091 | 0.08333 | 0.22222 | 0.00000 | 0.00000 | 0.00000 | 0.00000 |
| 30-082 | 0.00000 | 0.00000 | 0.00000 | 0.00000 | 0.00000 | 0.00000 | 0.00000 | 0.00000 | 0.09091 | 0.00000 | 0.00000 | 0.00000 | 0.00000 | 0.00000 | 0.00000 |
| 30-083 | 0.00000 | 0.00000 | 0.00000 | 0.00000 | 0.00000 | 0.09091 | 0.00000 | 0.00000 | 0.00000 | 0.00000 | 0.00000 | 0.00000 | 0.00000 | 0.00000 | 0.00000 |
| 30-084 | 0.00000 | 0.00000 | 0.08333 | 0.00000 | 0.08333 | 0.00000 | 0.00000 | 0.00000 | 0.00000 | 0.00000 | 0.00000 | 0.00000 | 0.00000 | 0.00000 | 0.00000 |
| 30-085 | 0.00000 | 0.00000 | 0.00000 | 0.00000 | 0.00000 | 0.00000 | 0.00000 | 0.08333 | 0.09091 | 0.00000 | 0.00000 | 0.00000 | 0.00000 | 0.00000 | 0.00000 |
| 30-086 | 0.00000 | 0.00000 | 0.00000 | 0.00000 | 0.00000 | 0.00000 | 0.00000 | 0.08333 | 0.00000 | 0.00000 | 0.00000 | 0.00000 | 0.00000 | 0.00000 | 0.00000 |
| 30-087 | 0.00000 | 0.00000 | 0.00000 | 0.00000 | 0.08333 | 0.00000 | 0.00000 | 0.00000 | 0.00000 | 0.00000 | 0.00000 | 0.00000 | 0.00000 | 0.00000 | 0.00000 |
| 30-088 | 0.00000 | 0.00000 | 0.00000 | 0.00000 | 0.25000 | 0.36364 | 0.00000 | 0.16667 | 0.18182 | 0.08333 | 0.22222 | 0.00000 | 0.00000 | 0.00000 | 0.00000 |
| 30-089 | 0.00000 | 0.00000 | 0.00000 | 0.00000 | 0.00000 | 0.00000 | 0.00000 | 0.08333 | 0.00000 | 0.08333 | 0.00000 | 0.00000 | 0.00000 | 0.00000 | 0.00000 |
| 30-090 | 0.00000 | 0.00000 | 0.00000 | 0.00000 | 0.08333 | 0.00000 | 0.00000 | 0.00000 | 0.00000 | 0.00000 | 0.11111 | 0.00000 | 0.25000 | 0.20000 | 0.00000 |
| 30-091 | 0.00000 | 0.00000 | 0.00000 | 0.00000 | 0.08333 | 0.09091 | 0.00000 | 0.08333 | 0.00000 | 0.00000 | 0.00000 | 0.00000 | 0.00000 | 0.00000 | 0.16667 |
| 30-092 | 0.00000 | 0.41667 | 0.00000 | 0.08333 | 0.00000 | 0.09091 | 0.08333 | 0.16667 | 0.00000 | 0.16667 | 0.00000 | 0.00000 | 0.00000 | 0.00000 | 0.00000 |
| 30-093 | 0.00000 | 0.00000 | 0.00000 | 0.00000 | 0.00000 | 0.00000 | 0.00000 | 0.00000 | 0.00000 | 0.00000 | 0.00000 | 0.00000 | 0.00000 | 0.10000 | 0.00000 |
| 30-094 | 0.00000 | 0.00000 | 0.00000 | 0.00000 | 0.00000 | 0.00000 | 0.00000 | 0.00000 | 0.00000 | 0.00000 | 0.00000 | 0.00000 | 0.00000 | 0.10000 | 0.00000 |
| 30-095 | 0.20000 | 0.08333 | 0.00000 | 0.08333 | 0.16667 | 0.00000 | 0.00000 | 0.00000 | 0.00000 | 0.00000 | 0.22222 | 0.00000 | 0.50000 | 0.40000 | 0.00000 |
| 30-096 | 0.00000 | 0.25000 | 0.16667 | 0.16667 | 0.08333 | 0.18182 | 0.58333 | 0.25000 | 0.09091 | 0.25000 | 0.11111 | 0.10000 | 0.00000 | 0.00000 | 0.00000 |
| 30-097 | 0.00000 | 0.08333 | 0.00000 | 0.00000 | 0.00000 | 0.00000 | 0.00000 | 0.00000 | 0.00000 | 0.00000 | 0.00000 | 0.00000 | 0.00000 | 0.00000 | 0.00000 |
| 30-098 | 0.00000 | 0.00000 | 0.00000 | 0.00000 | 0.00000 | 0.00000 | 0.00000 | 0.00000 | 0.00000 | 0.00000 | 0.00000 | 0.00000 | 0.00000 | 0.00000 | 0.16667 |
| 30-099 | 0.00000 | 0.00000 | 0.08333 | 0.00000 | 0.08333 | 0.09091 | 0.00000 | 0.00000 | 0.09091 | 0.00000 | 0.22222 | 0.30000 | 0.00000 | 0.10000 | 0.00000 |
| 30-100 | 0.00000 | 0.00000 | 0.08333 | 0.00000 | 0.00000 | 0.00000 | 0.00000 | 0.00000 | 0.00000 | 0.00000 | 0.00000 | 0.00000 | 0.00000 | 0.00000 | 0.00000 |
| 30-101 | 0.00000 | 0.00000 | 0.00000 | 0.08333 | 0.00000 | 0.09091 | 0.00000 | 0.00000 | 0.00000 | 0.00000 | 0.11111 | 0.00000 | 0.00000 | 0.00000 | 0.00000 |
| 30-102 | 0.00000 | 0.00000 | 0.00000 | 0.00000 | 0.00000 | 0.00000 | 0.00000 | 0.00000 | 0.00000 | 0.00000 | 0.00000 | 0.10000 | 0.00000 | 0.00000 | 0.00000 |
| 30-103 | 0.00000 | 0.00000 | 0.00000 | 0.00000 | 0.00000 | 0.00000 | 0.00000 | 0.00000 | 0.00000 | 0.00000 | 0.00000 | 0.10000 | 0.00000 | 0.00000 | 0.00000 |
| 30-104 | 0.20000 | 0.00000 | 0.00000 | 0.00000 | 0.00000 | 0.00000 | 0.00000 | 0.00000 | 0.00000 | 0.00000 | 0.22222 | 0.00000 | 0.25000 | 0.10000 | 0.00000 |
| 30-105 | 0.00000 | 0.00000 | 0.00000 | 0.00000 | 0.00000 | 0.00000 | 0.00000 | 0.00000 | 0.00000 | 0.00000 | 0.00000 | 0.00000 | 0.00000 | 0.10000 | 0.00000 |
| 30-106 | 0.00000 | 0.00000 | 0.00000 | 0.08333 | 0.00000 | 0.09091 | 0.00000 | 0.00000 | 0.00000 | 0.00000 | 0.11111 | 0.00000 | 0.00000 | 0.00000 | 0.00000 |
| 30-107 | 0.00000 | 0.00000 | 0.00000 | 0.00000 | 0.08333 | 0.00000 | 0.00000 | 0.00000 | 0.00000 | 0.00000 | 0.00000 | 0.00000 | 0.00000 | 0.00000 | 0.00000 |
| 30-108 | 0.00000 | 0.00000 | 0.00000 | 0.00000 | 0.00000 | 0.00000 | 0.00000 | 0.00000 | 0.00000 | 0.00000 | 0.00000 | 0.00000 | 0.00000 | 0.00000 | 0.16667 |
| 30-109 | 0.00000 | 0.00000 | 0.00000 | 0.00000 | 0.00000 | 0.00000 | 0.00000 | 0.00000 | 0.00000 | 0.00000 | 0.00000 | 0.00000 | 0.00000 | 0.00000 | 0.16667 |
| 30-110 | 0.00000 | 0.00000 | 0.00000 | 0.00000 | 0.00000 | 0.00000 | 0.00000 | 0.00000 | 0.00000 | 0.00000 | 0.00000 | 0.00000 | 0.00000 | 0.00000 | 0.16667 |
| 30-111 | 0.20000 | 0.00000 | 0.00000 | 0.00000 | 0.00000 | 0.00000 | 0.00000 | 0.00000 | 0.00000 | 0.00000 | 0.00000 | 0.00000 | 0.00000 | 0.00000 | 0.00000 |
| 31-001 | 0.00000 | 0.00000 | 0.09091 | 0.00000 | 0.00000 | 0.00000 | 0.00000 | 0.00000 | 0.00000 | 0.00000 | 0.00000 | 0.00000 | 0.00000 | 0.00000 | 0.16667 |
| 31-002 | 0.00000 | 0.00000 | 0.00000 | 0.00000 | 0.00000 | 0.00000 | 0.00000 | 0.00000 | 0.00000 | 0.08333 | 0.00000 | 0.00000 | 0.12500 | 0.30000 | 0.33333 |
| 31-003 | 0.00000 | 0.08333 | 0.09091 | 0.00000 | 0.00000 | 0.00000 | 0.00000 | 0.00000 | 0.00000 | 0.00000 | 0.00000 | 0.00000 | 0.00000 | 0.00000 | 0.00000 |
| 31-004 | 0.00000 | 0.00000 | 0.00000 | 0.00000 | 0.00000 | 0.00000 | 0.00000 | 0.00000 | 0.00000 | 0.00000 | 0.00000 | 0.00000 | 0.00000 | 0.00000 | 0.16667 |
| 31-005 | 0.00000 | 0.00000 | 0.00000 | 0.00000 | 0.00000 | 0.09091 | 0.08333 | 0.00000 | 0.00000 | 0.00000 | 0.00000 | 0.00000 | 0.00000 | 0.00000 | 0.16667 |
| 31-006 | 0.00000 | 0.00000 | 0.00000 | 0.00000 | 0.00000 | 0.00000 | 0.00000 | 0.00000 | 0.00000 | 0.00000 | 0.00000 | 0.00000 | 0.00000 | 0.00000 | 0.16667 |
| 31-007 | 0.00000 | 0.00000 | 0.00000 | 0.00000 | 0.00000 | 0.00000 | 0.00000 | 0.00000 | 0.00000 | 0.00000 | 0.00000 | 0.00000 | 0.00000 | 0.10000 | 0.50000 |
| 31-008 | 0.00000 | 0.00000 | 0.00000 | 0.00000 | 0.00000 | 0.00000 | 0.00000 | 0.00000 | 0.00000 | 0.00000 | 0.00000 | 0.00000 | 0.00000 | 0.00000 | 0.16667 |
| 31-009 | 0.00000 | 0.00000 | 0.00000 | 0.00000 | 0.00000 | 0.00000 | 0.00000 | 0.00000 | 0.10000 | 0.33333 | 0.33333 | 0.40000 | 0.75000 | 0.90000 | 0.66667 |
| 31-010 | 0.00000 | 0.00000 | 0.00000 | 0.00000 | 0.00000 | 0.00000 | 0.00000 | 0.00000 | 0.00000 | 0.08333 | 0.00000 | 0.00000 | 0.00000 | 0.00000 | 0.00000 |
| 31-011 | 0.00000 | 0.08333 | 0.00000 | 0.00000 | 0.00000 | 0.00000 | 0.08333 | 0.00000 | 0.00000 | 0.33333 | 0.00000 | 0.00000 | 0.00000 | 0.10000 | 0.00000 |
| 31-012 | 0.00000 | 0.00000 | 0.09091 | 0.00000 | 0.00000 | 0.00000 | 0.08333 | 0.00000 | 0.00000 | 0.00000 | 0.00000 | 0.00000 | 0.00000 | 0.00000 | 0.00000 |
| 31-013 | 0.00000 | 0.00000 | 0.00000 | 0.00000 | 0.00000 | 0.00000 | 0.00000 | 0.00000 | 0.00000 | 0.08333 | 0.00000 | 0.00000 | 0.00000 | 0.00000 | 0.00000 |
| 31-014 | 0.00000 | 0.00000 | 0.00000 | 0.00000 | 0.00000 | 0.00000 | 0.00000 | 0.00000 | 0.00000 | 0.25000 | 0.00000 | 0.10000 | 0.12500 | 0.60000 | 0.66667 |
| 31-015 | 0.00000 | 0.00000 | 0.00000 | 0.00000 | 0.00000 | 0.09091 | 0.00000 | 0.00000 | 0.00000 | 0.00000 | 0.00000 | 0.00000 | 0.00000 | 0.10000 | 0.00000 |
| 31-016 | 0.00000 | 0.00000 | 0.00000 | 0.00000 | 0.00000 | 0.00000 | 0.00000 | 0.00000 | 0.00000 | 0.25000 | 0.00000 | 0.00000 | 0.00000 | 0.20000 | 0.00000 |
| 31-017 | 0.00000 | 0.00000 | 0.00000 | 0.00000 | 0.00000 | 0.00000 | 0.00000 | 0.00000 | 0.00000 | 0.00000 | 0.00000 | 0.10000 | 0.25000 | 0.50000 | 0.16667 |
| 31-018 | 0.00000 | 0.00000 | 0.00000 | 0.00000 | 0.00000 | 0.00000 | 0.00000 | 0.00000 | 0.00000 | 0.08333 | 0.00000 | 0.00000 | 0.12500 | 0.60000 | 0.00000 |
| 31-019 | 0.00000 | 0.00000 | 0.00000 | 0.08333 | 0.00000 | 0.00000 | 0.00000 | 0.00000 | 0.00000 | 0.00000 | 0.00000 | 0.00000 | 0.00000 | 0.00000 | 0.00000 |
| 31-020 | 0.00000 | 0.08333 | 0.00000 | 0.00000 | 0.00000 | 0.09091 | 0.00000 | 0.00000 | 0.00000 | 0.00000 | 0.00000 | 0.00000 | 0.00000 | 0.00000 | 0.00000 |
| 31-021 | 0.00000 | 0.08333 | 0.00000 | 0.00000 | 0.00000 | 0.09091 | 0.00000 | 0.00000 | 0.00000 | 0.00000 | 0.00000 | 0.10000 | 0.00000 | 0.00000 | 0.00000 |
| 31-022 | 0.00000 | 0.00000 | 0.18182 | 0.50000 | 0.08333 | 0.36364 | 0.25000 | 0.00000 | 0.00000 | 0.25000 | 0.22222 | 0.00000 | 0.25000 | 0.10000 | 0.00000 |
| 31-023 | 0.00000 | 0.00000 | 0.00000 | 0.00000 | 0.00000 | 0.00000 | 0.00000 | 0.00000 | 0.00000 | 0.00000 | 0.00000 | 0.00000 | 0.00000 | 0.10000 | 0.00000 |
| 31-024 | 0.00000 | 0.00000 | 0.00000 | 0.00000 | 0.00000 | 0.18182 | 0.00000 | 0.00000 | 0.10000 | 0.16667 | 0.00000 | 0.00000 | 0.00000 | 0.00000 | 0.16667 |
| 31-025 | 0.00000 | 0.00000 | 0.00000 | 0.00000 | 0.08333 | 0.00000 | 0.00000 | 0.00000 | 0.00000 | 0.16667 | 0.11111 | 0.10000 | 0.00000 | 0.00000 | 0.00000 |
| 31-026 | 0.00000 | 0.00000 | 0.00000 | 0.00000 | 0.00000 | 0.00000 | 0.00000 | 0.00000 | 0.00000 | 0.00000 | 0.11111 | 0.00000 | 0.37500 | 0.00000 | 0.00000 |
| 31-027 | 0.00000 | 0.00000 | 0.09091 | 0.16667 | 0.00000 | 0.09091 | 0.08333 | 0.00000 | 0.00000 | 0.00000 | 0.00000 | 0.00000 | 0.00000 | 0.00000 | 0.00000 |
| 31-028 | 0.00000 | 0.00000 | 0.00000 | 0.00000 | 0.00000 | 0.00000 | 0.08333 | 0.00000 | 0.00000 | 0.25000 | 0.00000 | 0.00000 | 0.12500 | 0.10000 | 0.00000 |
| 31-029 | 0.20000 | 0.00000 | 0.00000 | 0.00000 | 0.00000 | 0.00000 | 0.00000 | 0.00000 | 0.00000 | 0.00000 | 0.00000 | 0.00000 | 0.00000 | 0.00000 | 0.00000 |
| 31-030 | 0.00000 | 0.00000 | 0.00000 | 0.00000 | 0.00000 | 0.00000 | 0.00000 | 0.00000 | 0.00000 | 0.00000 | 0.00000 | 0.10000 | 0.00000 | 0.30000 | 0.00000 |
| 31-031 | 0.00000 | 0.50000 | 0.27273 | 0.08333 | 0.08333 | 0.09091 | 0.08333 | 0.50000 | 0.20000 | 0.41667 | 0.88889 | 0.60000 | 0.87500 | 0.20000 | 0.50000 |
| 31-032 | 0.00000 | 0.08333 | 0.00000 | 0.00000 | 0.00000 | 0.00000 | 0.00000 | 0.33333 | 0.00000 | 0.08333 | 0.00000 | 0.00000 | 0.12500 | 0.00000 | 0.00000 |
| 31-033 | 0.40000 | 0.75000 | 0.45455 | 0.50000 | 0.50000 | 0.00000 | 0.66667 | 0.33333 | 0.50000 | 0.91667 | 0.88889 | 1.00000 | 1.00000 | 0.90000 | 0.66667 |
| 31-034 | 0.20000 | 0.00000 | 0.00000 | 0.00000 | 0.08333 | 0.00000 | 0.00000 | 0.00000 | 0.00000 | 0.08333 | 0.00000 | 0.00000 | 0.00000 | 0.00000 | 0.00000 |
| 31-035 | 0.00000 | 0.00000 | 0.00000 | 0.08333 | 0.00000 | 0.00000 | 0.08333 | 0.00000 | 0.00000 | 0.00000 | 0.00000 | 0.00000 | 0.00000 | 0.00000 | 0.00000 |
| 31-036 | 0.00000 | 0.08333 | 0.09091 | 0.16667 | 0.08333 | 0.00000 | 0.08333 | 0.00000 | 0.00000 | 0.41667 | 0.11111 | 0.00000 | 0.12500 | 0.20000 | 0.16667 |
| 31-037 | 0.00000 | 0.00000 | 0.00000 | 0.00000 | 0.00000 | 0.00000 | 0.00000 | 0.00000 | 0.00000 | 0.00000 | 0.11111 | 0.20000 | 0.12500 | 0.00000 | 0.16667 |
| 31-038 | 0.00000 | 0.41667 | 0.36364 | 0.58333 | 0.08333 | 0.45455 | 0.16667 | 0.00000 | 0.00000 | 0.00000 | 0.00000 | 0.00000 | 0.00000 | 0.00000 | 0.00000 |
| 31-039 | 0.40000 | 0.50000 | 0.09091 | 0.08333 | 0.41667 | 0.18182 | 0.16667 | 0.08333 | 0.00000 | 0.00000 | 0.00000 | 0.00000 | 0.00000 | 0.00000 | 0.00000 |
| 31-040 | 0.00000 | 0.00000 | 0.00000 | 0.00000 | 0.16667 | 0.00000 | 0.00000 | 0.00000 | 0.00000 | 0.08333 | 0.00000 | 0.00000 | 0.00000 | 0.00000 | 0.00000 |
| 31-041 | 0.00000 | 0.00000 | 0.00000 | 0.00000 | 0.16667 | 0.00000 | 0.00000 | 0.00000 | 0.00000 | 0.00000 | 0.00000 | 0.00000 | 0.00000 | 0.00000 | 0.00000 |
| 31-042 | 0.20000 | 0.41667 | 0.36364 | 0.50000 | 0.16667 | 0.45455 | 0.16667 | 0.33333 | 0.10000 | 0.00000 | 0.00000 | 0.00000 | 0.00000 | 0.20000 | 0.00000 |
| 31-043 | 0.20000 | 0.50000 | 0.27273 | 0.50000 | 0.16667 | 0.45455 | 0.16667 | 0.25000 | 0.10000 | 0.08333 | 0.00000 | 0.00000 | 0.00000 | 0.00000 | 0.00000 |
| 31-044 | 0.80000 | 0.41667 | 0.54545 | 0.25000 | 0.58333 | 0.36364 | 0.75000 | 0.58333 | 0.60000 | 0.00000 | 0.00000 | 0.00000 | 0.00000 | 0.00000 | 0.00000 |
| 31-045 | 0.80000 | 0.41667 | 0.54545 | 0.25000 | 0.58333 | 0.36364 | 0.66667 | 0.58333 | 0.60000 | 0.00000 | 0.00000 | 0.00000 | 0.00000 | 0.00000 | 0.00000 |
| 31-046 | 0.00000 | 0.08333 | 0.00000 | 0.00000 | 0.00000 | 0.00000 | 0.00000 | 0.00000 | 0.00000 | 0.00000 | 0.00000 | 0.00000 | 0.00000 | 0.00000 | 0.00000 |
| 31-047 | 0.00000 | 0.00000 | 0.00000 | 0.00000 | 0.00000 | 0.00000 | 0.08333 | 0.00000 | 0.00000 | 0.00000 | 0.00000 | 0.00000 | 0.00000 | 0.00000 | 0.00000 |
| 31-048 | 0.20000 | 0.00000 | 0.09091 | 0.00000 | 0.08333 | 0.00000 | 0.00000 | 0.00000 | 0.00000 | 0.00000 | 0.00000 | 0.00000 | 0.00000 | 0.00000 | 0.00000 |
| 31-049 | 0.00000 | 0.08333 | 0.18182 | 0.25000 | 0.16667 | 0.09091 | 0.08333 | 0.00000 | 0.00000 | 0.00000 | 0.00000 | 0.00000 | 0.00000 | 0.10000 | 0.00000 |
| 31-050 | 0.00000 | 0.00000 | 0.00000 | 0.00000 | 0.00000 | 0.00000 | 0.08333 | 0.00000 | 0.00000 | 0.00000 | 0.00000 | 0.00000 | 0.00000 | 0.00000 | 0.00000 |
| 31-051 | 0.00000 | 0.00000 | 0.00000 | 0.00000 | 0.00000 | 0.00000 | 0.00000 | 0.00000 | 0.00000 | 0.08333 | 0.00000 | 0.00000 | 0.00000 | 0.00000 | 0.00000 |
| 31-052 | 0.00000 | 0.00000 | 0.00000 | 0.00000 | 0.00000 | 0.00000 | 0.00000 | 0.00000 | 0.00000 | 0.00000 | 0.00000 | 0.00000 | 0.00000 | 0.00000 | 0.00000 |
| 31-053 | 0.00000 | 0.00000 | 0.00000 | 0.00000 | 0.25000 | 0.00000 | 0.00000 | 0.00000 | 0.00000 | 0.00000 | 0.00000 | 0.00000 | 0.00000 | 0.00000 | 0.00000 |
| 31-054 | 0.00000 | 0.00000 | 0.09091 | 0.00000 | 0.00000 | 0.00000 | 0.08333 | 0.00000 | 0.00000 | 0.00000 | 0.00000 | 0.00000 | 0.00000 | 0.00000 | 0.00000 |
| 31-055 | 0.00000 | 0.08333 | 0.09091 | 0.25000 | 0.33333 | 0.09091 | 0.08333 | 0.00000 | 0.00000 | 0.25000 | 0.22222 | 0.00000 | 0.00000 | 0.10000 | 0.00000 |
| 31-056 | 0.00000 | 0.00000 | 0.00000 | 0.00000 | 0.00000 | 0.00000 | 0.00000 | 0.00000 | 0.00000 | 0.16667 | 0.00000 | 0.00000 | 0.00000 | 0.00000 | 0.00000 |
| 31-057 | 0.00000 | 0.00000 | 0.00000 | 0.00000 | 0.00000 | 0.00000 | 0.00000 | 0.00000 | 0.00000 | 0.08333 | 0.00000 | 0.00000 | 0.00000 | 0.00000 | 0.00000 |
| 31-058 | 0.00000 | 0.00000 | 0.00000 | 0.00000 | 0.00000 | 0.00000 | 0.00000 | 0.00000 | 0.00000 | 0.16667 | 0.00000 | 0.10000 | 0.00000 | 0.00000 | 0.00000 |
| 31-059 | 0.00000 | 0.00000 | 0.00000 | 0.00000 | 0.00000 | 0.00000 | 0.00000 | 0.00000 | 0.00000 | 0.16667 | 0.00000 | 0.10000 | 0.12500 | 0.00000 | 0.00000 |
| 31-060 | 0.00000 | 0.00000 | 0.00000 | 0.00000 | 0.00000 | 0.00000 | 0.00000 | 0.00000 | 0.00000 | 0.16667 | 0.22222 | 0.00000 | 0.00000 | 0.10000 | 0.00000 |
| 31-061 | 0.00000 | 0.00000 | 0.00000 | 0.00000 | 0.00000 | 0.00000 | 0.00000 | 0.00000 | 0.00000 | 0.00000 | 0.00000 | 0.00000 | 0.00000 | 0.00000 | 0.00000 |
| 31-062 | 0.00000 | 0.00000 | 0.00000 | 0.00000 | 0.00000 | 0.09091 | 0.00000 | 0.00000 | 0.00000 | 0.00000 | 0.00000 | 0.10000 | 0.00000 | 0.20000 | 0.00000 |
| 31-063 | 0.20000 | 0.08333 | 0.09091 | 0.25000 | 0.00000 | 0.09091 | 0.16667 | 0.00000 | 0.00000 | 0.00000 | 0.11111 | 0.10000 | 0.00000 | 0.00000 | 0.00000 |
| 31-064 | 0.20000 | 0.16667 | 0.09091 | 0.08333 | 0.00000 | 0.27273 | 0.08333 | 0.00000 | 0.00000 | 0.00000 | 0.11111 | 0.10000 | 0.12500 | 0.10000 | 0.00000 |
| 31-065 | 0.00000 | 0.00000 | 0.00000 | 0.08333 | 0.00000 | 0.18182 | 0.00000 | 0.00000 | 0.00000 | 0.08333 | 0.22222 | 0.00000 | 0.00000 | 0.10000 | 0.00000 |
| 31-066 | 0.00000 | 0.00000 | 0.00000 | 0.00000 | 0.00000 | 0.00000 | 0.00000 | 0.00000 | 0.20000 | 0.08333 | 0.22222 | 0.10000 | 0.12500 | 0.20000 | 0.00000 |
| 31-067 | 0.00000 | 0.00000 | 0.00000 | 0.00000 | 0.00000 | 0.00000 | 0.00000 | 0.00000 | 0.00000 | 0.00000 | 0.22222 | 0.00000 | 0.00000 | 0.00000 | 0.00000 |
| 31-068 | 0.00000 | 0.16667 | 0.09091 | 0.16667 | 0.08333 | 0.00000 | 0.16667 | 0.08333 | 0.20000 | 0.00000 | 0.00000 | 0.00000 | 0.00000 | 0.00000 | 0.00000 |
| 31-069 | 0.00000 | 0.16667 | 0.09091 | 0.16667 | 0.08333 | 0.09091 | 0.08333 | 0.08333 | 0.40000 | 0.00000 | 0.00000 | 0.00000 | 0.00000 | 0.00000 | 0.00000 |
| 31-070 | 0.00000 | 0.00000 | 0.00000 | 0.00000 | 0.08333 | 0.27273 | 0.00000 | 0.00000 | 0.20000 | 0.00000 | 0.00000 | 0.00000 | 0.00000 | 0.00000 | 0.00000 |
| 31-071 | 0.00000 | 0.00000 | 0.00000 | 0.00000 | 0.00000 | 0.00000 | 0.00000 | 0.00000 | 0.00000 | 0.08333 | 0.22222 | 0.00000 | 0.00000 | 0.00000 | 0.00000 |
| 31-072 | 0.00000 | 0.00000 | 0.00000 | 0.00000 | 0.00000 | 0.00000 | 0.00000 | 0.00000 | 0.00000 | 0.00000 | 0.00000 | 0.00000 | 0.00000 | 0.00000 | 0.00000 |
| 31-073 | 0.00000 | 0.00000 | 0.00000 | 0.00000 | 0.00000 | 0.00000 | 0.00000 | 0.00000 | 0.00000 | 0.00000 | 0.11111 | 0.00000 | 0.25000 | 0.20000 | 0.00000 |
| 31-074 | 0.00000 | 0.00000 | 0.00000 | 0.00000 | 0.00000 | 0.00000 | 0.00000 | 0.00000 | 0.00000 | 0.00000 | 0.00000 | 0.00000 | 0.00000 | 0.00000 | 0.16667 |
| 31-075 | 0.00000 | 0.00000 | 0.00000 | 0.00000 | 0.00000 | 0.00000 | 0.00000 | 0.00000 | 0.00000 | 0.16667 | 0.00000 | 0.00000 | 0.00000 | 0.00000 | 0.00000 |
| 31-076 | 0.00000 | 0.00000 | 0.00000 | 0.00000 | 0.00000 | 0.09091 | 0.00000 | 0.00000 | 0.00000 | 0.00000 | 0.00000 | 0.00000 | 0.00000 | 0.00000 | 0.00000 |
| 31-077 | 0.00000 | 0.00000 | 0.00000 | 0.00000 | 0.00000 | 0.00000 | 0.00000 | 0.00000 | 0.00000 | 0.00000 | 0.00000 | 0.00000 | 0.00000 | 0.10000 | 0.00000 |
| 31-078 | 0.00000 | 0.00000 | 0.00000 | 0.00000 | 0.00000 | 0.00000 | 0.00000 | 0.00000 | 0.00000 | 0.00000 | 0.11111 | 0.00000 | 0.50000 | 0.40000 | 0.00000 |
| 31-079 | 0.00000 | 0.00000 | 0.00000 | 0.00000 | 0.00000 | 0.00000 | 0.00000 | 0.00000 | 0.00000 | 0.25000 | 0.11111 | 0.10000 | 0.00000 | 0.00000 | 0.00000 |
| 31-080 | 0.00000 | 0.00000 | 0.00000 | 0.00000 | 0.00000 | 0.00000 | 0.00000 | 0.08333 | 0.00000 | 0.00000 | 0.00000 | 0.00000 | 0.00000 | 0.00000 | 0.00000 |
| 31-081 | 0.00000 | 0.00000 | 0.00000 | 0.00000 | 0.00000 | 0.00000 | 0.00000 | 0.00000 | 0.00000 | 0.00000 | 0.00000 | 0.00000 | 0.00000 | 0.00000 | 0.16667 |
| 31-082 | 0.00000 | 0.00000 | 0.00000 | 0.00000 | 0.00000 | 0.00000 | 0.00000 | 0.00000 | 0.00000 | 0.00000 | 0.11111 | 0.30000 | 0.00000 | 0.10000 | 0.00000 |
| 31-083 | 0.00000 | 0.00000 | 0.00000 | 0.00000 | 0.00000 | 0.00000 | 0.08333 | 0.00000 | 0.00000 | 0.00000 | 0.11111 | 0.00000 | 0.00000 | 0.00000 | 0.00000 |
| 31-084 | 0.00000 | 0.00000 | 0.00000 | 0.00000 | 0.00000 | 0.00000 | 0.00000 | 0.00000 | 0.00000 | 0.00000 | 0.00000 | 0.10000 | 0.00000 | 0.00000 | 0.00000 |
| 31-085 | 0.00000 | 0.00000 | 0.00000 | 0.00000 | 0.00000 | 0.00000 | 0.00000 | 0.00000 | 0.00000 | 0.00000 | 0.00000 | 0.10000 | 0.00000 | 0.00000 | 0.00000 |
| 31-086 | 0.00000 | 0.00000 | 0.00000 | 0.00000 | 0.00000 | 0.00000 | 0.08333 | 0.00000 | 0.00000 | 0.00000 | 0.22222 | 0.00000 | 0.25000 | 0.00000 | 0.00000 |
| 31-087 | 0.00000 | 0.00000 | 0.00000 | 0.00000 | 0.00000 | 0.00000 | 0.00000 | 0.00000 | 0.00000 | 0.00000 | 0.00000 | 0.00000 | 0.00000 | 0.10000 | 0.00000 |
| 31-088 | 0.00000 | 0.00000 | 0.00000 | 0.00000 | 0.00000 | 0.00000 | 0.08333 | 0.00000 | 0.00000 | 0.00000 | 0.00000 | 0.00000 | 0.00000 | 0.00000 | 0.00000 |
| 31-089 | 0.00000 | 0.00000 | 0.00000 | 0.00000 | 0.00000 | 0.00000 | 0.00000 | 0.00000 | 0.00000 | 0.00000 | 0.11111 | 0.00000 | 0.00000 | 0.00000 | 0.00000 |
| 31-090 | 0.00000 | 0.00000 | 0.00000 | 0.00000 | 0.00000 | 0.00000 | 0.00000 | 0.00000 | 0.00000 | 0.00000 | 0.00000 | 0.00000 | 0.00000 | 0.00000 | 0.16667 |
| 31-091 | 0.00000 | 0.00000 | 0.00000 | 0.00000 | 0.00000 | 0.09091 | 0.25000 | 0.00000 | 0.00000 | 0.00000 | 0.00000 | 0.00000 | 0.00000 | 0.00000 | 0.00000 |
| 31-092 | 0.00000 | 0.00000 | 0.00000 | 0.00000 | 0.00000 | 0.00000 | 0.41667 | 0.00000 | 0.00000 | 0.00000 | 0.00000 | 0.00000 | 0.00000 | 0.00000 | 0.16667 |
| 31-093 | 0.00000 | 0.00000 | 0.00000 | 0.00000 | 0.00000 | 0.00000 | 0.00000 | 0.00000 | 0.00000 | 0.00000 | 0.00000 | 0.00000 | 0.00000 | 0.00000 | 0.16667 |
| 33-001 | 0.00000 | 0.08333 | 0.00000 | 0.41667 | 0.00000 | 0.00000 | 0.10000 | 0.00000 | 0.00000 | 0.00000 | 0.00000 | 0.00000 | 0.00000 | 0.00000 | 0.33333 |
| 33-002 | 0.00000 | 0.08333 | 0.08333 | 0.25000 | 0.09091 | 0.11111 | 0.10000 | 0.00000 | 0.00000 | 0.00000 | 0.00000 | 0.00000 | 0.00000 | 0.00000 | 0.33333 |
| 33-003 | 0.00000 | 0.00000 | 0.00000 | 0.08333 | 0.09091 | 0.00000 | 0.00000 | 0.00000 | 0.00000 | 0.00000 | 0.00000 | 0.00000 | 0.14286 | 0.00000 | 0.00000 |
| 33-004 | 0.00000 | 0.16667 | 0.08333 | 0.58333 | 0.27273 | 0.22222 | 0.20000 | 0.12500 | 0.00000 | 0.00000 | 0.12500 | 0.00000 | 0.00000 | 0.00000 | 0.33333 |
| 33-005 | 0.00000 | 0.00000 | 0.00000 | 0.16667 | 0.00000 | 0.11111 | 0.10000 | 0.00000 | 0.00000 | 0.00000 | 0.00000 | 0.00000 | 0.00000 | 0.00000 | 0.00000 |
| 33-006 | 0.00000 | 0.00000 | 0.00000 | 0.00000 | 0.00000 | 0.00000 | 0.00000 | 0.00000 | 0.00000 | 0.00000 | 0.00000 | 0.00000 | 0.00000 | 0.00000 | 0.33333 |
| 33-007 | 0.00000 | 0.00000 | 0.00000 | 0.16667 | 0.00000 | 0.00000 | 0.00000 | 0.00000 | 0.00000 | 0.00000 | 0.00000 | 0.00000 | 0.00000 | 0.00000 | 0.00000 |
| 33-008 | 0.00000 | 0.00000 | 0.00000 | 0.08333 | 0.00000 | 0.00000 | 0.00000 | 0.00000 | 0.00000 | 0.00000 | 0.00000 | 0.00000 | 0.00000 | 0.00000 | 0.00000 |
| 33-009 | 0.00000 | 0.00000 | 0.00000 | 0.00000 | 0.00000 | 0.00000 | 0.00000 | 0.00000 | 0.00000 | 0.00000 | 0.00000 | 0.00000 | 0.00000 | 0.00000 | 0.33333 |
| 33-010 | 0.00000 | 0.00000 | 0.00000 | 0.08333 | 0.00000 | 0.00000 | 0.00000 | 0.00000 | 0.00000 | 0.00000 | 0.00000 | 0.00000 | 0.00000 | 0.00000 | 0.00000 |
| 33-011 | 0.00000 | 0.00000 | 0.16667 | 0.00000 | 0.09091 | 0.00000 | 0.00000 | 0.00000 | 0.00000 | 0.10000 | 0.00000 | 0.00000 | 0.00000 | 0.00000 | 0.00000 |
| 33-012 | 0.00000 | 0.00000 | 0.00000 | 0.00000 | 0.09091 | 0.11111 | 0.00000 | 0.12500 | 0.00000 | 0.10000 | 0.00000 | 0.00000 | 0.00000 | 0.00000 | 0.00000 |
| 33-013 | 0.00000 | 0.00000 | 0.00000 | 0.00000 | 0.00000 | 0.00000 | 0.00000 | 0.00000 | 0.00000 | 0.00000 | 0.00000 | 0.00000 | 0.28571 | 0.00000 | 0.00000 |
| 33-014 | 0.00000 | 0.00000 | 0.00000 | 0.00000 | 0.09091 | 0.00000 | 0.00000 | 0.00000 | 0.00000 | 0.00000 | 0.00000 | 0.00000 | 0.00000 | 0.00000 | 0.00000 |
| 33-015 | 0.00000 | 0.00000 | 0.00000 | 0.00000 | 0.00000 | 0.00000 | 0.00000 | 0.12500 | 0.00000 | 0.10000 | 0.00000 | 0.00000 | 0.00000 | 0.00000 | 0.00000 |
| 33-016 | 0.00000 | 0.41667 | 0.16667 | 0.58333 | 0.09091 | 0.00000 | 0.20000 | 0.00000 | 0.00000 | 0.40000 | 0.37500 | 0.00000 | 0.14286 | 0.00000 | 0.00000 |
| 33-017 | 0.00000 | 0.00000 | 0.00000 | 0.08333 | 0.00000 | 0.00000 | 0.00000 | 0.00000 | 0.00000 | 0.10000 | 0.00000 | 0.00000 | 0.00000 | 0.10000 | 0.00000 |
| 33-018 | 0.00000 | 0.16667 | 0.08333 | 0.16667 | 0.45455 | 0.22222 | 0.10000 | 0.75000 | 0.00000 | 0.40000 | 0.12500 | 0.00000 | 0.28571 | 0.30000 | 0.00000 |
| 33-019 | 0.00000 | 0.00000 | 0.00000 | 0.00000 | 0.00000 | 0.00000 | 0.00000 | 0.00000 | 0.00000 | 0.00000 | 0.00000 | 0.00000 | 0.00000 | 0.00000 | 0.33333 |
| 33-020 | 0.00000 | 0.08333 | 0.00000 | 0.08333 | 0.00000 | 0.00000 | 0.00000 | 0.00000 | 0.00000 | 0.00000 | 0.00000 | 0.00000 | 0.00000 | 0.00000 | 0.33333 |
| 33-021 | 0.00000 | 0.08333 | 0.00000 | 0.16667 | 0.09091 | 0.00000 | 0.00000 | 0.00000 | 0.11111 | 0.00000 | 0.37500 | 0.00000 | 0.00000 | 0.00000 | 0.33333 |
| 33-022 | 0.00000 | 0.00000 | 0.00000 | 0.00000 | 0.09091 | 0.22222 | 0.00000 | 0.00000 | 0.11111 | 0.00000 | 0.37500 | 0.00000 | 0.00000 | 0.00000 | 0.00000 |
| 33-023 | 0.00000 | 0.33333 | 0.08333 | 0.16667 | 0.27273 | 0.00000 | 0.10000 | 0.12500 | 0.00000 | 0.30000 | 0.12500 | 0.00000 | 0.14286 | 0.10000 | 0.00000 |
| 33-024 | 0.00000 | 0.25000 | 0.16667 | 0.33333 | 0.45455 | 0.11111 | 0.40000 | 0.25000 | 0.33333 | 0.80000 | 0.12500 | 0.00000 | 0.14286 | 0.40000 | 0.33333 |
| 33-025 | 0.00000 | 0.00000 | 0.00000 | 0.08333 | 0.00000 | 0.00000 | 0.00000 | 0.00000 | 0.00000 | 0.00000 | 0.00000 | 0.00000 | 0.00000 | 0.00000 | 0.00000 |
| 33-026 | 0.00000 | 0.00000 | 0.16667 | 0.16667 | 0.09091 | 0.00000 | 0.10000 | 0.00000 | 0.00000 | 0.10000 | 0.00000 | 0.00000 | 0.00000 | 0.00000 | 0.00000 |
| 33-027 | 0.00000 | 0.00000 | 0.08333 | 0.16667 | 0.09091 | 0.00000 | 0.20000 | 0.00000 | 0.00000 | 0.20000 | 0.00000 | 0.00000 | 0.00000 | 0.00000 | 0.00000 |
| 33-028 | 0.00000 | 0.00000 | 0.00000 | 0.00000 | 0.00000 | 0.00000 | 0.00000 | 0.00000 | 0.00000 | 0.10000 | 0.00000 | 0.00000 | 0.00000 | 0.00000 | 0.00000 |
| 33-029 | 0.00000 | 0.00000 | 0.00000 | 0.00000 | 0.09091 | 0.00000 | 0.00000 | 0.00000 | 0.00000 | 0.00000 | 0.00000 | 0.00000 | 0.00000 | 0.00000 | 0.00000 |
| 33-030 | 0.00000 | 0.00000 | 0.00000 | 0.00000 | 0.09091 | 0.00000 | 0.00000 | 0.00000 | 0.00000 | 0.00000 | 0.00000 | 0.00000 | 0.00000 | 0.00000 | 0.00000 |
| 33-031 | 0.00000 | 0.08333 | 0.00000 | 0.08333 | 0.00000 | 0.00000 | 0.10000 | 0.00000 | 0.00000 | 0.00000 | 0.00000 | 0.00000 | 0.00000 | 0.00000 | 0.00000 |
| 33-032 | 0.00000 | 0.16667 | 0.58333 | 0.33333 | 0.63636 | 0.00000 | 0.10000 | 0.00000 | 0.00000 | 0.00000 | 0.00000 | 0.00000 | 0.14286 | 0.30000 | 0.33333 |
| 33-033 | 0.33333 | 0.16667 | 0.16667 | 0.50000 | 0.45455 | 0.22222 | 0.00000 | 0.75000 | 0.22222 | 0.30000 | 0.12500 | 0.00000 | 0.28571 | 0.20000 | 0.00000 |
| 33-034 | 0.00000 | 0.08333 | 0.00000 | 0.00000 | 0.00000 | 0.00000 | 0.00000 | 0.00000 | 0.00000 | 0.30000 | 0.00000 | 0.00000 | 0.14286 | 0.00000 | 0.00000 |
| 33-035 | 0.00000 | 0.00000 | 0.00000 | 0.00000 | 0.09091 | 0.00000 | 0.00000 | 0.00000 | 0.00000 | 0.10000 | 0.00000 | 0.00000 | 0.00000 | 0.00000 | 0.00000 |
| 33-036 | 0.00000 | 0.00000 | 0.00000 | 0.00000 | 0.09091 | 0.00000 | 0.00000 | 0.00000 | 0.00000 | 0.00000 | 0.00000 | 0.00000 | 0.00000 | 0.00000 | 0.00000 |
| 33-037 | 0.00000 | 0.00000 | 0.08333 | 0.00000 | 0.09091 | 0.00000 | 0.00000 | 0.00000 | 0.00000 | 0.00000 | 0.00000 | 0.00000 | 0.00000 | 0.10000 | 0.00000 |
| 33-038 | 0.00000 | 0.00000 | 0.16667 | 0.08333 | 0.00000 | 0.00000 | 0.00000 | 0.00000 | 0.00000 | 0.00000 | 0.00000 | 0.00000 | 0.00000 | 0.10000 | 0.00000 |
| 33-039 | 0.00000 | 0.08333 | 0.08333 | 0.08333 | 0.45455 | 0.00000 | 0.00000 | 0.12500 | 0.00000 | 0.00000 | 0.12500 | 0.50000 | 0.14286 | 0.10000 | 0.00000 |
| 33-040 | 0.33333 | 0.00000 | 0.25000 | 0.16667 | 0.00000 | 0.33333 | 0.00000 | 0.12500 | 0.22222 | 0.20000 | 0.00000 | 0.00000 | 0.00000 | 0.20000 | 0.00000 |
| 33-041 | 0.00000 | 0.00000 | 0.00000 | 0.16667 | 0.00000 | 0.00000 | 0.10000 | 0.00000 | 0.00000 | 0.10000 | 0.12500 | 0.00000 | 0.00000 | 0.00000 | 0.00000 |
| 33-042 | 0.00000 | 0.00000 | 0.08333 | 0.00000 | 0.00000 | 0.00000 | 0.00000 | 0.00000 | 0.00000 | 0.00000 | 0.00000 | 0.00000 | 0.00000 | 0.00000 | 0.00000 |
| 33-043 | 0.00000 | 0.00000 | 0.00000 | 0.00000 | 0.09091 | 0.00000 | 0.00000 | 0.00000 | 0.00000 | 0.10000 | 0.00000 | 0.00000 | 0.00000 | 0.00000 | 0.00000 |
| 33-044 | 0.00000 | 0.00000 | 0.00000 | 0.08333 | 0.09091 | 0.00000 | 0.00000 | 0.00000 | 0.00000 | 0.00000 | 0.00000 | 0.00000 | 0.00000 | 0.00000 | 0.00000 |
| 33-045 | 0.00000 | 0.00000 | 0.00000 | 0.00000 | 0.18182 | 0.00000 | 0.00000 | 0.00000 | 0.00000 | 0.00000 | 0.00000 | 0.00000 | 0.00000 | 0.00000 | 0.00000 |
| 33-046 | 0.00000 | 0.00000 | 0.00000 | 0.00000 | 0.09091 | 0.00000 | 0.00000 | 0.00000 | 0.00000 | 0.00000 | 0.00000 | 0.00000 | 0.00000 | 0.00000 | 0.00000 |
| 33-047 | 0.00000 | 0.08333 | 0.00000 | 0.00000 | 0.09091 | 0.11111 | 0.00000 | 0.00000 | 0.00000 | 0.00000 | 0.00000 | 0.00000 | 0.00000 | 0.00000 | 0.00000 |
| 33-048 | 0.00000 | 0.00000 | 0.08333 | 0.00000 | 0.00000 | 0.00000 | 0.00000 | 0.00000 | 0.00000 | 0.00000 | 0.00000 | 0.00000 | 0.00000 | 0.00000 | 0.00000 |
| 33-049 | 0.00000 | 0.00000 | 0.08333 | 0.00000 | 0.00000 | 0.00000 | 0.00000 | 0.00000 | 0.11111 | 0.00000 | 0.00000 | 0.00000 | 0.00000 | 0.00000 | 0.00000 |
| 33-050 | 0.33333 | 0.00000 | 0.00000 | 0.00000 | 0.00000 | 0.00000 | 0.00000 | 0.00000 | 0.00000 | 0.10000 | 0.00000 | 0.00000 | 0.00000 | 0.00000 | 0.00000 |
| 33-051 | 0.00000 | 0.00000 | 0.00000 | 0.00000 | 0.00000 | 0.00000 | 0.00000 | 0.00000 | 0.00000 | 0.20000 | 0.12500 | 0.00000 | 0.00000 | 0.00000 | 0.00000 |
| 33-052 | 0.00000 | 0.00000 | 0.00000 | 0.00000 | 0.00000 | 0.00000 | 0.00000 | 0.00000 | 0.00000 | 0.10000 | 0.12500 | 0.00000 | 0.00000 | 0.00000 | 0.00000 |
| 33-053 | 0.00000 | 0.00000 | 0.16667 | 0.25000 | 0.00000 | 0.00000 | 0.00000 | 0.00000 | 0.00000 | 0.00000 | 0.00000 | 0.00000 | 0.00000 | 0.00000 | 0.00000 |
| 33-054 | 0.00000 | 0.08333 | 0.08333 | 0.25000 | 0.00000 | 0.00000 | 0.00000 | 0.12500 | 0.00000 | 0.00000 | 0.12500 | 0.00000 | 0.00000 | 0.10000 | 0.00000 |
| 33-055 | 0.00000 | 0.16667 | 0.25000 | 0.08333 | 0.00000 | 0.00000 | 0.00000 | 0.12500 | 0.00000 | 0.00000 | 0.00000 | 0.00000 | 0.00000 | 0.00000 | 0.33333 |
| 33-056 | 0.00000 | 0.25000 | 0.00000 | 0.08333 | 0.00000 | 0.11111 | 0.00000 | 0.00000 | 0.00000 | 0.20000 | 0.12500 | 0.00000 | 0.00000 | 0.10000 | 0.33333 |
| 33-057 | 0.00000 | 0.25000 | 0.00000 | 0.16667 | 0.00000 | 0.11111 | 0.10000 | 0.12500 | 0.00000 | 0.40000 | 0.00000 | 0.00000 | 0.00000 | 0.10000 | 0.33333 |
| 33-058 | 0.00000 | 0.41667 | 0.00000 | 0.16667 | 0.00000 | 0.11111 | 0.00000 | 0.12500 | 0.00000 | 0.30000 | 0.00000 | 0.00000 | 0.14286 | 0.10000 | 0.00000 |
| 33-059 | 0.00000 | 0.00000 | 0.00000 | 0.00000 | 0.00000 | 0.00000 | 0.20000 | 0.00000 | 0.00000 | 0.00000 | 0.00000 | 0.00000 | 0.00000 | 0.00000 | 0.33333 |
| 33-060 | 0.00000 | 0.16667 | 0.25000 | 0.08333 | 0.00000 | 0.00000 | 0.20000 | 0.00000 | 0.00000 | 0.10000 | 0.00000 | 0.00000 | 0.00000 | 0.00000 | 0.00000 |
| 33-061 | 0.00000 | 0.00000 | 0.00000 | 0.08333 | 0.00000 | 0.00000 | 0.00000 | 0.00000 | 0.00000 | 0.10000 | 0.00000 | 0.00000 | 0.00000 | 0.00000 | 0.00000 |
| 33-062 | 0.00000 | 0.16667 | 0.16667 | 0.00000 | 0.00000 | 0.00000 | 0.20000 | 0.00000 | 0.00000 | 0.30000 | 0.00000 | 0.00000 | 0.00000 | 0.00000 | 0.00000 |
| 33-063 | 0.00000 | 0.08333 | 0.00000 | 0.08333 | 0.00000 | 0.11111 | 0.00000 | 0.00000 | 0.00000 | 0.10000 | 0.00000 | 0.00000 | 0.00000 | 0.00000 | 0.00000 |
| 33-064 | 0.00000 | 0.08333 | 0.00000 | 0.08333 | 0.00000 | 0.00000 | 0.10000 | 0.00000 | 0.00000 | 0.10000 | 0.00000 | 0.00000 | 0.00000 | 0.00000 | 0.33333 |
| 33-065 | 0.00000 | 0.00000 | 0.16667 | 0.00000 | 0.00000 | 0.00000 | 0.10000 | 0.00000 | 0.00000 | 0.10000 | 0.00000 | 0.00000 | 0.00000 | 0.00000 | 0.33333 |
| 33-066 | 0.00000 | 0.16667 | 0.00000 | 0.08333 | 0.00000 | 0.00000 | 0.10000 | 0.00000 | 0.00000 | 0.00000 | 0.00000 | 0.00000 | 0.00000 | 0.10000 | 0.00000 |
| 33-067 | 0.00000 | 0.25000 | 0.00000 | 0.00000 | 0.00000 | 0.00000 | 0.10000 | 0.12500 | 0.00000 | 0.00000 | 0.00000 | 0.00000 | 0.00000 | 0.00000 | 0.00000 |
| 33-068 | 0.00000 | 0.08333 | 0.00000 | 0.00000 | 0.09091 | 0.00000 | 0.00000 | 0.00000 | 0.00000 | 0.00000 | 0.12500 | 0.00000 | 0.00000 | 0.00000 | 0.00000 |
| 33-069 | 0.00000 | 0.00000 | 0.00000 | 0.00000 | 0.00000 | 0.00000 | 0.00000 | 0.00000 | 0.00000 | 0.00000 | 0.12500 | 0.00000 | 0.00000 | 0.00000 | 0.00000 |
| 33-070 | 0.00000 | 0.00000 | 0.08333 | 0.00000 | 0.09091 | 0.00000 | 0.00000 | 0.00000 | 0.00000 | 0.00000 | 0.12500 | 0.00000 | 0.00000 | 0.00000 | 0.00000 |
| 33-071 | 0.00000 | 0.00000 | 0.08333 | 0.00000 | 0.00000 | 0.00000 | 0.00000 | 0.00000 | 0.00000 | 0.00000 | 0.12500 | 0.00000 | 0.00000 | 0.00000 | 0.00000 |
| 33-072 | 0.00000 | 0.16667 | 0.00000 | 0.00000 | 0.00000 | 0.00000 | 0.00000 | 0.00000 | 0.00000 | 0.10000 | 0.00000 | 0.00000 | 0.00000 | 0.00000 | 0.00000 |
| 33-073 | 0.00000 | 0.08333 | 0.00000 | 0.00000 | 0.00000 | 0.00000 | 0.00000 | 0.00000 | 0.00000 | 0.00000 | 0.25000 | 0.00000 | 0.00000 | 0.00000 | 0.00000 |
| 33-074 | 0.00000 | 0.08333 | 0.00000 | 0.00000 | 0.00000 | 0.00000 | 0.00000 | 0.12500 | 0.00000 | 0.00000 | 0.00000 | 0.00000 | 0.00000 | 0.00000 | 0.00000 |
| 33-075 | 0.00000 | 0.00000 | 0.00000 | 0.00000 | 0.00000 | 0.00000 | 0.00000 | 0.00000 | 0.00000 | 0.00000 | 0.00000 | 0.00000 | 0.00000 | 0.10000 | 0.00000 |
| 33-076 | 0.00000 | 0.00000 | 0.00000 | 0.16667 | 0.00000 | 0.00000 | 0.10000 | 0.12500 | 0.00000 | 0.00000 | 0.00000 | 0.00000 | 0.00000 | 0.00000 | 0.00000 |
| 33-077 | 0.00000 | 0.08333 | 0.00000 | 0.00000 | 0.00000 | 0.00000 | 0.00000 | 0.00000 | 0.00000 | 0.00000 | 0.00000 | 0.00000 | 0.00000 | 0.00000 | 0.00000 |
| 33-078 | 0.00000 | 0.08333 | 0.00000 | 0.00000 | 0.00000 | 0.00000 | 0.00000 | 0.00000 | 0.00000 | 0.00000 | 0.00000 | 0.00000 | 0.00000 | 0.00000 | 0.00000 |
| 33-079 | 0.00000 | 0.08333 | 0.00000 | 0.00000 | 0.00000 | 0.00000 | 0.00000 | 0.00000 | 0.00000 | 0.00000 | 0.00000 | 0.00000 | 0.00000 | 0.00000 | 0.00000 |
| 33-080 | 0.00000 | 0.00000 | 0.00000 | 0.00000 | 0.00000 | 0.11111 | 0.00000 | 0.00000 | 0.00000 | 0.00000 | 0.12500 | 0.00000 | 0.00000 | 0.00000 | 0.00000 |
| 33-081 | 0.00000 | 0.00000 | 0.00000 | 0.00000 | 0.00000 | 0.11111 | 0.00000 | 0.00000 | 0.00000 | 0.00000 | 0.12500 | 0.00000 | 0.00000 | 0.00000 | 0.00000 |
| 33-082 | 0.00000 | 0.00000 | 0.00000 | 0.00000 | 0.00000 | 0.00000 | 0.00000 | 0.00000 | 0.11111 | 0.00000 | 0.00000 | 0.00000 | 0.00000 | 0.00000 | 0.00000 |
| 33-083 | 0.00000 | 0.00000 | 0.00000 | 0.00000 | 0.00000 | 0.00000 | 0.00000 | 0.00000 | 0.11111 | 0.00000 | 0.00000 | 0.00000 | 0.00000 | 0.00000 | 0.00000 |
| 33-084 | 0.00000 | 0.16667 | 0.08333 | 0.00000 | 0.09091 | 0.11111 | 0.00000 | 0.12500 | 0.11111 | 0.10000 | 0.00000 | 0.00000 | 0.00000 | 0.20000 | 0.00000 |
| 33-085 | 0.00000 | 0.16667 | 0.08333 | 0.00000 | 0.09091 | 0.11111 | 0.00000 | 0.12500 | 0.11111 | 0.10000 | 0.00000 | 0.00000 | 0.00000 | 0.20000 | 0.00000 |
| 33-086 | 0.00000 | 0.16667 | 0.08333 | 0.08333 | 0.09091 | 0.00000 | 0.10000 | 0.12500 | 0.00000 | 0.20000 | 0.12500 | 0.00000 | 0.14286 | 0.00000 | 0.33333 |
| 33-087 | 0.00000 | 0.25000 | 0.08333 | 0.08333 | 0.09091 | 0.00000 | 0.10000 | 0.12500 | 0.00000 | 0.20000 | 0.12500 | 0.00000 | 0.14286 | 0.10000 | 0.33333 |
| 33-088 | 0.00000 | 0.00000 | 0.00000 | 0.00000 | 0.00000 | 0.00000 | 0.00000 | 0.00000 | 0.11111 | 0.00000 | 0.00000 | 0.00000 | 0.00000 | 0.00000 | 0.00000 |
| 33-089 | 0.00000 | 0.08333 | 0.00000 | 0.08333 | 0.00000 | 0.00000 | 0.00000 | 0.00000 | 0.00000 | 0.00000 | 0.12500 | 0.00000 | 0.00000 | 0.10000 | 0.00000 |
| 33-090 | 0.00000 | 0.08333 | 0.08333 | 0.41667 | 0.00000 | 0.33333 | 0.10000 | 0.00000 | 0.00000 | 0.10000 | 0.00000 | 0.00000 | 0.00000 | 0.10000 | 0.33333 |
| 33-091 | 0.00000 | 0.08333 | 0.00000 | 0.41667 | 0.00000 | 0.22222 | 0.10000 | 0.00000 | 0.00000 | 0.10000 | 0.00000 | 0.00000 | 0.00000 | 0.10000 | 0.33333 |
| 33-092 | 0.00000 | 0.16667 | 0.33333 | 0.16667 | 0.54545 | 0.11111 | 0.00000 | 0.25000 | 0.44444 | 0.10000 | 0.12500 | 0.50000 | 0.00000 | 0.00000 | 0.00000 |
| 33-093 | 0.00000 | 0.16667 | 0.25000 | 0.16667 | 0.45455 | 0.11111 | 0.00000 | 0.25000 | 0.44444 | 0.10000 | 0.12500 | 0.50000 | 0.00000 | 0.00000 | 0.00000 |
| 33-094 | 0.33333 | 0.33333 | 0.16667 | 0.00000 | 0.18182 | 0.00000 | 0.00000 | 0.00000 | 0.00000 | 0.10000 | 0.00000 | 0.00000 | 0.14286 | 0.00000 | 0.00000 |
| 33-095 | 0.33333 | 0.33333 | 0.16667 | 0.08333 | 0.18182 | 0.00000 | 0.10000 | 0.00000 | 0.00000 | 0.10000 | 0.00000 | 0.00000 | 0.28571 | 0.20000 | 0.00000 |
| 33-096 | 0.00000 | 0.00000 | 0.00000 | 0.08333 | 0.00000 | 0.00000 | 0.00000 | 0.00000 | 0.00000 | 0.00000 | 0.00000 | 0.00000 | 0.14286 | 0.20000 | 0.00000 |
| 33-097 | 0.00000 | 0.25000 | 0.08333 | 0.25000 | 0.09091 | 0.22222 | 0.30000 | 0.00000 | 0.00000 | 0.00000 | 0.12500 | 0.00000 | 0.00000 | 0.00000 | 0.00000 |
| 33-098 | 0.00000 | 0.16667 | 0.08333 | 0.25000 | 0.09091 | 0.22222 | 0.30000 | 0.00000 | 0.00000 | 0.00000 | 0.12500 | 0.00000 | 0.00000 | 0.20000 | 0.00000 |
| 33-099 | 0.00000 | 0.16667 | 0.00000 | 0.00000 | 0.09091 | 0.00000 | 0.00000 | 0.00000 | 0.00000 | 0.20000 | 0.12500 | 0.00000 | 0.00000 | 0.30000 | 0.00000 |
| 33-100 | 0.00000 | 0.08333 | 0.00000 | 0.00000 | 0.00000 | 0.00000 | 0.00000 | 0.00000 | 0.00000 | 0.10000 | 0.00000 | 0.00000 | 0.00000 | 0.00000 | 0.00000 |
| 33-101 | 0.00000 | 0.08333 | 0.00000 | 0.16667 | 0.00000 | 0.11111 | 0.00000 | 0.00000 | 0.00000 | 0.10000 | 0.12500 | 0.00000 | 0.00000 | 0.30000 | 0.00000 |
| 33-102 | 0.00000 | 0.00000 | 0.00000 | 0.16667 | 0.00000 | 0.11111 | 0.00000 | 0.00000 | 0.00000 | 0.10000 | 0.12500 | 0.00000 | 0.00000 | 0.30000 | 0.00000 |
| 33-103 | 0.00000 | 0.41667 | 0.00000 | 0.08333 | 0.00000 | 0.11111 | 0.00000 | 0.00000 | 0.00000 | 0.00000 | 0.00000 | 0.00000 | 0.00000 | 0.10000 | 0.00000 |
| 33-104 | 0.00000 | 0.41667 | 0.00000 | 0.08333 | 0.00000 | 0.11111 | 0.00000 | 0.00000 | 0.00000 | 0.00000 | 0.00000 | 0.00000 | 0.00000 | 0.10000 | 0.00000 |
| 33-105 | 0.00000 | 0.08333 | 0.00000 | 0.00000 | 0.09091 | 0.22222 | 0.10000 | 0.00000 | 0.00000 | 0.00000 | 0.25000 | 0.00000 | 0.14286 | 0.10000 | 0.00000 |
| 33-106 | 0.00000 | 0.08333 | 0.08333 | 0.00000 | 0.09091 | 0.22222 | 0.10000 | 0.00000 | 0.00000 | 0.00000 | 0.25000 | 0.00000 | 0.14286 | 0.10000 | 0.00000 |
| 33-107 | 0.00000 | 0.41667 | 0.41667 | 0.33333 | 0.18182 | 0.00000 | 0.40000 | 0.12500 | 0.00000 | 0.20000 | 0.12500 | 0.00000 | 0.00000 | 0.00000 | 0.00000 |
| 33-108 | 0.00000 | 0.41667 | 0.41667 | 0.33333 | 0.18182 | 0.00000 | 0.40000 | 0.12500 | 0.00000 | 0.20000 | 0.00000 | 0.00000 | 0.14286 | 0.00000 | 0.00000 |
| 33-109 | 0.00000 | 0.00000 | 0.00000 | 0.00000 | 0.00000 | 0.00000 | 0.00000 | 0.00000 | 0.00000 | 0.00000 | 0.00000 | 0.00000 | 0.00000 | 0.00000 | 0.00000 |
| 33-110 | 0.00000 | 0.00000 | 0.00000 | 0.00000 | 0.00000 | 0.00000 | 0.00000 | 0.00000 | 0.00000 | 0.10000 | 0.00000 | 0.00000 | 0.14286 | 0.20000 | 0.00000 |
| 33-111 | 0.00000 | 0.00000 | 0.00000 | 0.00000 | 0.00000 | 0.00000 | 0.00000 | 0.00000 | 0.00000 | 0.10000 | 0.00000 | 0.00000 | 0.14286 | 0.20000 | 0.00000 |
