# Supplementary material for: Landscape genomics reveal that ecological character determines adaptation: a case study in smoke tree (Cotinus coggygria Scop.)
Source: BMC Evol Biol. 2017 Aug 23;17:202. doi: 10.1186/s12862-017-1055-3 (PMC5569454; doi:10.1186/s12862-017-1055-3)
Supplement: Supplementary file 3 — Environmental variables for each location from the WorldClim database. (DOCX 15 kb) [file 12862_2017_1055_MOESM3_ESM.docx]

**Additional file 3** Environmental variables for each location from the WorldClim database.

| Population no. and code | Environmental variables | | | | | | | | | | | | | | | | | | |
| --- | --- | --- | --- | --- | --- | --- | --- | --- | --- | --- | --- | --- | --- | --- | --- | --- | --- | --- | --- |
|  | Bio1 | Bio2 | Bio3 | Bio4 | Bio5 | Bio6 | Bio7 | Bio8 | Bio9 | Bio10 | Bio11 | Bio12 | Bio13 | Bio14 | Bio15 | Bio16 | Bio17 | Bio18 | Bio19 |
| 1.HBWD | 11.9 | 9.7 | 29.4 | 854.2 | 27.8 | -5.1 | 32.9 | 21.3 | 1.1 | 22.4 | 1.1 | 970.0 | 173.0 | 16.0 | 65.8 | 448.0 | 55.0 | 431.0 | 55.0 |
| 2.HNSM | 11.9 | 11.3 | 30.5 | 976.0 | 29.4 | -7.7 | 37.1 | 22.1 | -0.7 | 23.6 | -0.7 | 724.0 | 164.0 | 10.0 | 80.7 | 387.0 | 36.0 | 373.0 | 36.0 |
| 3.SDBD | 13.1 | 10.2 | 28.1 | 1012.9 | 30.3 | -5.9 | 36.2 | 25.2 | 0.0 | 25.2 | 0.0 | 791.0 | 224.0 | 11.0 | 101.3 | 494.0 | 41.0 | 494.0 | 41.0 |
| 4.HNJL | 11.2 | 12.0 | 30.5 | 1013.5 | 29.6 | -9.6 | 39.2 | 21.6 | -2.2 | 23.3 | -2.2 | 655.0 | 170.0 | 7.0 | 97.2 | 388.0 | 24.0 | 386.0 | 24.0 |
| 5.SDYM | 12.8 | 11.5 | 29.3 | 1062.0 | 31.2 | -7.9 | 39.1 | 25.3 | -1.0 | 25.3 | -1.0 | 689.0 | 217.0 | 8.0 | 113.1 | 450.0 | 28.0 | 450.0 | 28.0 |
| 6.SXLK | 6.0 | 11.7 | 30.1 | 989.4 | 24.2 | -14.5 | 38.7 | 16.3 | -7.0 | 17.8 | -7.0 | 599.0 | 147.0 | 5.0 | 95.9 | 356.0 | 19.0 | 347.0 | 19.0 |
| 7.HNLJ | 11.6 | 11.0 | 30.7 | 928.3 | 28.6 | -7.3 | 35.9 | 21.5 | -0.3 | 22.9 | -0.3 | 766.0 | 163.0 | 11.0 | 75.3 | 390.0 | 38.0 | 372.0 | 38.0 |
| 8.SXTB | 0.7 | 7.1 | 25.1 | 755.7 | 14.6 | -13.8 | 28.4 | 9.1 | -8.8 | 10.0 | -8.8 | 1010.0 | 182.0 | 10.0 | 77.0 | 519.0 | 35.0 | 469.0 | 35.0 |
| 9.SXTT | 11.0 | 9.0 | 27.0 | 885.7 | 27.6 | -5.7 | 33.3 | 20.4 | -0.3 | 21.8 | -0.3 | 732.0 | 134.0 | 5.0 | 80.3 | 391.0 | 20.0 | 336.0 | 20.0 |
| 10.SXLJ | 10.3 | 9.0 | 26.2 | 926.0 | 26.8 | -7.6 | 34.4 | 20.1 | -1.6 | 21.6 | -1.6 | 726.0 | 143.0 | 9.0 | 73.4 | 364.0 | 31.0 | 335.0 | 31.0 |
| 11.SXWL | 10.1 | 9.4 | 26.5 | 963.6 | 27.0 | -8.6 | 35.6 | 20.3 | -2.3 | 21.8 | -2.3 | 631.0 | 128.0 | 8.0 | 77.4 | 329.0 | 26.0 | 304.0 | 26.0 |
| 12.HNYT | 13.7 | 12.0 | 30.6 | 1022.2 | 32.2 | -7.0 | 39.2 | 24.1 | 0.3 | 25.9 | 0.3 | 597.0 | 152.0 | 6.0 | 95.4 | 350.0 | 21.0 | 347.0 | 21.0 |
| 13.SXHM | 10.0 | 10.3 | 27.5 | 996.7 | 27.7 | -9.8 | 37.5 | 20.4 | -2.9 | 22.0 | -2.9 | 567.0 | 118.0 | 5.0 | 84.5 | 314.0 | 17.0 | 289.0 | 17.0 |
| 14.SXTL | 10.2 | 13.6 | 31.6 | 1084.4 | 30.2 | -12.8 | 43.0 | 21.4 | -3.9 | 23.0 | -3.9 | 433.0 | 115.0 | 4.0 | 104.6 | 273.0 | 13.0 | 264.0 | 13.0 |
| 15.HBTG | 10.4 | 12.7 | 30.6 | 1093.1 | 29.5 | -12.0 | 41.5 | 23.2 | -3.8 | 23.2 | -3.8 | 462.0 | 131.0 | 2.0 | 116.9 | 309.0 | 12.0 | 309.0 | 12.0 |
